# Targeting PTPN13 with 11-amino-acid peptides of C-terminal APC prevents immune evasion of colorectal cancer

**DOI:** 10.1038/s41422-025-01206-4

**Published:** 2026-01-05

**Authors:** Wen-Hui Ma, Wen-Yi Li, Tao Chen, Linqian Jing, Yue-Hong Chen, Kejun Li, Zhuo-Luo Xu, Rong-Fang Shen, Yutong He, Tingyu Mou, Ting-Yue Luo, Xiangnan Sun, Zhao-Kun Wu, Li-Jing Wang, Hong-Juan Liu, Xiaozhong Qiu, Yi Gao, Xiaochun Bai, Wei Wang, Dalei Wu, Guoxin Li, Wei-Jie Zhou

**Affiliations:** 1 State Key Laboratory of Multi-organ Injury Prevention and Treatment, Guangdong Provincial Key Laboratory of Precision Medicine for Gastrointestinal Tumor, Department of General Surgery, Nanfang Hospital, Southern Medical University, Guangzhou, Guangdong, China; 2 Cancer Research Institute, School of Basic Medical Sciences, Southern Medical University, Guangzhou, Guangdong, China; 3 Department of Gastrointestinal Surgery, The First Affiliated Hospital of Guangzhou University of Chinese Medicine, Guangzhou, Guangdong, China; 4 Department of Gastrointestinal and hernia surgery, Ganzhou Hospital-Nanfang Hospital, Southern Medical University, Ganzhou, Jiangxi China; 5 State Key Laboratory of Microbial Technology, Shandong University, Qingdao, Shandong China; 6 State Key Laboratory of Molecular Oncology, Department of Etiology and Carcinogenesis, National Cancer Center, National Clinical Research Center for Cancer, Cancer Hospital, Chinese Academy of Medical Sciences and Peking Union Medical College, Beijing, China; 7 The Fifth Affiliated Hospital of Southern Medical University, Guangdong Provincial Key Laboratory of Construction and Detection in Tissue Engineering, Southern Medical University, Guangzhou, Guangdong, China; 8 Vascular Biology Research Institute, School of Life Sciences and Biopharmaceuticals, Guangdong Pharmaceutical University, Guangzhou, Guangdong, China; 9 General Surgery Center, Department of Hepatobiliary Surgery II, Guangdong Provincial Research Center for Artificial Organ and Tissue Engineering, Guangzhou Clinical Research and Transformation Center for Artificial Liver, Institute of Regenerative Medicine, Zhujiang Hospital, Southern Medical University, Guangzhou, Guangdong, China; 10 State Key Laboratory of Multi-organ Injury Prevention and Treatment, Department of Cell Biology, School of Basic Medical Sciences, Southern Medical University, Guangzhou, Guangdong, China; 11 Division of Nephrology, Guangdong Provincial People’s Hospital (Guangdong Academy of Medical Sciences), Southern Medical University, Guangzhou, Guangdong, China

**Keywords:** Colorectal cancer, Immunoediting, Cancer immunotherapy

## Abstract

Colorectal cancer (CRC) remains largely refractory to immune-checkpoint blockade, with *adenomatous polyposis coli* (*APC*) mutations present in 80%–90% of cases. Loss of APC was previously thought to promote tumor progression mainly through deregulated Wnt/β-catenin signaling. Here, we report that APC loss leads to inhibition of CD8^+^ T cell infiltration and CRC immune evasion through the dephosphorylation of signal transducers and activators of transcription 1 (STAT1) by protein tyrosine phosphatase non-receptor type 13 (PTPN13), independently of β-catenin. Peptides containing the last 11 C-terminal amino acid (aa) residues of APC (APC11) bind directly to PTPN13 to block PTPN13–STAT1 interactions and facilitate STAT1 phosphorylation, interferon regulatory factor-1 (IRF1) expression, major histocompatibility complex (MHC) class I antigen presentation, and T cell intratumoral infiltration, all of which eventually inhibit tumor progression and enhance the effects of programmed cell death 1 (PD1) blockade. Thus, we have identified a previously unknown APC/PTPN13/STAT1-dependent tumor immune-suppressive mechanism. The potent tumor-suppressing effect of combining anti-PD1 antibodies with APC11 peptides provides a compelling target and rationale for future development of anti-tumor drugs for patients with CRC.

## Introduction

Colorectal cancer (CRC) is the third most commonly diagnosed cancer and the second leading cause of cancer-related mortality worldwide.^1,2^ Despite recent advances in immunotherapies that have revolutionized cancer treatment, their effectiveness in CRC remains limited.^3,4^ Although CRC characterized by microsatellite instability-high (MSI-H) responds favorably to PD1 blockade, this subset represents less than 15% of all CRC cases. The vast majority of CRC patients, characterized by microsatellite stable (MSS) tumors, fail to derive significant benefit from these therapies, leaving an urgent unmet need for novel treatment strategies.^5–13^ The precise mechanisms that underlie immune evasion in CRC remain poorly defined.

Mutations in the tumor suppressor gene *adenomatous polyposis coli* (*APC*) occur in ~80%–90% of CRC cases.^14–19^ APC is a key regulator of the Wnt/β-catenin signaling pathway, and loss of APC function is a critical driver of colorectal tumorigenesis. Recent studies have revealed the complexity of APC-mutant CRC, demonstrating that APC-driven polyclonality markedly enhances tumorigenic potential and enables tumors to overcome growth constraints typically encountered by monoclonal populations.^20^ In cells with truncated APC, aberrant activation of cholesterol biosynthesis has been observed. The small molecule TASIN-1 (truncated APC selective inhibitor-1) selectively induces cytotoxicity in such cells by inhibiting cholesterol biosynthesis.^21^ Furthermore, elevated cholesterol levels in the inner leaflet of the plasma membrane (IPM) of APC-truncated CRC cells promote Wnt-independent assembly of Wnt signalosomes via interaction with Dishevelled (Dvl). Inhibitors that target the cholesterol–Dvl interaction effectively suppress β-catenin signaling and reduce viability in APC-truncated CRC cells.^22^ However, the role of APC mutations in mediating immune evasion remains poorly understood.

Here, we identify protein tyrosine phosphatase non-receptor type 13 (PTPN13) as a critical mediator of APC-driven immune evasion. PTPN13, previously known for its controversial roles in different tumors,^23^ is revealed here to dephosphorylate STAT1, thereby impairing the IFNγ-STAT1-IRF1-MHC class I antigen presentation pathway. This dephosphorylation limits CD8^+^ T cell infiltration and promotes tumor immune escape. APC directly interacts with PTPN13, blocking its interaction with STAT1 and thereby preventing STAT1 dephosphorylation by PTPN13. Notably, we demonstrate that APC-derived peptides, particularly the C-terminal 11 amino acids (APC11), effectively block the PTPN13–STAT1 interaction, restoring STAT1 phosphorylation and reactivating immune responses against tumors. Furthermore, an improved tumor-suppressing effect is observed when APC11 peptides are combined with anti-PD1 antibodies.

## Results

### Loss of APC drives immune evasion in CRC by reducing CD8^+^ T cell infiltration

**Fig. 1:**
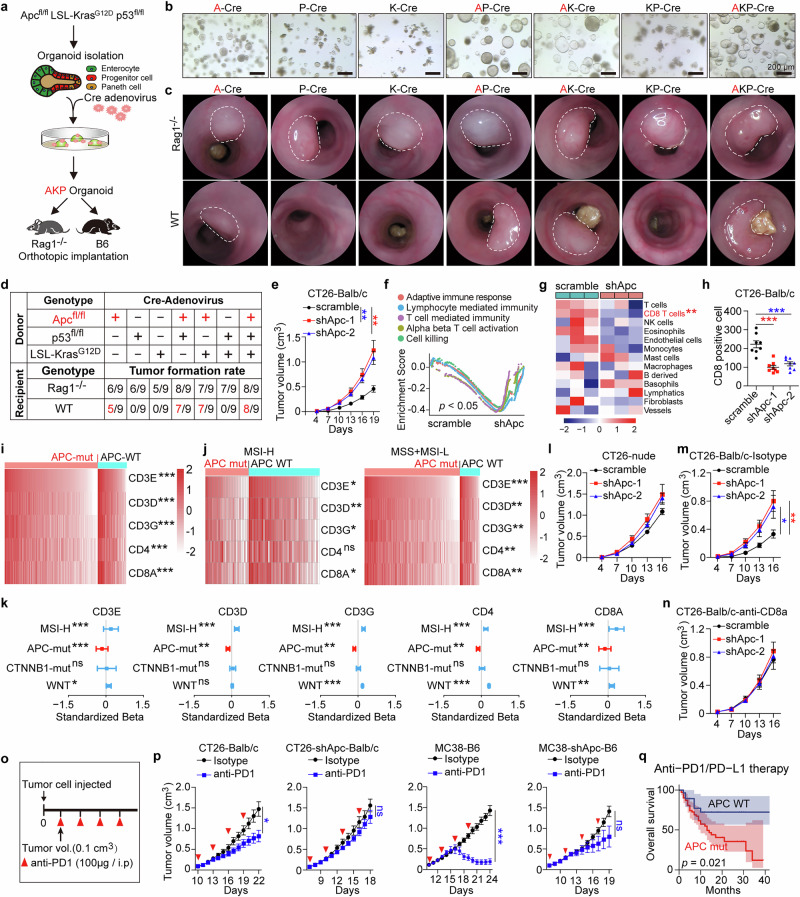
APC loss leads to CRC immune evasion and invalidation of anti-PD1 in tumor therapy. **a** Schematics showing how intestinal organoids from *Apc*^*fl/fl*^*/*LSL-*Kras*^G12D^*/p53*^fl/fl^ (AKP) mice were transfected with adenovirus-Cre and orthotopically injected into *Rag1*^–/–^ or wild-type (WT) C57/B6 mice. **b** Organoid cultures of intestinal crypts isolated from *Apc*^*fl/fl*^ (A), *p53*^*fl/fl*^ (P), *LSL-Kras*^*G12D*^ (K), *Apc*^*fl/fl*^*/p53*^*fl/fl*^ (AP), *Apc*^*fl/fl*^*/Kras*^*G12D*^ (AK), *p53*^*fl/fl*^*/Kras*^*G12D*^ (KP), and *Apc*^*fl/fl*^*/p53*^*fl/fl*^*/Kras*^*G12D*^ (AKP) mice were transfected with adenovirus expressing Cre recombinase. **c** Optical colonoscopy of orthotopic tumors of the indicated intestinal organoids injected into *Rag1*^–/–^ or WT C57/B6 mice. **d** Tumor formation rates of the indicated intestinal organoids injected into *Rag1*^–/–^ or WT C57/B6 mice. **e** Two independent Apc-silenced CT26 cells or scramble shRNA transfected cells were injected subcutaneously into WT Balb/c mice, and tumor growth was monitored at the indicated times. *n* = 8 per group, two-way ANOVA. **f** GSEA using GO pathways was performed between Apc-silenced and control groups of the indicated tumors in (**e**), using gene sets associated with immune function and T cell activity. **g** Microenvironment Cell Populations-counter (mMCPcounter) analysis performed between Apc-silenced and control groups of the indicated tumors in (**e**). Unpaired *t* test. **h** Scatter plot showing numbers of CD8 positive cells in Apc-silenced CT26 cells or scramble shRNA transfected CT26 subcutaneous tumors in WT Balb/c mice. *n* = 8 per group, one-way ANOVA. **i, j** Heat maps of all (**i**) and indicated subtypes (**j**) of colorectal cancer clustered in APC-WT vs APC-mutation gene groups in TCGA data. **k** Forest plots showing regression coefficients (Beta values) and 95% confidence intervals for the effects of MSI status and APC mutation on expression of CD3E, CD3D, CD3G, CD4, and CD8A in TCGA data. Linear regression analysis. **l** Two independent Apc-silenced CT26 cells or scramble shRNA transfected cells were injected subcutaneously into nude mice, and tumor growth was monitored at the indicated times. *n* = 8 per group, two-way ANOVA. **m, n** The indicated CT26 cells were transplanted into Balb/c mice injected with isotype or anti-CD8 antibody, and tumor growth was monitored. Significance of differences in tumor growth kinetics was assessed by two-way ANOVA. **o** Schematic of anti-PD1 antibody administration when the tumor reached 100 mm^3^, once every three days for four times in total. **p** The indicated cells were injected subcutaneously (1 × 10^7^ cells) into mice, anti-PD1 antibody was administered when the tumor reached 100 mm^3^, and tumor growth was monitored. *n* = 8 per group, two-way ANOVA. *n* = 6 per group, one-way ANOVA. **q** Survival analysis of APC WT and APC mut groups from MSKCC-CRC patients receiving anti-PD1/PD-L1 immunotherapy. Log-rank test. All data are mean ± SEM, *P < 0.05, **P < 0.01, ***P < 0.001.

*APC* (~80%–90%), *TP53* (~50%–60%), and *KRAS (*~40%–50%) are the three most frequently mutated genes in CRC, collaborating to drive tumor progression.^14–16^ To investigate their respective roles in tumor immune evasion, we employed genetically engineered mice carrying conditional null alleles of *Apc* allele (*Apc*^*fl/fl*^), *p53* (*Trp53*^*fl/fl*^, hereafter *p53*^*fl/fl*^), and mutant *Kras* (*LSL-Kras*^*G12D*^, hereafter *Kras*^*G12D*^). Crossbreeding generated *Apc*^*fl/fl*^*/p53*^*fl/fl*^, *Apc*^*fl/fl*^*/Kras*^*G12D*^, *p53*^*fl/fl*^*/Kras*^*G12D*^, and *Apc*^*fl/fl*^*/p53*^*fl/fl*^*/Kras*^*G12D*^ models. Intestinal crypts isolated from these mice formed organoids^24^ with quantified protein expression of APC, *Kras*^*G12D*^, and p53 (Fig. 1a, b; Supplementary information, Fig. S1a). When inoculated into the colonic mucosa of wild-type and *Rag1*^*–/–*^ immunodeficient mice (lacking T and B cells), tumors developed in immunodeficient mice with p53 loss, *Kras*^*G12D*^ mutation, or both, whereas Apc loss allowed tumor formation in both WT and immunodeficient mice (Fig. 1c, d; Supplementary information, Fig. S1b, c). These results indicate that Apc loss, rather than p53 loss or *Kras*^*G12D*^ mutation, is a critical driver of immune evasion during early CRC tumorigenesis.

To further validate the role of APC in CRC immune evasion, we employed a syngeneic colon cancer model using shRNA-mediated Apc knockdown in CT26 cells (Supplementary information, Fig. S2a). APC knockdown increased the growth of subcutaneously grafted CT26 cells in WT Balb/c mice (Fig. 1e; Supplementary information, Fig. S2b). Gene set enrichment analysis (GSEA) using gene ontology (GO) pathways and Microenvironment Cell Populations-counter (mMCPcounter) analysis of RNA sequencing (RNA-seq) data revealed significant suppression of T cell-mediated immunity in CT26-shApc tumors compared with CT26 control-shRNA tumors (Fig. 1f, g). Examination of immunofluorescence confirmed reduced CD8^+^ T cell infiltration in CT26-shApc tumors compared with control tumors (Fig. 1h; Supplementary information, Fig. S2c).

Analysis of data from The Cancer Genome Atlas (TCGA) showed significantly lower expression of T-cell markers, including CD3E, CD3D, CD3G, CD4, and CD8A, in APC-mutant tumors compared with APC-WT tumors (Fig. 1i). Stratification of tumors into MSI-High (MSI-H) and a combined MSI-Low (MSI-L) plus Microsatellite Stable (MSS) group revealed consistently lower T-cell marker expression in APC-mutant tumors across both groups (Fig. 1j). Linear regression analysis incorporating MSI status, APC and CTNNB1 mutations, and Wnt activity revealed a significant negative correlation between APC mutations and immune markers (CD3E, CD3G, CD4, and CD8A), together with significant positive correlations of MSI status and Wnt activity with these same immune markers. By contrast, CTNNB1 mutations did not substantially impact immune marker expression (Fig. 1k). These analyses indicated that the reduced expression of T-cell markers in APC-mutant tumors was independent of the Wnt/β-catenin signaling pathway.

In our own CRC cohort, APC mutant tumors showed significantly reduced CD3^+^ and CD8^+^ T cell infiltration compared with APC-WT tumors across both MSI-H and MSS + MSI-L groups (Supplementary information, Fig. S2d–g). Further regression analysis confirmed these findings, identifying MSI-H status as a positive independent variable and APC mutations as a negative independent variable for CD3^+^ and CD8^+^ T cell counts (Supplementary information, Fig. S2h).

To assess the importance of CD8^+^ T cells in Apc knockdown-induced tumor promotion, CT26 cells were subcutaneously grafted into immunodeficient nude mice, where APC knockdown did not affect tumor growth (Fig. 11; Supplementary information, Fig. S2i). Depletion of CD8^+^ T cells confirmed their crucial role in Apc knockdown-induced tumor growth, as tumor growth was unaffected in mice treated with anti-CD8 antibodies (Fig. 1m, n; Supplementary information, Fig. S2j, k). Similar results were observed in MC38 colon cancer cells: APC knockdown increased tumor growth and reduced CD8^+^ T cell infiltration in WT C57BL/6 mice but had no effect in *Rag1*^*–/–*^ mice (C57BL/6 background) (Supplementary information, Fig. S2l–q). To evaluate the effector function of CD8^+^ T cells, immunostaining for IFNγ and TNFα was performed. The results indicated that Apc knockdown significantly reduced the expression of IFNγ and TNFα in CD8^+^ T cells isolated from MC38 tumors in WT C57BL/6 mice (Supplementary information, Fig. S2r).

These results demonstrate that APC loss induces immune evasion in CRC by impairing CD8^+^ T cell infiltration.

### APC loss confers resistance to PD1 blockade in CRC models

To examine the effect of APC loss on tumor response to immune checkpoint blockade, syngeneic mice were subcutaneously implanted with CT26-WT, CT26-shApc, MC38-WT, or MC38-shApc tumor cells. Mice received anti-PD1 treatment at a suboptimal dose of 100 μg, administered intraperitoneally every three days (Fig. 1o). This regimen significantly inhibited the growth of CT26-WT tumors, but CT26-shApc tumors demonstrated resistance. Similarly, whereas MC38-WT tumors showed a robust response to anti-PD1 therapy, MC38-shApc tumors exhibited a markedly reduced response (Fig. 1o, p; Supplementary information, Fig. S2s). We next analyzed data from the MSKCC cohort,^25^ comprising 99 CRC patients treated with anti-PD1/PD-L1 therapy. Our analysis revealed that patients with APC WT tumors exhibited significantly better overall survival (OS) than those with APC-mutant tumors (Fig. 1q). These results indicate that APC loss promotes resistance to immune checkpoint blockade in CRC models.

### APC loss drives immune evasion in CRC via PTPN13

**Fig. 2:**
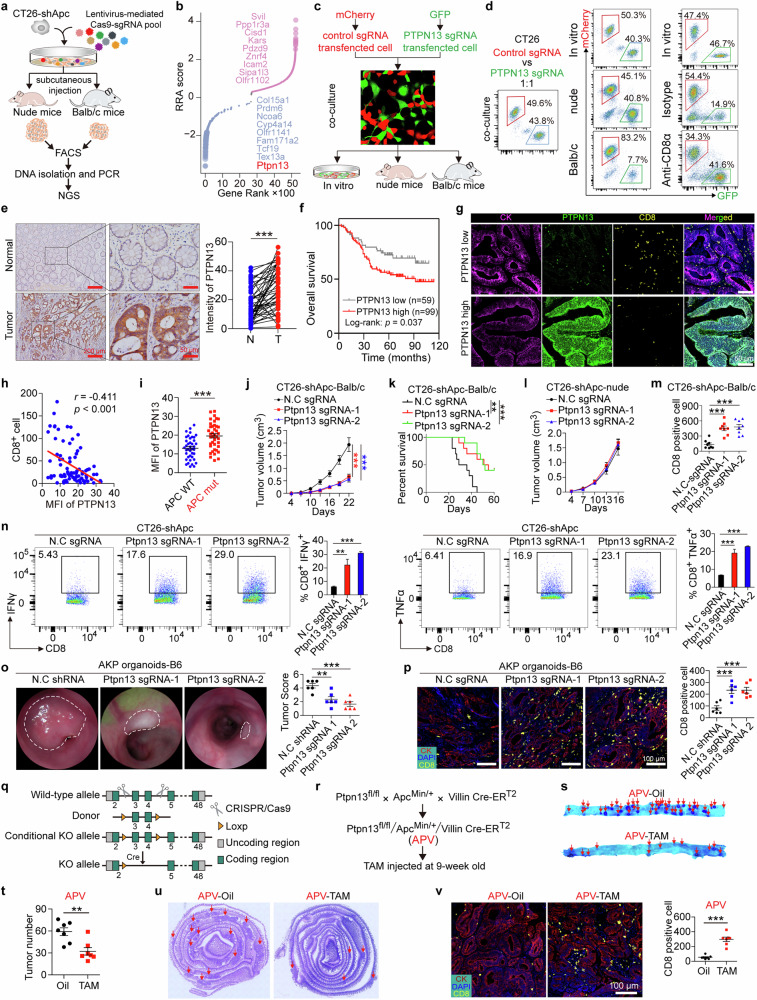
APC loss drives immune evasion in CRC via PTPN13. **a** Schematic illustration of the CT26-shApc in vivo CRISPR screening system. **b** Rank-ordered normalized robust rank aggregation (RRA) scores for genes negatively or positively enriched in WT Balb/c in the CT26-shApc in vivo CRISPR screen. Genes are highlighted in blue (immune-resistor genes) and red (immune-sensitizer genes). The top ten genes are indicated. Dot size is inversely scaled by FDR. FDR < 5%. **c, d** Ptpn13 knockout-GFP and control-mCherry CT26 cells were mixed in vitro and subcutaneously injected at a 1:1 ratio into WT Balb/c and nude mice or WT Balb/c mice pre-injected with anti-CD8α depletion antibody. The GFP/mCherry ratio in vitro and in vivo was determined by FACS. **e** Representative images and quantification of immunohistochemical staining against PTPN13 in paired CRC normal and tumor tissues. *n* = 50, paired *t*-test. **f** Survival analysis of PTPN13-high and PTPN13-low groups from the CRC patient cohort. Log-rank test. **g** Representative immunofluorescence staining of CK (purple), PTPN13 (green), and CD8 (yellow) in CRC primary tissues. **h** Scatterplot showing correlation between MFI of HLA-ABC and CD8^+^ cells. *n =* 80, Pearson’s *r*. **i** MFI of PTPN13 in APC WT and APC-mutated CRC primary tissues. *n* = 80 for each group, unpaired *t*-test. **j** The indicated cells were injected subcutaneously into Balb/c mice, and tumor growth was monitored. *n* = 8 per group, two-way ANOVA. **k** Ptpn13 was knocked out in Apc-silenced CT26 cells, and the cells were then intraperitoneally injected into Balb/c mice. The log-rank test was used to compare survival times. **l** The indicated CT26 cells were transplanted into Balb/c mice, and the tumor growth curve and weight were measured. *n* = 8 per group, two-way ANOVA. **m** Immmunofluorescent staining against CD8 in subcutaneous tumors of Balb/c mice. *n* = 8 per group, one-way ANOVA. **n** Numbers of IFN-γ^+^ or TNF-α^+^ CD8^+^ T cells in tumor-infiltrating lymphocytes (TILs) of Ptpn13-knockout or negative control sgRNA-transfected CT26-shAPC subcutaneous tumors were measured by flow cytometry. *n* = 3 per group, one-way ANOVA. **o** Optical colonoscopy and tumor scoring of orthotopic tumors of Ptpn13-knockout or negative control sgRNA-transfected AKP intestinal organoids injected into WT C57/B6 mice. *n* = 6 per group, unpaired *t*-test. **p** Representative immunofluorescence staining of CK (red) and CD8 (yellow) in tumor tissues and scatterplots showing numbers of CD8^+^ cells in three groups. *n* = 6 per group, one-way ANOVA. **q** Schematic illustration of the CRISPR/Cas9-based Ptpn13 conditional knockout system. Offspring of Ptpn13 conditional knockout mice were screened using PCR, and knockout efficiency was determined by immunoblotting. **r** *Ptpn13*^*fl/fl*^ mice were crossed with *Apc*^*Min/+*^ and *Villin-*CreER^T2^ mice (APV), and tamoxifen (TAM) or oil was injected intraperitoneally in APV mice aged 9 weeks. **s** Representative image of intestines of APV mice treated with TAM or oil. The entire small intestine was systematically divided into three segments, and one representative segment is shown. Red arrows, tumors stained with methylene blue. **t** Scatterplot showing the total number of intestinal tumors across the entire small intestine in APV mice treated with TAM or oil. *n* = 7, unpaired *t*-test. **u** Representative hematoxylin and eosin staining of intestines of APV mice treated with TAM or oil. The entire small intestine was systematically divided into three segments, and one representative segment is shown. Red arrows indicate tumors. **v** Representative immunofluorescence staining of CK (red) and CD8 (yellow) in tumor tissues. Scatterplots show numbers of CD8 positive cells in the tumor tissues of APV mice treated with TAM or oil. *n* = 7, unpaired *t*-test. Data are representative of three independent experiments. All data are mean ± SEM, *P < 0.05, **P < 0.01, ***P < 0.001.

To investigate the mechanism by which APC influences immune evasion in CRC, we performed CRISPR screening to identify potential key regulators. Cas9-expressing CT26-shApc cells, infected with a lentivirus-mediated sgRNA library, were subcutaneously implanted into WT Balb/c and nude mice. Tumors were harvested and sequenced to analyze sgRNA abundance (Fig. 2a; Supplementary information, Table S3). Notably, sgRNAs targeting Ptpn13 were significantly depleted in tumors from WT Balb/c mice compared with those from nude mice, indicating that loss of PTPN13 impairs tumor growth specifically in an immune-competent context and suggesting its critical role in facilitating immune evasion (Fig. 2b). Among the top candidate genes identified in the screen, subsequent functional validation studies revealed that only PTPN13 consistently and robustly regulated immune-related phenotypes. It should be noted that the complex in vivo environment resulted in limited recovery of valid sgRNA reads, rendering this screening approach non-comprehensive. Therefore, these results should be interpreted as a preliminary exploratory screen, in which identified genes (including *PTPN13*) represent candidate hits requiring independent functional validation.

To further investigate the immune-dependent effects of PTPN13 depletion, we performed a competitive tumor growth assay. CT26-control-sgRNA cells (mCherry-labeled) and CT26-Ptpn13-sgRNA cells (GFP-labeled) were mixed at a 1:1 ratio and subcutaneously inoculated into immunocompetent WT Balb/c and immunodeficient nude mice. A parallel experiment with reversed fluorescent labels yielded consistent results. After 14 days, tumor composition was analyzed by fluorescence-activated cell sorting (FACS). In nude mice, the proportions of mCherry⁺ and GFP⁺ cells were comparable, indicating no intrinsic growth disadvantage associated with PTPN13 knockout. By contrast, in WT Balb/c mice, CT26-Ptpn13-sgRNA cells (GFP⁺) were significantly depleted, demonstrating that PTPN13 loss sensitizes tumor cells to immune-mediated elimination (Fig. 2c, d; Supplementary information, Fig. S3a–c).

To directly determine whether CD8⁺ T cell activity mediates this selective clearance, we performed an in vivo depletion experiment. Mice were treated with a CD8-depleting antibody prior to inoculation with a co-culture of GFP-labeled PTPN13-knockout and mCherry-labeled control cells. Flow cytometry analysis of harvested tumors showed that CD8⁺ T cell depletion significantly increased the proportion of GFP⁺ PTPN13-knockout cells compared with isotype control-treated mice (Fig. 2d). This result confirms that CD8⁺ T cell-mediated cytotoxicity is responsible for the selective elimination of PTPN13-deficient tumor cells.

In clinical CRC samples, PTPN13 is elevated in CRC tissue compared with normal colorectal tissue (Fig. 2e). CRC patients with high PTPN13 expression were associated with markedly lower survival rates compared with those with low PTPN13 expression (Fig. 2f). In addition, CRC samples with high PTPN13 expression had significantly fewer CD8^+^ T cells within the tumor microenvironment (Fig. 2g). Pearson’s correlation analysis revealed a significant negative correlation between PTPN13 expression and the number of infiltrated CD8^+^ cells in 80 CRC patients (Fig. 2h, *r* = −0.411, *P* < 0.001). Furthermore, APC-mutant CRC samples expressed higher levels of PTPN13 (Fig. 2i).

We next investigated the role of PTPN13 in APC-loss-induced tumor immune evasion. Ptpn13 knockout significantly suppressed the growth of subcutaneously grafted CT26-shApc cells in Balb/c mice (Fig. 2j; Supplementary information, Fig. S3d) and improved mouse survival in an intraperitoneal tumor implantation model (Fig. 2k). However, in immunodeficient nude mice, Ptpn13 knockout had no effect on the growth of CT26-shApc cells (Fig. 2l; Supplementary information, Fig. S3e). Immunohistological analysis of the CT26-shApc tumors from Balb/c mice showed that Ptpn13 knockout in tumor cells significantly increased CD8^+^ T cell infiltration (Fig. 2m; Supplementary information, Fig. S3f) without affecting tumor cell proliferation (Supplementary information, Fig. S3g). Furthermore, Ptpn13 knockdown in tumor cells significantly enhanced the expression of the effector cytokines IFNγ and TNFα in CD8^+^ T cells isolated from CT26-shApc tumors in WT Balbc mice, indicating an increase in CD8^+^ T cell effector function (Fig. 2n).

To further investigate the impact of PTPN13 inactivation, we engineered an inducible Ptpn13 knockout CT26-shApc cell line using U6-sgRNA-EFS-NSp-Flex-spCAS9 and CreER^T2^ lentivirus, enabling Ptpn13 inactivation upon TAM exposure (Supplementary information, Fig. S3h). TAM treatment effectively suppressed PTPN13 expression, inhibited CT26-shApc cell growth in Balb/c mice (Supplementary information, Fig. S3i–k), and increased CD8^+^ T cell infiltration (Supplementary information, Fig. S3l, m). By contrast, PTPN13 overexpression significantly promoted the growth of subcutaneously grafted CT26 cells in Balb/c mice and reduced CD8^+^ T cell infiltration (Supplementary information, Fig. S3n–q).

In orthotopic transplantation models, AKP organoids were implanted into the colonic mucosa of WT C57BL/6 mice, enabling tumors to be monitored and scored (Supplementary information, Fig. S3r). Ptpn13 knockout significantly inhibited the growth of orthotopically grafted AKP organoids in C57BL/6 mice (Fig. 2o) and increased CD8^+^ T cell infiltration (Fig. 2p). In addition, we developed a conditional Ptpn13 knockout mouse model (*Ptpn13*^*fl/fl*^), which we crossed with *Villin-CreER*^*T2*^ mice and *Apc*^*Min/+*^ mice (spontaneous tumor mouse model carrying a single mutant *Apc* allele) to generate *Apc*^*Min/+*^*/Ptpn13*^*fl/fl*^*/Vil-CreER*^*T2*^ (APV) mice (Fig. 2q, r; Supplementary information, Fig. S3s). TAM treatment in these mice specifically abolished Ptpn13 expression in intestinal epithelial cells, significantly reduced the intestinal adenoma burden (Fig. 2s–u), and increased CD8^+^ T cell infiltration (Fig. 2v).

These results demonstrate that APC-loss-driven CRC immune evasion is mediated by PTPN13.

### APC loss impairs IFNγ-STAT1-IRF1 signaling activation and suppresses antigen presentation

**Fig. 3:**
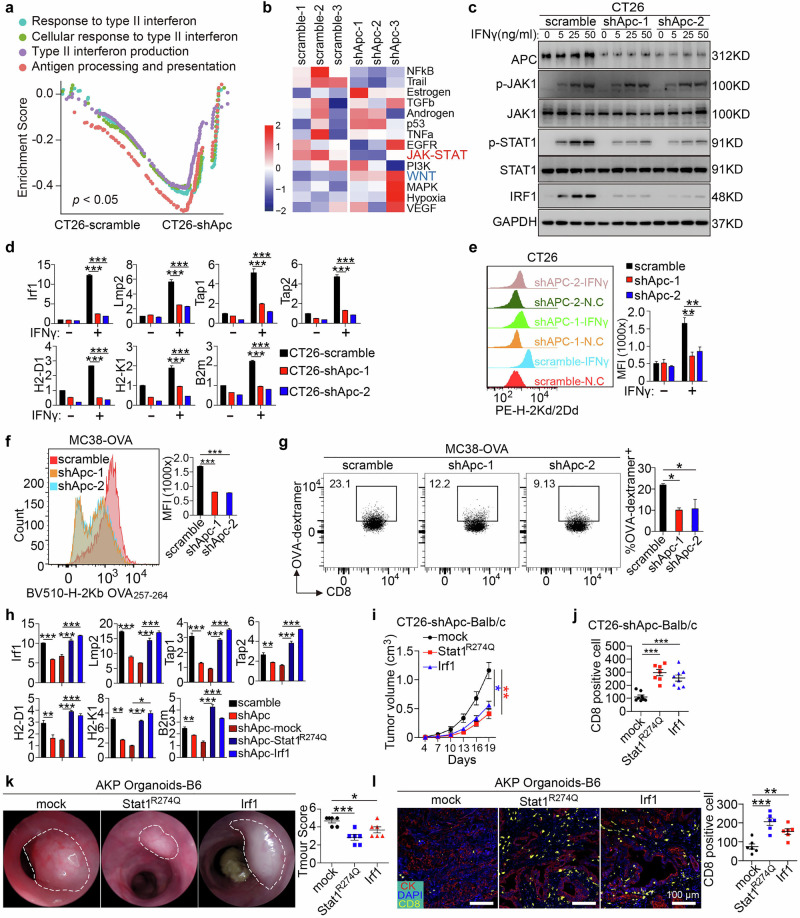
APC loss inactivates IFNγ-STAT1-IRF1-MHC-I antigen presentation signaling. **a** GSEA using GO pathways was performed between shAPC or scramble control transfected CT26 subcutaneous tumors, using gene sets associated with type II interferon response and antigen processing and presentation. **b** Pathway responsive genes for activity inference from gene expression (progeny) analysis performed between tumors formed in Apc-silenced and control groups. **c** Total cell lysates from a series of IFNγ concentrations were subjected to immunoblot analysis with antibodies to the indicated proteins. Data represent three independent experiments. **d** *Irf1, Lmp2, Tap1, Tap2, MHC-I*, and *B2m* mRNA expression (RT-qPCR) in CT26-shApc or CT26-scramble cells. *n* = 6 per group, one-way ANOVA. **e** Flow cytometry histogram and levels of the MHC-I complex on the surfaces of the indicated cells pretreated for 24 h with IFNγ (100 ng/mL) or BSA and stained with anti-H-2Kd/2Dd antibody. Data were calculated from three independent experiments. One-way ANOVA. **f** MC38-OVA-shAPC cells were stimulated with IFNγ (100 ng/mL) or BSA for 24 h, and the numbers of H-2Kb-OVA_257-264_ positive cells and MFI were detected by flow cytometry. Data were calculated from three independent experiments. One-way ANOVA. **g** Numbers of OVA-tetramer positive CD8^+^ T cells in TILs of MC38-OVA-shAPC subcutaneous tumors as detected by flow cytometry. *n* = 3 for each group, one-way ANOVA. **h** *Irf1, Lmp2, Tap1, Tap2, H2-D1, H2K1*, and *B2m* mRNA expression (RT-qPCR) in the indicated cells exposed to IFNγ (50 ng/mL) for 12 h before collection from three independent experiments. One-way ANOVA. **i** APC-silenced CT26 cells were transfected with Stat1^R274Q^ and Irf1 overexpression lentivirus, then subcutaneously xenotransplanted to Balb/c mice; tumor growth was monitored at the indicated times. *n* = 8 for each group, two-way ANOVA. **j** Scatterplot showing numbers of CD8^+^ cells in the indicated groups. *n* = 8, 7, 8 for each group, one-way ANOVA. **k** AKP organoids were transfected with Stat1^R274Q^ and Irf1 overexpression lentivirus, then orthotopically inoculated into C57BL/6 mice; tumor growth was monitored and scored by colonoscopy. *n* = 6 for each group, one-way ANOVA. **l** Representative immunofluorescence staining of CK (red) and CD8 (yellow) in tumor tissues, with scatterplot showing numbers of CD8^+^ cells in three groups. *n* = 6 for each group, one-way ANOVA. Data were calculated from three independent experiments. All data are mean ± SEM, *P < 0.05, **P < 0.01, ***P < 0.001.

To explore the signaling pathway involved in APC-loss-induced immune evasion in CRC, we performed GSEA using GO pathways based on RNA-seq data from CT26-shApc tumors vs control tumors in WT Balb/c mice. The results revealed that APC knockdown was associated with suppression of the response to type II interferon and JAK-STAT signaling pathways (Fig. 3a, b). Analysis of TCGA data further demonstrated inactivation of the response to IFNγ and antigen processing and presentation pathways in APC mutant tumors (Supplementary information, Fig. S4a, b). Specifically, shRNA-mediated Apc knockdown significantly reduced IFNγ-induced STAT1 phosphorylation and IRF1 upregulation, whereas JAK1 phosphorylation remained unaffected, indicating that APC functions downstream of JAK1 and upstream of STAT1 in this pathway (Fig. 3c). Similar results were observed in *Apc*^*fl/fl*^-derived small intestinal crypt cultures following adenovirus-Cre-mediated knockout, as well as in human CRC Ls174.T cells, in which Apc knockout significantly reduced IFNγ-induced STAT1 phosphorylation and IRF1 expression (Supplementary information, Fig. S4c, d).

Using ChIP-grade antibodies specific for phosphorylated STAT1 combined with CUT&Tag sequencing, we identified p-STAT1 binding sites in CT26 cells. p-STAT1 was enriched at promoter regions of key immunoregulatory genes, including those involved in the MHC class I antigen presentation pathway, such as IRF1, LMP2, TAP1, TAP2, H2-D1, H2-K1, and B2M (Supplementary information, Fig. S4e). Overexpression of APC markedly increased the mRNA levels of these genes in CT26 cells under IFNγ stimulation, whereas APC knockout reduced their expression across CT26 cells, *Apc*^*fl/fl*^-Cre intestinal organoids, and Ls174.T cells (Fig. 3d; Supplementary information, Fig. S4f–h). Flow cytometric analysis using anti-H-2Kd/2Dd revealed that Apc knockdown significantly reduced the surface levels of the MHC-I complex on CT26 tumor cells (Fig. 3e).

To further investigate the impact of APC loss on antigen presentation and CD8^+^ T cell function, MC38-OVA cells, which express the ovalbumin (OVA) peptide SIINFEKL, were implanted into WT C57BL/6 mice. Flow cytometry with anti-H-2K^b^ OVA_257-264_ complex antibodies demonstrated a significant reduction in OVA presentation on MC38-Ova cells upon Apc knockdown, indicating impaired antigen presentation (Fig. 3f). Analysis of tumor-infiltrating lymphocytes (TILs) using H-2K^b^-OVA_257-264_ tetramer staining revealed that APC knockdown led to a marked reduction in antigen-specific CD8^+^ T cells (Fig. 3g).

In addition, in MSS CRC patients at comparable disease stages, APC-mutant tumors showed significantly lower HLA-ABC expression and reduced CD8^+^ T cell infiltration compared with APC-WT tumors (Supplementary information, Fig. S4i, j). Pearson’s correlation analysis revealed a significant positive correlation between HLA-ABC expression and the number of infiltrated CD8^+^ cells in 80 patients (*r* = 0.622, *P* < 0.001, Supplementary information, Fig. S4k).

### STAT1/IRF1 activation reverses tumor immune evasion induced by APC loss

To evaluate the role of STAT1/IRF1 in APC-loss-induced immune evasion, we overexpressed a gain-of-function mutant, Stat1^R274Q^, and Irf1 in CT26-shApc cells (Supplementary information, Fig. S4l, m). This intervention significantly upregulated Irf1, Lmp2, Tap1, Tap2, H2-D1, H2-K1, and B2m in CT26-shApc cells (Fig. 3h). In a syngeneic mouse model, overexpression of Stat1^R274Q^ and Irf1 markedly inhibited the growth of subcutaneously grafted CT26-shApc cells in WT Balb/c mice and enhanced CD8^+^ T cell infiltration (Fig. 3i, j; Supplementary information, Fig. S4n, o). However, no such effect was observed in immunodeficient nude mice (Supplementary information, Fig. S4p, q). Furthermore, in orthotopic transplantation models using AKP organoids, overexpression of Stat1^R274Q^ and Irf1 significantly inhibited tumor growth and increased CD8^+^ T cell infiltration (Fig. 3k, l).

These results indicate that APC loss promotes immune evasion by inactivating STAT1/IRF1 signaling, and restoration of this pathway effectively counteracts tumor immune resistance.

### PTPN13 knockout restores APC-loss-induced activation of IFNγ-STAT1-IRF1 signaling

**Fig. 4:**
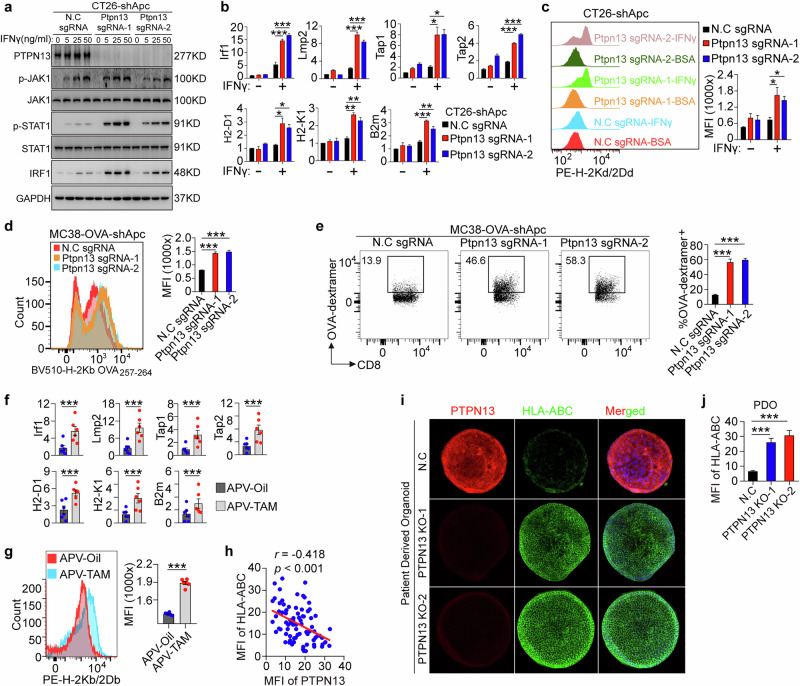
Ptpn13 knockout suppresses APC-loss-induced activation of IFNγ-STAT1-IRF1 signaling. **a** Apc-silenced CT26 cells transfected with negative control (N.C) or two single guide RNAs (sgRNAs) targeting Ptpn13 were stimulated with different concentrations of IFNγ. Total cell lysates were subjected to immunoblot analysis with antibodies to the indicated proteins. Data are representative of three independent experiments. **b** *Irf1, Lmp2, Tap1, Tap2, MHC-I*, and *B2m* mRNA expression was determined by RT-qPCR in Ptpn13-knockout or negative control sgRNA-transfected CT26-shAPC cells with 12 h exposure to IFNγ (50 ng/mL). *n* = 6 per group, one-way ANOVA. **c** Flow cytometry histogram and levels of the MHC-I complex on the surfaces of the indicated cells pretreated for 24 h with IFNγ (100 ng/mL) or BSA and stained with anti-H-2Kd/2Dd antibody. Data were calculated from three independent experiments. One-way ANOVA. **d** Ptpn13-knockout or negative control sgRNA-transfected MC38-OVA-shAPC cells were stimulated with IFNγ (100 ng/mL) or BSA for 24 h, and the numbers of H-2Kb-OVA_257-264_ positive cells and MFI were detected by flow cytometry. Data were calculated from three independent experiments. One-way ANOVA. **e** Numbers of OVA-tetramer positive CD8^+^ T cells in TILs of Ptpn13-knockout or negative control sgRNA-transfected MC38-OVA-shAPC subcutaneous tumors, as detected by flow cytometry. *n* = 3 for each group, one-way ANOVA. **f** *Irf1, Lmp2, Tap1, Tap2, MHC-I*, and *B2m* mRNA expression was determined by RT-qPCR in intestinal tumors of TAM- or oil-treated APV mice. *n* = 6 per group, one-way ANOVA. **g** MFI of H-2Kb/2Db positive cells in intestinal tumors of TAM- or oil-treated APV mice as detected by flow cytometry. *n* = 6 per group, unpaired *t*-test. **h** Scatterplot showing correlation between MFI of HLA-ABC and Ptpn13 IF staining in CRC primary tissues. *n* = 80, Pearson’s *r*. **i, j** CRC-patient-derived organoids (PDO) were cultivated and transfected with Ptpn13-knockout or negative control sgRNA. **i** Representative immunofluorescence staining of Epcam (green), HLA-ABC (red) and CD8 (cyan). **j** MFI of HLA-ABC IF staining in Ptpn13-knockout or negative control sgRNA-transfected PDOs. One-way ANOVA. Data were calculated from three independent experiments. All data are mean ± SEM, *P < 0.05, **P < 0.01, ***P < 0.001.

Notably, Ptpn13 knockout restored STAT1 phosphorylation and IRF1 expression in CT26-shApc cells without affecting JAK1 phosphorylation (Fig. 4a), indicating that APC loss mediates STAT1 dephosphorylation primarily through PTPN13 to dephosphorylate STAT1, rather than altering upstream JAK1 activity. Moreover, Ptpn13 knockout completely reversed the APC-loss-induced reduction in antigen-presentation gene expression and MHC-I surface levels in CT26-shApc cells, *Apc*^*fl/fl*^-Cre intestinal organoids, and Ls174.T-KO APC cells (Fig. 4b, c; Supplementary information, Fig. S4r, s). Ptpn13 knockout also significantly upregulated these genes in both DLD1 (APC-mutant) and LOVO (APC-WT) cells (Supplementary information, Fig. S4t, u).

In the MC38-Ova tumor model, Ptpn13 knockout enhanced OVA antigen presentation on MC38-Ova-shApc cells and increased the frequency of antigen-specific CD8^+^ T cells, indicating improved antigen-specific CD8^+^ T cell responses (Fig. 4d, e).

Analysis of tumors from *Apc*^*Min/+*^*/Ptpn13*^*fl/fl*^*/Vil-CreER*^*T2*^ (APV) mice showed that Ptpn13 knockout, induced by TAM treatment, significantly upregulated the expression of IRF1, LMP2, TAP1, TAP2, H2-D1, H2-K1, and B2M and also increased the surface levels of MHC-I complex on tumor cells (Fig. 4f, g). Furthermore, Pearson’s correlation analysis revealed a significant negative correlation between PTPN13 expression and HLA-ABC expression in 80 CRC patients (Fig. 4h, *r* = −0.418, *P* < 0.001). CRISPR/Cas9-mediated Ptpn13 knockout in patient-derived CRC organoids resulted in a significant increase in HLA-ABC expression (Fig. 4i, j).

These results demonstrate that APC loss downregulates STAT1/IRF1-mediated antigen presentation through PTPN13.

### APC-loss-driven immune evasion in CRC is independent of Wnt/β-catenin pathway activation

Given the role of APC in β-catenin accumulation, we investigated whether β-catenin influences immune evasion mechanisms resulting from APC loss, particularly in relation to T cell infiltration and IFNγ-STAT1-IRF1-MHC class I antigen presentation. shRNA-mediated knockdown of β-catenin in CT26-shApc cells led to a reduction in the expression of β-catenin target genes, including Axin2 and Lgr5 (Supplementary information, Fig. S5a), and inhibited tumor cell proliferation in both WT Balb/c and immunodeficient nude mice without affecting CD8^+^ T cell infiltration (Supplementary information, Fig. S5b–h). Importantly, β-catenin knockdown failed to restore STAT1 phosphorylation or IRF1 expression and did not upregulate IFNγ-induced MHC class I gene expression, further supporting the lack of involvement of β-catenin in immune suppression following APC loss (Supplementary information, Fig. S5i, j).

We also employed an N-terminal (amino acids 1–131) deletion mutant of β-catenin (ΔN-β-catenin), which is resistant to GSK3β phosphorylation.^26^ Overexpression of ΔN-β-catenin resulted in significantly increased expression of Wnt/β-catenin target genes, including Myc, Lgr5, and Axin2, compared with APC knockout. However, unlike APC knockout, ΔN-β-catenin overexpression did not reduce the expression of genes in the IFNγ-induced MHC class I antigen-presentation pathway (Supplementary information, Fig. S5k). In subcutaneously grafted tumor models, ΔN-β-catenin overexpression showed a minimal impact on tumor growth and CD8^+^ T cell infiltration compared with APC knockout, which significantly increased tumor growth and reduced CD8^+^ T cell infiltration (Supplementary information, Fig. S5l–n).

In intestinal organoid cultures, activation of Wnt/β-catenin signaling through R-spondin1, Wnt3a, ΔN-β-catenin, or Apc-sgRNAs led to increased expression of target genes such as Myc, Lgr5, and Axin2. However, IFNγ-induced genes involved in the MHC class I antigen-presentation pathway, including Irf1, Lmp2, Tap1, Tap2, H2-D1, H2-K1, and B2m, were significantly downregulated following Apc-sgRNA treatment, a suppression not observed with R-spondin1, Wnt3a, or ΔN-β-catenin treatments (Supplementary information, Fig. S5o).

Tumors from CT26-shApc vs CT26-shControl, CT26-shApc-shCTNNB1 versus CT26-shApc-shControl, and CT26-shApc-sgPtpn13 versus CT26-shApc-sgControl were collected for RNA-seq. Analysis of biological process GO terms revealed that response to type II interferon and T cell mediated cytotoxicity were significantly downregulated, whereas Wnt signaling pathway was upregulated in APC knockdown CT26 tumors compared with control tumors. Notably, CTNNB1 knockdown downregulated the Wnt signaling pathway but failed to restore the response to type II interferon and T cell mediated cytotoxicity in CT26-shApc tumors. By contrast, Ptpn13 knockdown restored the response to type II interferon and T cell mediated cytotoxicity without affecting the Wnt signaling pathway (Supplementary information, Fig. S6a–c). Heatmap analysis of immune-related genes and Wnt target genes confirmed that APC knockdown significantly downregulated immune-related genes while upregulating Wnt target genes in CT26 tumors. In CT26-shApc tumors, CTNNB1 knockdown downregulated Wnt target genes without affecting immune-related genes, whereas Ptpn13 knockdown upregulated immune-related genes without affecting Wnt target genes (Supplementary information, Fig. S6d–f). These results suggest that APC-loss-induced immune evasion in CRC is dependent on PTPN13 but not on the Wnt/β-catenin pathway.

### Direct interaction and dephosphorylation of STAT1 by PTPN13

**Fig. 5:**
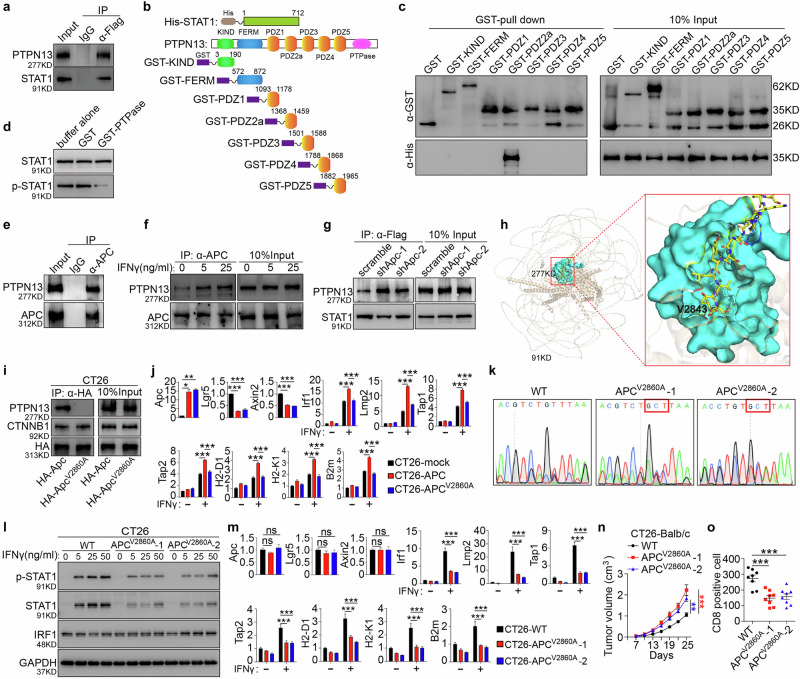
The C-terminal valine of APC is essential for PTPN13 binding and immune evasion in CRC. **a** Flag-tagged Stat1 expression vector (0.5 μg) was transfected into CT26 cells. Total cell lysates were immunoprecipitated with anti-Flag and immunoblotted with anti-PTPN13. **b** Schematic diagram showing the GST-fused PTPN13 motifs and His-tagged STAT1 used in the GST pull-down assays. **c** GST pull-down assays examining the interactions between GST-fused PTPN13 fragments and His-tagged STAT1 protein. Data are representative of three independent experiments. **d** Phosphorylated STAT1 was immunoprecipitated with anti-Flag from IFNγ-stimulated 293T transfectants and incubated with 0.2 mg/mL recombinant GST-PTPase domain of PTPN13. Immunoprecipitates were immunoblotted with anti-phospho-STAT1. Equal loading was verified by reprobing with anti-STAT1. **e** Total cell lysates of CT26 cells were immunoprecipitated with anti-APC and immunoblotted with anti-PTPN13. Data are representative of three independent experiments. **f** CT26 cells were treated with the indicated concentrations of IFNγ, and cell lysates were immunoprecipitated with anti-APC and immunoblotted with anti-PTPN13. **g** A Flag-tagged Stat1 vector (0.5 μg) was transfected into CT26-shApc cells or their control cells. Total cell lysates from the indicated cells were immunoprecipitated with anti-Flag and immunoblotted with anti-PTPN13. Data are representative of three independent experiments. **h** Alphafold3-predicted binding pattern of human APC (brown) and the PDZ2a domain of PTPN13 (cyan). The APC V2843 residue is labeled, and APC Q2829–V2843 residues are shown as sticks and colored in yellow. Hydrogen bonds are shown as yellow dotted lines. **i** CT26 cells were transfected with HA-tagged Apc-WT or Apc^V2860A^ mutant plasmids, and total cell lysates were immunoprecipitated with anti-HA and immunoblotted with anti-PTPN13 and anti-CTNNB1. Data are representative of three independent experiments. **j** IFNγ (50 ng/mL) was administered to CT26 cells transfected with Apc-WT or Apc^V2860A^ mutant plasmids for 12 h, and *Apc, Lgr5, Axin2, Irf1, Lmp2, Tap1, Tap2, H2-D1, H2K1*, and *B2m* mRNA expression was detected by RT-qPCR. Data are representative of three independent experiments. One-way ANOVA. **k** CRISPR/Cas9-based establishment of APC^V2860A^ point mutation. **l** CT26 cells transfected with APC-WT or APC^V2860A^ mutant plasmids (CT26-APC^V2860A^-1/2) were incubated with or without IFNγ at the indicated concentrations for 2 h, and total cell lysates were subjected to immunoblot analysis. **m** IFNγ (50 ng/mL) was administered to CT26 cells transfected with Apc-WT or Apc^V2860A^ mutant plasmids (CT26-APC^V2860A^-1/2) for 12 h, and *Apc, Lgr5, Axin2, Irf1, Lmp2, Tap1, Tap2, H2-D1, H2K1*, and *B2m* mRNA expression was detected by RT-qPCR. Data are representative of three independent experiments. One-way ANOVA. **n** CT26 cells harboring gRNA-induced mutant APC^V2860A^ (CT26-APC^V2860A^-1/2) and the WT control were subcutaneously injected (2 × 10^6^ cells) into Balb/c mice, and tumor growth was monitored. *n* = 8 for each group, two-way ANOVA. **o** Immunofluorescence of CD8^+^ cell infiltration in subcutaneous tumors of CT26-APC^V2860A^ and the control group. *n* = 8 for each group, one-way ANOVA. Data are representative of three independent experiments. All data are mean ± SEM, *P < 0.05, **P < 0.01, ***P < 0.001.

Previous studies have implicated PTPN13 in the dephosphorylation of STAT family members.^27–29^ To investigate whether PTPN13 interacts with STAT1, we transfected CT26 and Ls174.T cells with Flag-tagged STAT1 and used co-immunoprecipitation to confirm the STAT1–PTPN13 interaction (Fig. 5a; Supplementary information, Fig. S7a). To identify the specific interaction domain, various truncations of PTPN13 were engineered (Fig. 5b). Using glutathione S-transferase (GST)-tagged PTPN13 truncations and His-tagged STAT1, a GST-Sepharose bead pull-down assay confirmed that STAT1 binds directly to the PDZ2a domain of PTPN13 (Fig. 5c).

To assess whether PTPN13 directly dephosphorylates STAT1, we performed an in vitro PTPase assay with recombinant proteins. The purified GST-tagged PTPase domain of PTPN13 was incubated with immunoprecipitated, tyrosine-phosphorylated STAT1 from IFNγ-stimulated, Flag-tagged STAT1-transfected 293T cells, resulting in STAT1 dephosphorylation (Fig. 5d). These results demonstrate that STAT1 is a direct substrate of PTPN13.

### APC blocks the STAT1–PTPN13 interaction

To better understand how APC regulates PTPN13/STAT1 signaling, we examined the APC–PTPN13 interaction. Prior studies suggested that the C terminus of APC binds the PDZ2a domain of PTPN13,^30^ but its physiological presence was uncertain. Co-immunoprecipitation in CT26 and Ls174.T cells confirmed an endogenous APC–PTPN13 interaction (Fig. 5e; Supplementary information, Fig. S7b), and IFNγ stimulation enhanced the formation of this complex (Fig. 5f). We hypothesized that APC blocks the STAT1–PTPN13 interaction, and this notion was supported by the enhanced PTPN13–STAT1 interaction in Apc knockdown cells (Fig. 5g). Although PTPN13 was found to interact with β-catenin, it did not bind to other core components of the canonical destruction complex, such as GSK3β or YAP1 (Supplementary information, Fig. S7c). These results suggest that the APC–PTPN13–STAT1 axis does not integrate into the classical β-catenin destruction complex but may instead function as a distinct regulatory complex.

### The C-terminal valine of APC is essential for PTPN13 binding and immune evasion in CRC

We used AlphaFold3 to model the interaction between APC and the PTPN13 PDZ2a segment, revealing that the C-terminal valine of APC is crucial for binding to PTPN13 (Fig. 5h). To confirm the critical role of the terminal valine residue (V2843) in this interaction, we generated full-length APC mutants — V2860A (murine) and V2843A (human) — and transfected them into mouse or human CRC cells. In CT26 cells, a co-immunoprecipitation assay showed that WT APC bound both PTPN13 and β-catenin, whereas the APC^V2860A^ mutant lost its binding affinity for PTPN13 but retained interaction with β-catenin, highlighting the specificity of V2860 for PTPN13 binding (Fig. 5i). Both WT APC and the APC^V2860A^ mutant downregulated Wnt target genes such as Myc and Axin2. However, only WT APC upregulated IFNγ-induced MHC-I pathway genes, with no effects observed in the APC^V2860A^ mutant (Fig. 5j). Similar results were observed in the human CRC cell lines Ls174.T and DLD1 (Supplementary information, Fig. S7d, e).

Using CRISPR/Cas9 technology to introduce a V2860A mutation into CT26 cells, we successfully generated two mutant clones: APC^V2860A^-1 and APC^V2860A^-2 (Fig. 5k). IFNγ-induced STAT1 phosphorylation and IRF1 upregulation were significantly inhibited in these clones (Fig. 5l). The mRNA levels of genes involved in the MHC class I antigen-presentation pathway induced by IFNγ, including IRF1, LMP2, TAP1, TAP2, MHC-I, and B2M, were significantly reduced (Fig. 5m). When these clones were transplanted subcutaneously into WT Balb/c mice, they displayed accelerated tumor growth and reduced CD8^+^ T cell infiltration, indicating impaired immune surveillance due to loss of APC’s C-terminal valine (Fig. 5n, o; Supplementary information, Fig. S7f).

These results demonstrated that the C-terminal valine of APC is crucial for its interaction with PTPN13 and that this interaction is essential for regulating immune evasion in CRC.

### Structural basis for binding of APC11 to the PDZ2a domain of PTPN13

**Fig. 6:**
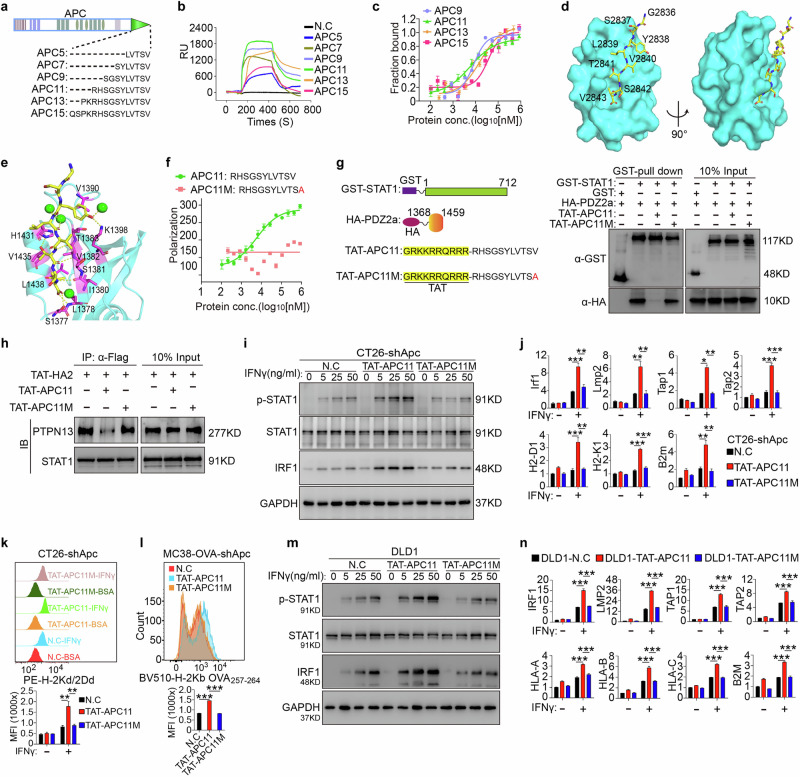
APC11 blocks the PTPN13–STAT1 interaction and restores IFNγ-STAT1-IRF1-MHC-I antigen presentation signaling impaired by APC loss. **a** Schematic diagram showing the indicated residues at the APC C terminus. **b** Kinetics of the interaction between PDZ-2a and the indicated residues of APC were explored by surface plasmon resonance-based binding assays. **c** Binding affinities of PDZ2a to APC C-terminal peptides of different lengths, as measured by an FP assay. **d** The 2.1-Å complex structure of the PDZ2a domain (1364–1446 aa) and the APC11 peptide. PDZ2a is shown in cyan and presented as a surface diagram, whereas the peptide is shown in yellow and presented as a stick diagram. **e** Detailed interactions between the APC11 peptide and PDZ2a within the complex. The PDZ2a residues involved are labeled and shown as magenta sticks, and the peptide-interacting water molecules are shown as green balls. Hydrogen bonds are shown as yellow dotted lines. **f** Binding affinity of PDZ2a to the WT APC11 peptide and the APC11M mutant (V2843A) as measured by an FP assay. **g** GST-fused STAT1 was incubated with HA-tagged PDZ2a and with TAT-APC11 or TAT-APC11M peptides, immunoprecipitated with GST beads, and immunoblotted with anti-GST and anti-HA antibodies. Data are representative of three independent experiments. **h** CT26 cells transfected with a Flag-tagged Stat1 vector were incubated with 25 μM TAT-HA2, with or without 50 μM TAT-APC11 or TAT-APC11M, for 4 h. Total cell lysates were immunoprecipitated with anti-Flag and immunoblotted with anti-PTPN13. Data are representative of three independent experiments. **i** Apc-silenced CT26 cells were incubated with 25 μM TAT-HA2, with or without 50 μM TAT-APC11 or TAT-APC11M, for 2 h and then treated with or without IFNγ at the indicated concentrations for 2 h. Total cell lysates were subjected to immunoblot analysis. Data are representative of three independent experiments. **j** *Irf1, Lmp2, Tap1, Tap2, H2-D1, H2K1*, and *B2m* mRNA expression (RT-qPCR) in Apc-silenced CT26 cells exposed to IFNγ (50 ng/mL) for 12 h before collection from three independent experiments. One-way ANOVA. **k** Apc-silenced CT26 cells were incubated with 25 μM TAT-HA2, with or without 50 μM TAT-APC11 or TAT-APC11M, for 2 h and then treated with or without IFNγ (100 ng/mL) for 24 h. FACS histogram and quantification of the MHC-I complex on the surfaces of the indicated cells stained with anti-H-2Kd/2Dd antibody or isotype control antibodies. Data were calculated from three independent experiments. One-way ANOVA. **l** Apc-silenced MC38-OVA_257-264_ cells were incubated with 25 μM TAT-HA2, with or without 50 μM TAT-APC11 or TAT-APC11M, for 2 h and then treated with or without IFNγ (100 ng/mL) for 24 h. FACS histogram and quantification of OVA_257-264_-specific MHC-I complex on the surfaces of the indicated cells stained with anti-H-2Kb/SIINFEKL antibody or isotype control antibodies. Data represent three independent experiments. One-way ANOVA. **m** DLD1 cells were incubated with 25 μM TAT-HA2, with or without 50 μM TAT-APC11 or TAT-APC11M, for 2 h and then treated with or without IFNγ at the indicated concentrations for 2 h. Total cell lysates were subjected to immunoblot analysis. Data are representative of three independent experiments. **n** *IRF1, LMP2, TAP1, TAP2, HLA-A, HLA-B, HLA-C*, and *B2M* mRNA expression (RT-qPCR) in the indicated cells after exposure to IFNγ (50 ng/mL) for 12 h before collection from three independent experiments. One-way ANOVA. All data are mean ± SEM, *P < 0.05, **P < 0.01, ***P < 0.001.

To examine the interaction between APC and PTPN13 in detail, we synthesized a series of peptides from the C terminus of APC and measured their binding affinities to the PDZ2a domain of PTPN13 using surface plasmon resonance (SPR). Among the tested peptides, APC11, an 11-amino-acid sequence, exhibited the highest affinity for PDZ2a, with a dissociation constant (*K*_d_) of ~6 µM, as confirmed by fluorescence polarization (FP) assays. By contrast, longer APC peptides showed progressively weaker binding affinities (Fig. 6a–c).

To test the function of APC C-terminal peptides of various lengths, each peptide was conjugated at the N terminus with the cell-penetrating peptide “TAT” (sequence: GRKKRRQRRR).^31^ These TAT-conjugated peptides were incubated with CT26-shApc cells. All APC-peptides significantly increased the expression of genes involved in the MHC class I antigen-presentation pathway induced by IFNγ, including Irf1, Lmp2, Tap1, Tap2, H2-D1, H2-K1, and B2m in CT26-shApc cells. TAT-APC11 yielded the most substantial increase (Supplementary information, Fig. S8a). TAT-APC11 also showed the most effective tumor suppression (Supplementary information, Fig. S8b, c) and augmentation of intratumoral CD8^+^ T cell infiltration (Supplementary information, Fig. S8d, e) in a syngeneic mouse colon cancer model. Subsequent experiments therefore focused on the APC11 peptide.

To understand the structural basis of this interaction, we crystallized the PDZ2a domain (1364–1446 aa) of PTPN13 bound to APC11 and solved the structure at a resolution of 2.1 Å in a C121 space group (Fig. 6d; Supplementary information, Table S2). Although PDZ2a acted as a monomer in solution, the crystal structure revealed a dimeric arrangement due to crystal packing. The two PDZ2a protomers shared a high degree of structural similarity (RMSD of 1.47 Å for all Cα atoms), with the main structural variation located at the α1 helix (Supplementary information, Fig. S8f, g). Comparison of the APC11-bound PDZ2a structure with the apo PDZ2 structure (PDB ID: 3LNX)^32^ revealed that the binding of APC11 did not induce substantial conformational changes, supporting a “lock-and-key” binding model (Supplementary information, Fig. S8h, i). Within the complex, only the last six residues of APC11 (Y2838 to V2843) interact directly with PDZ2a, forming hydrogen bonds and engaging in hydrophobic interactions that secure the peptide within the groove between the β2 strand and α2 helix of PDZ2a (Fig. 6e). The C-terminal residue, V2843, plays a crucial role in anchoring APC11 through hydrogen bonding and hydrophobic interactions, fitting into a binding pocket lined by L1378, I1380, V1382, V1435, and L1438 on PDZ2a (Fig. 6d, e).

We generated an APC11 peptide point mutant (V2843A, hereafter APC11M) and reassessed its binding affinity using an FP assay. Results confirmed that the binding of APC11M to PDZ2a was severely impaired (Fig. 6f), consistent with our structural hypothesis that the shorter side chain of alanine may not fully occupy the hydrophobic pocket in PDZ2a, thus weakening the interaction.

### APC11 blocks the PTPN13–STAT1 interaction and restores the IFNγ-STAT1-IRF1-MHC-I antigen presentation signaling impaired by APC loss

We next tested the capacity of APC11 to function as an inhibitor of the PTPN13–STAT1 interaction. To facilitate cellular entry, APC11 peptides were conjugated at the N terminus with the cell-penetrating peptide “TAT”. The TAT-conjugated APC11 peptide (TAT-APC11), but not the mutant TAT-APC11M, effectively disrupted the interaction between STAT1 and the PDZ2a domain of PTPN13, as shown in a GST-Sepharose bead pulldown assay (Fig. 6g). In addition, co-immunoprecipitation in CT26 cells expressing Flag-tagged STAT1 showed that TAT-APC11 significantly inhibited the PTPN13–STAT1 interaction, whereas TAT-APC11M did not (Fig. 6h).

In CT26-shApc cells, treatment with TAT-APC11, but not TAT-APC11M, enhanced STAT1 phosphorylation and upregulated IRF1 expression (Fig. 6i). TAT-APC11, but not the mutant TAT-APC11M, significantly upregulated genes involved in the IFNγ-induced MHC class I antigen-presentation pathway, including Irf1, Lmp2, Tap1, Tap2, H2-D1, H2-K1, and B2m (Fig. 6j). Similar results were observed in MC38-shApc cells and *Apc*^*fl/fl*^*-Cre* organoid cultures (Supplementary information, Fig. S8j, k). Flow cytometry confirmed that TAT-APC11, but not TAT-APC11M, increased MHC-I complex levels on the cell surface (Fig. 6k). Treatment with TAT-APC11, but not TAT-APC11M, markedly increased the surface expression of the Ova antigen on MC38-Ova tumor cells, suggesting improved antigen presentation (Fig. 6l). In the human CRC cell line DLD1, TAT-APC11 selectively increased STAT1 phosphorylation and expression of IRF1 and MHC-I components (HLA-A, HLA-B, HLA-C, and B2M), whereas TAT-APC11M exhibited no effect (Fig. 6m, n). However, neither APC11 nor APC11M triggered apoptosis or influenced the expression and activation of caspase-3 and caspase-8 in CT26-shApc cells (Supplementary information, Fig. S8l–n).

These results demonstrate that APC11 effectively blocks the PTPN13/STAT1 interaction, thereby restoring APC-loss-induced inactivation of the IFNγ-STAT1-IRF1-MHC-I antigen-presentation pathway without inducing apoptosis.

### APC11 inhibits tumor growth and enhances the response to anti-PD1 therapy

**Fig. 7:**
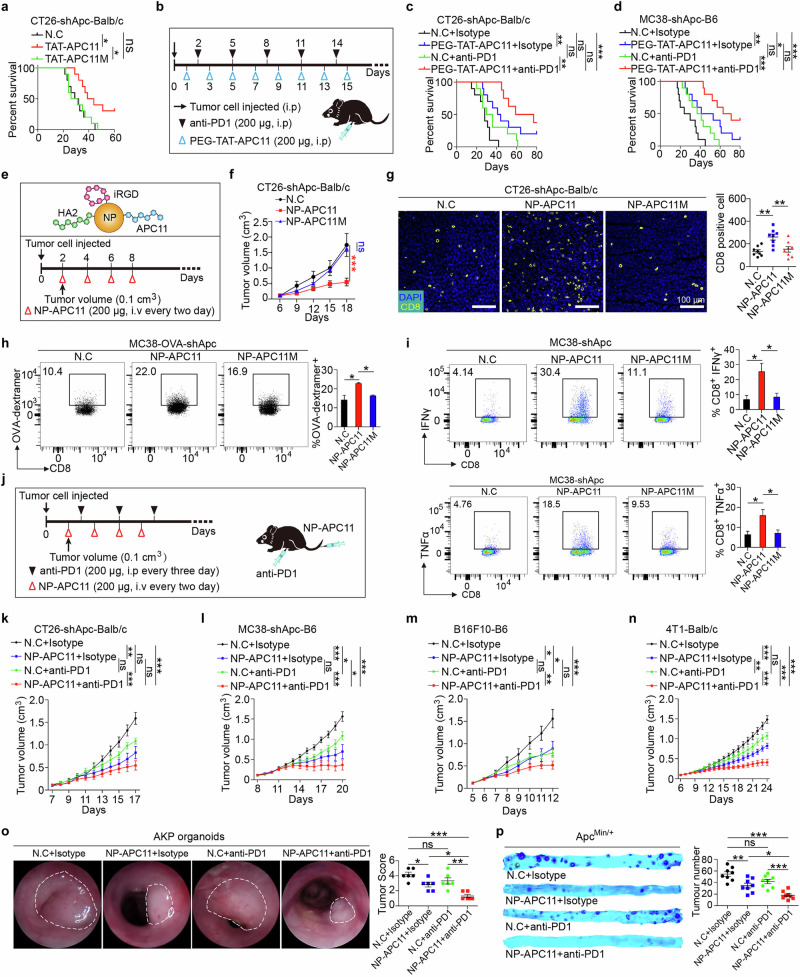
APC11 inhibits tumor growth and enhances the response to anti-PD1 therapy. **a** CT26-shApc cells were intraperitoneally injected into mice, followed by intraperitoneal administration of PEG-conjugated TAT-APC11 peptides, and survival percentages were compared. *n* = 10 per group, log-rank test. **b** Schematic showing the combination treatment of anti-PD1 antibody and PEG-coupled TAT-APC11 peptide at the indicated times. **c, d** The indicated cells were intraperitoneally injected into mice, followed by intraperitoneal administration of anti-PD1 antibodies and PEG-conjugated TAT-APC11 peptides, and survival percentages were compared. *n* = 10 per group, log-rank test. **e** Schematic of the combination treatment of anti-PD1 antibodies and nanoparticle-coupled APC11 peptide (NP-APC11). **f** Apc-silenced CT26 cells were injected subcutaneously (1 × 10^7^ cells) into Balb/c mice. Subsequently, 200 μg NP-APC11 or NP-APC11M was injected intraperitoneally every other day at the indicated times when the tumor reached 500 mm^3^. Tumor growth was measured. *n* = 8 per group, two-way ANOVA. **g** Immunofluorescence of CD8^+^ cell infiltration into NP-APC11- or NP-APC11M-treated CT26-shAPC subcutaneous tumors and the control group (DAPI, nucleus). *n* = 8 for each group, one-way ANOVA. **h** Apc-silenced MC38-OVA_257-264_ cells were injected subcutaneously (1 × 10^7^ cells) into C57BL/6 mice. Subsequently, 200 μg NP-APC11 or NP-APC11M was injected intraperitoneally every other day three times when the tumor reached 100 mm^3^. FACS histogram and quantification of OVA_257-264_-tetramer on the surfaces of tumor-infiltrating CD8^+^ T cells. *n* = 3, one-way ANOVA. **i** Apc-silenced MC38-OVA_257-264_ cells were injected subcutaneously (1 × 10^7^ cells) into C57BL/6 mice. Subsequently, 200 μg NP-APC11 or NP-APC11M was injected intraperitoneally every other day when the tumor reached 100 mm^3^. FACS histogram and quantification of IFNγ^+^ or TNFα^+^ in tumor-infiltrating CD8^+^ T cells. *n* = 3, one-way ANOVA. **j** Schematic of the combination treatment of anti-PD1 antibody and nanoparticle-conjugated APC11 peptide at the indicated times. **k–n** The indicated cells were injected subcutaneously (1 × 10^7^ cells) into mice, anti-PD1 antibodies were injected intraperitoneally, and NP-APC11 was injected via the caudal vein. Tumor growth was monitored, and tumor weight was measured. *n* = 8 for each group, two-way ANOVA for tumor volume, one-way ANOVA for tumor weight. **o** AKP organoids were orthotopically inoculated into C57BL/6 mice, anti-PD1 antibodies were injected intraperitoneally, and NP-APC11 was injected via the caudal vein. Tumor growth was monitored and scored by colonoscopy. *n* = 6, one-way ANOVA. **p** Representative methylene blue-stained images of the intestines of *Apc*^*Min/+*^ mice treated with anti-PD1 antibodies and/or NP-APC11. Scatterplot shows the numbers of intestinal tumors in *Apc*^*Min/+*^ mice treated with anti-PD1 antibodies and/or NP-APC11. *n* = 8, one-way ANOVA. All data are mean ± SEM, *P < 0.05, **P < 0.01, ***P < 0.001.

In an intraperitoneal tumor model using Balb/c mice injected with CT26-shApc cells, treatment with TAT-APC11, but not the mutant form TAT-APC11M, significantly improved survival (Fig. 7a). To enhance solubility and stability, TAT-APC11 was conjugated with polyethylene glycol (PEG).^33^ We then evaluated the therapeutic potential of PEG-TAT-APC11 both as a standalone treatment and in combination with immune checkpoint blockade. In this model, anti-PD1 monotherapy did not improve survival, whereas PEG-TAT-APC11 extended it. The combination of PEG-TAT-APC11 and anti-PD1 further improved survival outcomes (Fig. 7b, c). Similar results were obtained in C57BL/6 mice injected with MC38-shApc cells: both PEG-TAT-APC11 and anti-PD1 monotherapies extended survival, with the combination therapy being most effective (Fig. 7d).

To further optimize tumor targeting, APC11-based nanoparticles (NP-APC11) were developed and conjugated with the tumor-penetrating peptide iRGD^34^ to improve selectivity. Pharmacokinetic analysis showed low serum levels of both APC11 and NP-APC11 at 2 h and 4 h post administration, with peak concentrations detected in urine at 48 h and 24 h, respectively. Both compounds preferentially accumulated in liver and kidney tissues, with minimal distribution to other organs such as the heart, lungs, spleen, brain, and intestines. Notably, significant enrichment of APC11 and NP-APC11 was observed in tumor tissues, underscoring their efficacy in targeting cancer cells (Supplementary information, Fig. S9a–f).

Following tumor establishment (~100 mm³) in subcutaneously grafted CT26-shApc tumor models, mice were treated with NP-APC11 or the mutant form NP-APC11M via intravenous injection (Fig. 7e). NP-APC11, but not NP-APC11M, significantly suppressed tumor growth and increased CD8^+^ T cell infiltration (Fig. 7f, g; Supplementary information, Fig. S9g). RNA sequencing of tumors treated with APC11 peptide confirmed significant upregulation of gene signatures related to CD8⁺ T cell infiltration and activation. Transcriptomic analysis further revealed broad restoration of multiple immune-related pathways beyond the STAT1–IRF1 axis (Supplementary information, Fig. S9h, i).

NP-APC11 also demonstrated therapeutic efficacy in larger tumors (500 mm³), further emphasizing its potential as an effective treatment (Supplementary information, Fig. S9j, k). In the MC38-Ova tumor model, NP-APC11, but not NP-APC11M, markedly increased the number of H-2K^b^-OVA_257-264_ tetramer^+^ CD8^+^ tumor-infiltrating lymphocytes, indicating enhanced antigen-specific T cell infiltration (Fig. 7h). In addition, NP-APC11 treatment, in contrast to NP-APC11M treatment, significantly increased the expression of the effector cytokines IFNγ and TNFα in CD8^+^ T cells, highlighting its role in enhancing the anti-tumor immune response (Fig. 7i).

We further evaluated the efficacy of combining NP-APC11 with anti-PD1 in tumor treatment. Syngeneic mice were subcutaneously grafted with CT26-shApc, MC38-shApc, B16F10, or 4T1 tumor cells. Once the tumors reached 100 mm³, NP-APC11 was administered via the caudal vein, and anti-PD1 antibodies were given intraperitoneally (Fig. 7j). In all four tumor models, NP-APC11 significantly inhibited tumor growth. The combination of NP-APC11 with anti-PD1 had a more potent therapeutic effect than anti-PD1 monotherapy (Fig. 7k–n; Supplementary information, Fig. S9l–o).

In orthotopic transplantation models, NP-APC11, but not anti-PD1, effectively reduced tumor growth in CT26-shApc and AKP organoid models, with combination therapy yielding the most potent effects (Fig. 7o; Supplementary information, Fig. S9p). In the *Apc*^*Min/+*^ spontaneous tumor mouse model, NP-APC11 significantly reduced adenoma formation, outperforming anti-PD1 alone, and the combination therapy resulted in further tumor suppression (Fig. 7p).

These results demonstrate the potential of APC11, either alone or in combination with anti-PD1, as an effective therapeutic strategy for treating APC-deficient CRC, with possible applications in other tumor types.

## Discussion

The identification of a previously unrecognized APC/PTPN13/STAT1-dependent tumor immune-suppressive mechanism in this study represents a significant advance, offering a compelling strategy for development of therapeutic interventions for CRC. On the basis of this mechanism, the APC11 peptide has been designed to disrupt the PTPN13–STAT1 interaction, thereby inhibiting tumor immune evasion. Multiple formulations of this peptide, including TAT-APC11, PEG-TAT-APC11, and NP-APC11, exhibited significant anti-tumor efficacy. Furthermore, the enhanced tumor-suppressing effects observed when APC11 peptides were combined with anti-PD1 antibodies highlight a promising therapeutic strategy, providing a strong rationale for further development of anti-tumor agents and clinical trials for CRC patients.

Despite recent advances in cancer research and treatment, CRC remains among the deadliest malignancies worldwide.^1,2^ Notably, more than 80% of CRC cases harbor loss-of-function mutations in the Apc gene,^14–16^ emphasizing the critical need to understand the function of APC in CRC pathogenesis in order to develop more effective therapies for this patient population. APC is a large protein (310 kDa, 2843 amino acids) that plays an essential role in the β-catenin destruction complex, promoting β-catenin degradation via the proteasome and thereby inhibiting Wnt/β-catenin signaling to regulate tumor cell proliferation and differentiation.^17–19^ The 15- and 20-residue repeat domains and SAMP repeats (1000 to 2100 aa), are essential for the interactions of APC with β-catenin and Axin, respectively.^35^ The C-terminal region of APC has non-Wnt signaling functions, including interactions with microtubules, end-binding protein 1 (EB1), and discs large 1 (DLG1), which are important for cytoskeletal dynamics, cell polarity, and migration.^36^ APC has also been shown to influence T cell function by regulating the nuclear localization of NFAT, which is required for cytokine gene transcription and immune regulation.^37^ Our study reveals that the C terminus of APC interacts directly with the PDZ2a domain of PTPN13, inhibiting the interaction between PTPN13 and STAT1. This enhances STAT1 phosphorylation and activates the IFNγ-STAT1-IRF1-MHC class I antigen-presentation pathway, which ultimately leads to increased CD8^+^ T cell infiltration and suppression of tumor growth, independently of β-catenin (summarized in Supplementary information, Fig. S10a, b).

Specifically, the C-terminal valine residue of APC is essential for its binding to PTPN13. Mutation of this residue (V2860A in murine APC or V2843A in human APC) significantly impaired the interaction between APC and PTPN13, leading to reduced activation of IFNγ-STAT1 signaling, lower immune cell infiltration, and greater tumor growth, without affecting the interaction between APC and β-catenin. We generated a mouse model in which an HA tag was fused to the C terminus of APC, thereby disrupting the interaction with PTPN13, and this also resulted in increased tumor burden and reduced immune activity. Consistent with our findings, Yoshimi et al. created a Kyoto Apc Delta (KAD) rat model harboring a nonsense mutation in the *Apc* gene (S2523X) that truncated the C-terminal region but retained intact β-catenin-binding domains. This model exhibited a significant increase in colon tumor incidence, underscoring the crucial role of the APC C terminus in tumorigenesis.^38^ The majority of APC mutations (~90%) result in premature stop codons, leading to truncated proteins that lack both the C-terminal PTPN13-binding domain and the regions responsible for β-catenin regulation.^35,39,40^ As a result, APC mutations simultaneously activate Wnt/β-catenin signaling, promoting tumor proliferation, while also impairing the IFNγ-STAT1-IRF1-MHC class I antigen presentation pathway, promoting immune evasion. This mechanism provides insight into how APC truncations — frequently observed in CRC — contribute to tumor immune evasion, emphasizing the potential of targeting this specific interaction as a therapeutic strategy (summarized in Supplementary information, Fig. S10c).

The APC11 peptide, derived from the C-terminal sequence of APC, effectively disrupts the PTPN13–STAT1 interaction, thereby enhancing STAT1 phosphorylation and reactivating the IFNγ-STAT1-IRF1-MHC class I antigen-presentation pathway. This leads to increased CD8^+^ T cell infiltration and tumor suppression, as demonstrated in murine CRC models (summarized in Supplementary information, Fig. S10d). Unlike previously reported strategies that directly target oncogenic signaling within APC-truncated cells — such as inhibiting cholesterol biosynthesis (TASIN-1)^21^ or disrupting cholesterol-mediated Wnt activation through Dvl interaction²² — our study identified an immune-suppressive mechanism driven by APC and demonstrated that the APC11 peptide can restore anti-tumor immunity by blocking the PTPN13–STAT1 interaction. This suggests that combining APC11 with agents such as TASIN-1 or Dvl–cholesterol inhibitors may provide a synergistic therapeutic strategy that simultaneously targets both oncogenic signaling and immune escape in APC-mutant CRC.

Although the MC38 model used here is highly immunogenic and may not fully represent non-inflamed human CRCs, the efficacy of the APC11 peptide was also confirmed in two genetically engineered mouse models of APC loss — orthotopically transplanted AKP organoids (carrying Apc/Kras/p53 mutations) and *Apc*^*Min/+*^ spontaneous intestinal tumors. In both models, APC11 significantly suppressed tumor growth and synergized with anti-PD1 therapy, demonstrating its therapeutic potential even in immune-resistant and non-inflamed microenvironments. Further studies using additional models that more closely mimic clinical disease settings are warranted to fully evaluate the clinical translational potential of the APC11 peptide.

Importantly, TAT-APC11 and PEG-TAT-APC11 significantly improved survival in a murine model of intraperitoneal tumor implantation, thus addressing peritoneal metastasis — a major cause of mortality in CRC patients.^41^ Consequently, APC11-based therapies represent a promising avenue for treating CRC with peritoneal metastasis and warrant expedited clinical development. Furthermore, the combination of anti-PD1 antibodies with PEG-TAT-APC11 or NP-APC11 demonstrated synergistic anti-tumor effects in APC-mutant CRC, which is typically resistant to anti-PD1 therapy, as well as in melanoma and breast cancer models. Notably, APC11 not only inhibited the growth of APC mutant tumors but also suppressed the growth of APC WT tumors. This is because the APC11 peptide mimics the effect of APC overexpression, which can further enhance the inhibition of PTPN13 and the activation of IFNγ-STAT1 signaling in APC intact tumor cells, thereby enhancing the immune response. These findings support the broad therapeutic potential of APC11 in both APC mutant and APC WT tumors and its ability, in combination with immune checkpoint inhibitors, to enhance anti-tumor efficacy across multiple cancer types. Patients with high tumor expression of PTPN13 may particularly benefit from APC11-based therapies.

The co-crystal structure of the PTPN13/APC11 complex reveals the precise mechanism by which APC11 targets PTPN13. The terminal valine of APC11 plays a key role in binding to PTPN13, as mutation of this residue to alanine significantly reduces the binding affinity and anti-tumor efficacy of the peptide. Future research should focus on optimizing APC11 and developing derivative peptides or small-molecule mimetics that target PTPN13 more effectively. Improving peptide stability, targeting precision, and cellular uptake could enhance the clinical applicability of APC11. Strategies such as introduction of functional groups to the N terminus of APC11 or development of PEGylated versions could improve its pharmacokinetic profile. Moreover, by leveraging structural information on the APC11–PTPN13 interaction, a high-throughput screening method could be developed to identify small molecules capable of competitively binding PTPN13. Such small-molecule inhibitors would offer advantages in terms of oral bioavailability and manufacturing scalability and could potentially be used in combination with existing immunotherapies.

Hyperactivation of Wnt signaling has been linked to immune suppression through various mechanisms, including TCF/β-catenin-mediated transcriptional activation of PD-L1, which contributes to tumor immune evasion.^42,43^ However, our study suggests that APC-loss-induced immune evasion in CRC is dependent on PTPN13 but not on the Wnt/β-catenin pathway. APC loss led to suppression of STAT1/MHC-I signaling, which could be reversed by Ptpn13 knockdown but not by CTNNB1 knockdown. Activation of Wnt/β-catenin signaling through R-spondin1, Wnt3a, or ΔN-β-catenin did not affect PTPN13/STAT1/MHC-I signaling activation. RNA-seq analysis showed that APC knockdown both upregulated the Wnt signaling pathway and downregulated the immune response, whereas CTNNB1 knockdown downregulated the Wnt signaling pathway but failed to restore the immune response. By contrast, Ptpn13 knockdown restored the immune response without affecting the Wnt signaling pathway. Furthermore, analysis of the TCGA dataset revealed a significant negative correlation between APC mutations and the immune markers CD3E, CD3G, CD4, and CD8A. By contrast, Wnt activity showed a significant positive correlation with these same immune markers, and CTNNB1 mutations did not substantially affect immune marker expression.

The role of PTPN13 in cancer has been controversial, with evidence suggesting both tumor-promoting and tumor-suppressive functions, depending on the context. PTPN13 was initially identified as a regulator of Fas-mediated apoptosis, and it modulates several signaling pathways by regulating the phosphorylation of surface receptors and intracellular substrates. In melanoma, cervical, and pancreatic carcinoma cells, PTPN13 has been implicated as an oncogene that acts by inhibiting Fas-mediated apoptosis.^27,44–48^ By contrast, PTPN13 has been shown to function as a tumor suppressor by stabilizing cell–cell junctions and inhibiting cell invasion in breast cancer,^49^ as well as by regulating IGF2BP1 and c-Myc expression in hepatocellular carcinoma.^50^ However, in our study, APC11 did not activate apoptotic pathways in CRC cells, suggesting that the APC/PTPN13/STAT1 axis is distinct from Fas-mediated apoptotic pathways.

Although the in vivo CRISPR screen in this study revealed genes potentially important for tumor growth, its limitations must be acknowledged. The screen achieved limited sgRNA coverage, and most candidate genes were identified on the basis of a single effective sgRNA, reducing statistical confidence. Nevertheless, this preliminary screen successfully identified PTPN13 as a high-priority candidate, which was subsequently confirmed through independent gain- and loss-of-function experiments.

Our findings suggest that APC functions as a master regulator of two critical signaling pathways: the Wnt/β-catenin cell proliferation pathway and the IFNγ/STAT1 cell immune response pathway. The coordination between these pathways may play an important role not only in tumorigenesis but also in maintaining intestinal homeostasis and repair following injury. For example, in the intestinal crypt, APC downregulation concurrently activates Wnt/β-catenin signaling, driving cell proliferation, and suppresses IFNγ/STAT1/MHC class I-mediated immune responses, enabling stem and transient amplifying cells to proliferate without immune surveillance. Further research is necessary to fully clarify these complex mechanisms and their implications for tumorigenesis and tissue homeostasis.

## Materials and methods

### Patients and clinical specimens

The tumor tissues of CRC patients were obtained from Nanfang Hospital, Southern Medical University. Patient information is presented in Supplementary information, Table S1. Written informed consent was obtained from all subjects. Subjects in our study were not involved in previous procedures and were drug or test naive. The study protocols concerning human subjects were consistent with the principles of the Declaration of Helsinki and were approved by the Clinical Research Ethics Committee of Southern Medical University.

APC mutation information and microsatellite instability status were obtained from clinical reports using the 3DMed Onco GPS Tissue Kit (3D Biomedicine Science & Technology Co., Limited, Shanghai) or determined by whole-exosome sequencing. In brief, genomic DNA was extracted from CRC tumor tissues using the QIAGEN DNeasy Blood and Tissue kit (Qiagen, Germany) and fragmented (180–280 bp) using Covaris DNA shearing (Massachusetts, USA). DNA libraries were constructed using the Agilent SureSelect Human All Exon V6 kit (Agilent Technologies, CA, USA) following the manufacturer’s instructions, and exons were captured by streptavidin magnetic beads and linearly enriched by PCR. Tagged libraries were purified (Beckman Coulter, Beverly, USA), quantified (Agilent Bioanalyzer 2100 system), and sequenced on the Illumina NovaSeq platform. Reads were quality-controlled using Fastp (v0.19.6) and then aligned to the human reference genome (GRCh38.p13) using BWA–MEM2 (v0.7.17). To detect mutations, SNP and InDel markers were detected using the best practices pipeline of GATK (v4.0.11.0) and annotated using SnpEff (v4.3). To analyze microsatellite instability, MSI scores were estimated using MSIsensor2 software^51^ and, as recommended, samples with MSI score ≥ 20% were considered MSI-high, whereas others were considered MSS.

### In vivo CRISPR screening in CT26 tumor cells

CRISPR screening was performed as previously reported by Doench et al.^52^ In brief, we created a Cas9-expressing CT26-shApc-GFP cell line and confirmed its high editing efficiency. For CT26-Cas9 cell line screening, we purchased a library of 130,209 sgRNAs targeting 20,611 genes (CRISPR-Pool KOUT Mouse, GENECHEM, Shanghai, China). The library contains at least 3 sgRNAs per gene and 1000 non-targeting control sgRNAs. The sgRNA library was delivered to CT26-Cas9-GFP cells via lentiviral infection at a multiplicity of infection (MOI) < 0.4. Transduced CT26-Cas9-GFP cells were expanded in vitro before subcutaneous implantation into mice. For each pool, CT26 cells were implanted into 15 nude mice and 15 WT Balb/c mice. Mice were euthanized when tumor size reached 2000 mm^3^, and tumors were resected, trypsinized to a single-cell suspension, and GFP sorted by FACS. Tumor genomic DNA was then prepared from whole tumor tissue using the QIAamp DNA Mini Kit (Cat. 51304). PCR was used to amplify the sgRNA region, and sequencing was performed on the Illumina HiSeq platform to determine sgRNA abundance. MAGeCK software^53^ and the “mageck test” command with default parameters were used to analyze CRISPR screen read counts and obtain sgRNA summary and gene summary tables. Significantly enriched or depleted sgRNAs were identified, ranked by robust rank aggregation (RRA) score, and visualized using MAGeCKFlute in R.

### Mice

C57BL/6, Balb/c, and nude mice were obtained from the Guangdong Medical Laboratory Animal Center or the Experimental Animal Center of Southern Medical University. The following mouse strains were purchased from Jackson Laboratory and were used to generate the mouse models in this study: B6.129S4-Kras^tm4Tyj^/J (*LSL-Kras*^*G12D*^), B6.129S7-Rag1^tm1Mom^/J (*Rag1*^*−/−*^), B6.129P2-Trp53^tm1Brn^/J (*p53*^*fl/fl*^), C57BL/6-Apc^tm1Tyj^/J (*Apc*^*fl/fl*^), C57BL/6J-Apc^*Min*^/J (*Apc*^*Min/*+^), and B6.Cg-Tg(Vil1-cre/ER^T2^)^23Syr^/J (Villin Cre-ER^*T2*^). B6/JGpt-Ptpn13^em1Cflox^/Gpt (Ptpn13^*fl/fl*^), in which exon 3–4 of the mouse *Ptpn13* gene was sandwiched by two loxP sequences, was purchased from GemPharmatech. Apc-HA mice were designed at the Shanghai Institute of Biochemistry and Cell Biology (CAS). All studies were performed in male mice, except for 4T1 subcutaneous xenograft and peritoneal metastasis models using female Balb/c and nude mice. Mice were kept in a standard 12-h light–dark cycle under specific-pathogen-free conditions and were allowed free access to water and food. Animal-related research protocols were consistent with the U.S. Public Health Service Policy on Use of Laboratory Animals and were approved by the Ethics Committee on Use and Care of Animals of Southern Medical University.

### Analysis of datasets

Transcriptome and somatic mutation data and the microsatellite instability status of TCGA-COAD and -READ were downloaded using the TCGAbiolinks^54^ R package. For mutation data, variants were called using the “varscan2” pipeline, and samples that harbored “frame shift del,” “frame shift ins,” “missense mutation,” “nonsense mutation,” “splice site mutation,” “in frame del,” “in frame ins,” “translation start site mutation,” or “nonstop mutation” on the APC gene locus were considered APC-mutated samples (APC-mut). For microsatellite instability status, samples were divided into MSI-High (MSI-H), MSI-Low (MSI-L), and MSS groups on the basis of “mononucleotide and dinucleotide marker panel analysis status”. Differentially expressed genes (DEGs) between APC-mut and APC-wild-type (APC-WT) samples of different microsatellite instability status were identified using the DESeq2 (1.38.3) R package. Genes with logFC ≥ 1 or ≤ −1 and *P* value ≤ 0.05 were considered DEGs. We used Euclidean distance and the ward.D2 clustering method to cluster samples and genes. For GSEA analysis, DEGs from APC-mut samples and APC-WT samples in the indicated groups were sorted by logFC and analyzed with the KEGG (c2) or GO-BP (c5) gene-set collections from MSigDB (v7.0, http://www.gsea-msigdb.org/gsea/msigdb). The clusterProfiler R package was used for GSEA analysis and visualization. GSEA results, corrected for multiple testing using the false discovery rate (FDR), are shown with a significance threshold of nominal *P* < 0.05. All R packages were run with default parameters unless otherwise specified.

To assess the effect of APC mutation on CRC with immunotherapy, the MSKCC dataset of 99 colorectal cancer patients was downloaded from the cBioPortal database (https://www.cbioportal.org).^24^ Survival analysis was performed using the Kaplan–Meier model to assess the effect of APC mutation on overall survival status.

### Cell culture and transfection

293 T, HCT8, HCT15, HCT116, HT29, SW480, SW620, LOVO, RKO, DLD1, NCW460 and Ls174.T cell lines were purchased from American type culture collection (ATCC) and maintained in our laboratory. R-spondin-1-transfected HEK 293 T cell line was purchased from Trevigen (3710-001-K). MC38, CT26, B16 and 4T1 cell lines were kindly gifted by W. Yang of Southern Medical University. All of the cell lines were maintained in DMEM (Gibco) with 10% FBS. For viral infection of cell, trypsinized cells were resuspended in 1 mL serum-free medium and plated in 12-well plates at high density (40%–50% confluent) for 12 h. Polybrene (10 μg/mL) was added to the medium followed by lenti-virus particles and cells were incubated for 12 h at 37 °C before exchanged to culture medium. Medium supplemented with Puromycin (10 μg/mL) or Neomycin (500 μg/mL) was administered for selection 3 days after transfection. Ptpn13 sgRNA transfected cells were sorted (Beckman coulter MoFlo XDF) as single cells into 96-well plates. Single-cell clones were randomly picked. By PCR and DNA sequencing, 1 clone without mutation was set as control (C1) and 2 clones with frame shift mutation (C2 and C3) were selected for further studies. Transfection efficiency was confirmed by quantitative reverse transcription polymerase chain reaction (qRT-PCR) or immunoblot assay. Lentivirus were used to knockdown Apc or Ctnnb1 (gene encoding β-catenin). siRNA sequences used were as follows: Apc-siRNA1 (GACTGTCCTTTCACCATATTT), Apc-siRNA2 (CGTGGATACTTTGTTACACTT), Ctnnb1-siRNA1 (CGCCATCACGACACTGCATAA), Ctnnb1-siRNA2 (CCATTGTTTGTGCAGTTGCTT). Annealed siRNA oligonucleotides targeting mouse APC or Ctnnb1 were cloned into GV112 (hU6-MCS-CMV-Puromycin) or GV117 (hU6-MCS-CMV-Neomycin) plasmid. The custom-made lentivirus for expressing Stat1^R274Q^, Irf1 or ∆N (aa 1–131)-Ctnnb1 were purchased from GENECHEM, Shanghai, China.

The gene deletions used in this study were created through the CRISPR–Cas9-mediated gene-editing system. To knockout PTPN13 or APC, sgRNAs were designed using the CRISPR tool (http://crispr.mit.edu) to minimize potential off-target effects. The following sgRNA sequences were used: 5’-CCTTAGAGCATTCACCGCAC-3’ and 5’-GCCATTCGAGACCGACTGAG-3’ to target mouse Ptpn13, 5’-CAGAGTCCAGCTACACGTG-3’ and 5’-TACACCTGCTGAATACGAG-3’ to target mouse Apc, 5’-ATGCAATGAATGAACTAGG-3’ and 5’-CTCTCTTAGGATTTGCCTG-3’ to target Human APC, 5’-ATGGGACGAGCAATCAGCAC-3’ and 5’-GTGAATACGAGTGTCAGACA-3’ to target Human PTPN13. Annealed sgRNA oligonucleotides were cloned into plasmid.

Lentivirus for expressing Cre-ER^T2^ recombinase (Cre-ER^T2^), loxp-U6-shApc and U6-sgRNA (Ptpn13)-EFS-NSp-Flex-spCAS9 were designed and constructed. When in the presence of 4-OHT, Apc expression can be rescued and Ptpn13 can be knocked out. Lentivirus carrying a catalytically inactive Cas9 (dCas9)-SAM transactivators and sgRNAs that target the Apc and Ptpn13 promoter were generated in GENECHEM, Shanghai, China.

Flag-tagged murine Stat1, was cloned into pENTER vector (Vigenebio). HA-tagged murine Apc, murine Apc^V2860A^, human APC, human APC^V2843A^ was cloned into pCDNA3.1 (Miaolingbio), respectively. Flag-tagged murine Ptpn13 with PDZ2a and PDZ2b-containing isoforms were cloned into pCMV (Miaolingbio). Flag-tagged PDZ2a (residues 1358–1459) and Flag-tagged PDZ2a (residues 1358–1464) was cloned into pCMV (Miaolingbio). siRNAs were used to knockdown murine Ptpn13, Ptpn11, Ptpn6, Ptpn2 and human Ptpn13, Ptpn11, Ptpn6, Ptpn2. Following siRNA sequences were used: Ptpn13-siRNA1 (GGATGATGTTAGTCTGATA), Ptpn13-siRNA2 (CGATGTTGGAGATGTTATA), Ptpn11-siRNA1 (GGCTGAGACCACAGATAAA), Ptpn11-siRNA2 (GAACCTTCATTGTGATTGA), Ptpn6-siRNA1 (GAGCAAGAAGGAAGAGAAA), Ptpn6-siRNA2 (GGTCACTCATATCAAGGTT), Ptpn2-siRNA1 (ACAGAGTGATGGTTGAGAA), Ptpn2-siRNA2 (AGAGAATAGGTTCAGAAGA), PTPN13-siRNA1 (GGATGATGTTAGTCTAATA), PTPN13-siRNA2 (GCATGAGACTACAAAGACA), PTPN11-siRNA1 (GGTCCAGTATTACATGGAA), PTPN11-siRNA2 (GGAGAACGGTTTGATTCTT), PTPN6-siRNA1 (GCAAGAACCGCTACAAGA), PTPN6-siRNA2 (GCACCATCATCCACCTCAA), PTPN2-siRNA1 (GTACAGGACTTTCCTCTAA), PTPN2-siRNA2 (GGAACAGAATAGGTCTAGA). The cell lipofection protocol was conducted according to manufacturer’s instruction. In short, cells were grown in the medium described earlier. Nucleic acid-Lipofectamine 3000 complexes were prepared according to the standard Lipofectamine 3000 protocol (Invitrogen). Lipofectamine 3000 reagent in Opti-MEM medium (Gibco), and plasmid in Opti-MEM medium were mixed together, incubated for 5 min, and added to the cells. Total cell lysates from cells were immunoprecipitated with indicated antibodies in the figure legends.

### Crypt isolation and organoid culture

Isolated small intestines were opened longitudinally and washed with cold phosphate-buffered saline (PBS) until the supernatant was clear. The tissue was chopped into ~5-mm pieces and incubated in a 50-mL conical centrifuge tube containing 30 mL shaking buffer (1 U/mL penicillin, 1 μg/mL streptomycin, and 2.5 ng/mL amphotericin B in PBS). The tube was shaken on a tube rotator (QB-208, Kylin-Bell) at 80 rpm for 10 min (4 °C). The tissue fragments were allowed to settle under normal gravity for 1 min. With the shaking buffer discarded, fragments were washed with 5 mL cold shaking buffer, and the shaking procedure was repeated. Intestinal fragments were transferred to 30 mL EDTA chelation buffer (15 mM EDTA in shaking buffer), shaken on a tube rotator for 30 min, and then oscillated vigorously for 20 s on a VORTEX-5 mixer (Kylin-Bell). The supernatant containing the villous fraction was discarded; the sediment was resuspended with shaking buffer and oscillated vigorously again for 2 min. The suspension was then centrifuged at 200× *g* for 10 min at 4 °C to collect intestinal crypts. Isolated intestinal crypts were counted using a hemocytometer, embedded in Matrigel (growth factor reduced, phenol red free; BD Biosciences) on ice, and seeded in 24-well plates (100 crypts per 50 μL of Matrigel per well). The Matrigel was polymerized for 30 min at 37 °C, then overlaid with 500 μL per well ENR culture medium (DMEM/F12, 2 mM Glutamax, 10 mM HEPES, 100 U/mL penicillin, 100 μg/mL streptomycin (Invitrogen), 1 mM N-acetyl cysteine (Sigma), B27 supplement (Invitrogen), N2 supplement (Invitrogen), 50 ng/mL mouse EGF (Peprotech), 100 ng/mL mouse Noggin (Peprotech), and 100 ng/mL R-spondin-1 (R&D Systems) or 10% human R-spondin-1-conditioned medium from R-spondin-1-transfected HEK 293T cells) or WENR (ENR medium supplemented with 50 ng/mL recombinant human Wnt-3A (R&D Systems), 20 μM CHIR99021 (Selleck), and 10 mM nicotinamide).^24^

### Viral infection of organoids

Organoids were cultured in WENR medium to enrich for stem cells. Before transfection, mouse organoids were trypsinized for 10 min at 37 °C to obtain single cells, then resuspended in 500 μL WENR medium plus Rho kinase inhibitor Y-27632 (Selleck). Cells were incubated with GFP- or Cre-encoding adenovirus (Vigenebio) for 6 h before plating in Matrigel (BD Biosciences). Cells were embedded in Matrigel on ice and seeded in 24-well plates. The Matrigel was polymerized for 30 min at 37 °C and overlaid with 500 μL per well WENR medium; after 3 days, WENR was exchanged for culture medium.

### Subcutaneous and orthotopic transplantation

CT26 cells (2 × 10^6^ cells, unless noted otherwise) were subcutaneously transplanted into right back flanks of Balb/c or nude mice. MC38 cells (2 × 10^6^ cells, unless noted otherwise) were subcutaneously transplanted into right back flanks of C57BL/6 or *Rag1*^*–/–*^ mice. Tumor length and width were measured with a caliper at the indicated times to calculate tumor volume (= width^2^ × length × 0.5). Mice were sacrificed when tumors reached 2000 mm^3^ in volume or when signs of ulceration were evident. Tumors were dissected, measured, and photographed, then embedded in OCT or fixed with formalin and embedded in paraffin for further immunohistological or immunofluorescence assessment. CT26 or MC38 cells (2 × 10^6^) were intraperitoneally injected into Balb/c or C57BL/6 mice for assessment of survival rate. For orthotopic transplantation of cells or organoids, digested cells (2 × 10^6^ cells, unless noted otherwise) were resuspended into 10% Matrigel in crypt culture medium, then transplanted into the colonic lamina propria of C57BL/6 recipient mice under optical colonoscopy using a customed injection needle (Hamilton Inc., 33-gauge, 6 inches long, point 4, 45-degree bevel) and syringe (Hamilton Inc. part number 7656-01). Optical colonoscopy was performed using an IKEDA HD Camera System and Karl Storz 1.9 mm/9.5 Fr Integrated Telescope (part number 1232 AA). Four injections were performed per mouse. Mice then underwent colonoscopy 40 days later to assess tumor formation. Colonoscopy videos and images were saved for offline analysis. After sacrifice of the mice, the distal colons were excised and fixed in 10% formalin, then examined by haematoxylin and eosin staining to identify tumors. Histology images were reviewed by gastrointestinal pathologists who were blinded to the treatment groups.

### In vivo competitive assay

Ptpn13 knockout CT26 cells were labeled with GFP and WT CT26 with mCherry (and vice versa), then 1:1 mixed, co-cultured, and expanded in vitro. This pool of CT26 cells was subcutaneously transplanted as described above into nude mice or WT Balb/c mice (2 × 10^6^ per mouse). This pool of CT26 cells was also grown in vitro for 7 days. Mice were euthanized, and tumors were resected and trypsinized to a single-cell suspension 14 days after tumor implantation. Tumor cells from 14 days of in vivo growth or 7 days of in vitro co-culture were FACS sorted to measure the GFP/mCherry ratio.

### Monoclonal antibody therapy

To deplete CD8^+^ T cells, mice were injected with 200 μg/dose anti-CD8α (BE0061, BioXcell) intraperitoneally twice weekly three days before tumor cell injection, with rat IgG2b isotype (BE0090, BioXcell) administered as a control.

Therapy using monoclonal antibodies was initiated when the tumor volumes reached 100 mm^3^. Anti-PD1 antibody (BE0146, BioXcell) was administered intraperitoneally every three days at a dose of 100 μg (Fig. 1) or 200 μg (Fig. 7) per mouse, with rat IgG2b isotype (BE0089, BioXcell) administered as a control.

### In vitro cytokine administration

Recombinant mouse IFNγ (#315-05, Peprotech) was reconstituted as described by the manufacturer to a concentration of 100 μg/mL in PBS with 0.1% bovine serum albumin (BSA). Administration of IFNγ is described in the figures and figure legends.

### CUT&Tag DNA library construction and ChIP-seq

The CUT&Tag assay was performed according to the manufacturer’s instructions (Novoprotein, N259-YH01). In brief, CT26 cells were stimulated with 0 and 100 ng/mL IFNγ for 2 h. Fresh cultured cells were harvested and incubated with concanavalin A-coated magnetic beads (Novoprotein, N251-01A) for 15 min. The bead-bound cells were permeabilized with 0.05% digitonin (Novoprotein, N253-YH01) and then incubated with primary p-STAT1 antibody (CST, 9167) on a rotating platform overnight at 4 °C. Goat anti-rabbit secondary antibody (Novoprotein, N269) was incubated with a primary antibody-cell-bead complex to increase the number of protein A binding sites. After removal of unbound antibodies by three washes, the complex was incubated with pA-Tn5 (Novoprotein, M059-YH01) and subjected to a transposition reaction. Next, cells were resuspended in fragmentation buffer and incubated at 37 °C for 1 h. The tagmentation was terminated by addition of 10% SDS at 55 °C for 10 min.

To amplify libraries, 35 µL DNA was mixed with 2.5 µL of a universal i5 primer and a uniquely barcoded i7 primer, using a different barcode for each sample; 10 µL of 5× AmpliMix was added and mixed. The samples were placed in a thermocycler with a heated lid using the following cycling conditions: 72 °C for 3 min; 98 °C for 30 s; 20 cycles of 98 °C for 15 s and 60 °C for 20 s; final extension at 72 °C for 2 min and hold at 10 °C. All the resulting libraries were sequenced on the Illumina NovaSeq 6000 platform in PE 150 mode by Shenzhen Chi Biotech. An average minimum of 20 million uniquely mapped reads per sample was obtained. Peaks were called using MACS2 version 2.2.7.1 with default parameters. All samples passed FastQC (v0.11.5) quality-control checks.

### APC^V2860A^ point mutation

Selectivity assessment in colony formation assays with clones derived from CT26 cells was engineered, and two clones bearing a point mutation in APC^V2860A^ were established as follows. The guide-RNA target sequence for APC (agacgtcacgaggtaagacccgg) was cloned into the pNGx_006 vector (pUC/ori, U6 promoter for tracrRNA/chimera, CMV promoter for SPyCas9 and puromycin selection). CT26 cells (5 × 10^5^) were electroporated with 1.5 μg of pNGx_006_sg APC and 0.5 μg of single-stranded oligonucleotide for APC^V2860A^ (GAGAGATTCGAAGACTGACAGCACAGAATCCAGTGGAGCCCAAAGTCCTAAACGCCATTCCGGATCTTACCTCGTGACGTCTGCTTAAaagagctagaaggacaaacgtagaaaagctggtgttaatcactactgctgtata) using a Neon Transfection System (Invitrogen) with the following parameters: voltage 1,300 V, pulse 20 ms, and pulse number 2. Monoclones were seeded after puromycin selection and characterized by Sanger sequencing.

### Pharmacokinetics assay

For pharmacokinetics assays, a polypeptide (iRGD-PEG2-(Lys)-FITC-PEG2-TSV) or nanoparticle-encapsuled polypeptide (Cy5, iRGD, TSV, HA2) was prepared in a series of dilutions and used to create a standards curve of concentration vs fluorescence intensity. The indicated peptides were administered at 10 μg/g (mouse weight) to wild-type C57/B6 via the caudal vein. Mouse blood was drawn at 0.25 h, 0.5 h, 1 h, 2 h, 4 h, 8 h, 12 h, 18 h, 24 h, 36 h, and 48 h after peptide administration and centrifuged at 5000 rpm at room temperature to collect serum for assays on a microplate reader (Cytation5; FITC, excitation 488 nm, emission 520 nm; Cy5, excitation 650 nm; emission 680 nm). Data are presented as the fluorescence intensity relative to that of serum from untreated mice at the indicated time point.

For tissue distribution assays, standard curves were created as described above. The indicated peptides were administered at 10 μg/g (mouse weight) to wild-type C57/B6 mice via the caudal vein. Mice were sacrificed at 2 h, 6 h, 12 h, 24 h, 36 h, and 48 h after peptide administration. Brain, heart, lung, spleen, kidney, intestine, and liver were harvested, weighed, and ground. Tissue homogenate was centrifuged at 5000 rpm at room temperature to collect supernatant for assays on a microplate reader (Cytation5). Data are presented as the fluorescence intensity relative to that of untreated mice at the indicated time point.

For metabolism assays, standard curves were created as described above. The indicated peptides were administered at 10 μg/g (mouse weight) to wild-type C57/B6 mice via the caudal vein. Mouse urine was collected at 0–2 h, 2–6 h, 6–2 h, 12–24 h, and 24–48 h after peptide administration and measured on a microplate reader (Cytation5). Data are presented as the fluorescence intensity relative to that of urine from untreated mice at the indicated time point, and the percentage of dose was calculated as the ratio of peptide concentration in urine/administered dosage in the indicated time frame.

For tissue target-specific assays, standard curves were created as described above. A polypeptide (iRGD-PEG2-(Lys)-FITC-PEG2-TSV) and control compound (FITC-PEG2-TSV) or a nanoparticle-capsuled polypeptide (Cy5, iRGD, TSV, HA2) and control compound (Cy5, TSV, HA2) were administered at 10 μg/g (mouse weight) via the caudal vein to CT26-shAPC tumor-bearing Balb/c mice when the tumor volume had reached 200 mm^3^. Mice were sacrificed at 0.5 h, 2 h, 4 h, 6 h, 8 h, 12 h, 18 h, 24 h, and 48 h after peptide administration. Subcutaneous tumors were harvested and weighed. Tumors were resected and ground to obtain a tissue homogenate, which was centrifuged at 5000 rpm at room temperature to collect supernatant for assays on a microplate reader (Cytation5). Data are presented as the fluorescence intensity relative to that of untreated mice at the indicated time point.

### Cytotoxicity and apoptosis assays

CCK-8 and apoptosis assays were performed to assess the cytotoxicity of the indicated peptides. Cells (CT26-shAPC/CT26-NC, CT26-shAPC-shPTPN13) were plated in 96-well plates (1 × 10^4^ cells/well) and cultivated for 12 h. TAT (25 μM), TAT-APC11 (50 μM), or TAT-APC11M (50 μM) peptides were added. After incubation for 48 h, the cells in each well were rinsed with PBS and treated with 100 μL cell culture medium containing 10 μL CCK-8 for 1 h, then assayed on a microplate spectrophotometer (Cytation5) at 450 nm.

For apoptosis assays, cells (CT26-shAPC/CT26-NC, CT26-shAPC-shPTPN13) were plated in 6-well plates (1 × 10^5^ cells/well) and cultivated for 12 h. TAT (25 μM), TAT-APC11 (50 μM), or TAT-APC11M (50 μM) peptides were added. After incubation for 48 h, the cells in each well were rinsed with PBS, and Annexin V-FITC and PI reagent were added (Elabscience, E-CK-A211) according to the manufacturer’s instructions. Cells were assayed on a microplate flow cytometer.

### RNA-seq and downstream bioinformatic analysis

Total RNA was isolated from the indicated mice subcutaneous tumor samples using QIAzol Lysis Reagent and quality checked. The transcriptome libraries were prepared following the instructions of the Illumina Stranded mRNA Prep, Ligation kit (San Diego, CA), and sequenced on the NovaSeq 6000 platform to obtain 150-bp paired-end reads. Raw reads were trimmed, quality controlled using fastp (v0.19.5), and aligned to the mice reference genome (mm10) using HISAT2 (v2.1.0). The mapped reads from each sample were assembled into transcripts using StringTie (v2.1.2). Downstream bioinformatic analyses were performed using R (v3.2.0) in RStudio (v4.0.3). DEGs were identified using the DESeq2 (v1.38.3) R package with default settings. GO analysis was performed using Database for Annotation, Visualization and Integrated Discovery (DAVID) tools.

### RNA isolation and RT-qPCR

Total RNA was isolated using TRIzol reagent (TaKaRa, Dalian, China) according to the manufacturer’s instructions, then reverse transcribed to complementary DNA using PrimeScript RT Master Mix (RR036A, TaKaRa). cDNA was diluted and used for quantification by real-time PCR using Tli RNaseH Plus mix (RR820A, TaKaRa) and performed on the 7500 Real-Time PCR system (Applied Biosystems). Relative mRNA abundance was calculated using ΔC_t_ values with *Hprt* mRNA as the internal control. Reactions for *Hprt* mRNA were performed concurrently on the same plate as those for the test mRNAs, and the results were normalized to the corresponding amount of *Hprt* mRNA. Expression level and fold change were calculated as follows: ∆CT = CT_gene of interest_ – CT_Hprt_; expression level = 2^−∆CT^; fold change = 2(∆CT_reference sample_ – ∆CT_tested sample_). The sequences of the PCR primers (forward and reverse, respectively) are 5’-TCTTTGCTGACCTGCTGGATT-3’ and 5’-ACTTTTATGTCCCCCGTTGACT-3’ for mouse Hprt; 5’-GCAGCTCAGCAAAAAGGGAAAT-3’ and 5’-TACATGGGGAGCACTGTCTCGT-3’ for mouse Axin2; 5’-CCTACTCGAAGACTTACCCAGT-3’ and 5’-GCATTGGGGTGAATGATAGCA-3’ for mouse lgr5; 5’-TATGTTCGGCTTCCCATTCTC-3’ and 5’-TTTCTGGTGCTTGTCTCACTG-3’ for mouse B2m; 5’-GGACTTGCCTTGTTCCGAGAG-3’ and 5’-GCTGCCACATAACTGATAGCGA-3’ for mouse Tap1; 5’-CTGGCGGACATGGCTTTACTT-3’ and 5’-CTCCCACTTTTAGCAGTCCCC-3’ for mouse Tap2; 5’-GGCCGATACAAAGCAGGAGAA-3’ and 5’-GGAGTTCATGGCACAACGGA-3’ for mouse Irf1; 5’-TGCTGTTCTGGTTGTCCTTG-3’ and 5’-CCTGGAGCCAGAGCATAGTC-3’ for mouse H2K1; 5’-TCTCTGTCGGCTATGTGG-3’ and 5’-CCTTGGCTTTCTGTGTTTC-3’ for mouse H2D1; 5’-GGGACAACCATCATGGCAGT-3’ and 5’-CAGCAGCGGAACCTGAGAG-3’ for mouse Lmp2; 5’-GCGAAAAGCAGAGCCCTAGAA-3’ and 5’-ACTATGGAGTTTGCGCCTTCC-3’ for mouse Apc; 5’-GCTGGAGATGATGACCGAGT-3’ and 5’-AACCGCTCCACATACAGTCC-3’ for mouse Myc; 5’-ATGGAGCCGGACAGAAAAGC-3’and 5’- CTTGCCACTCAGGGAAGGA-3’ for mouse Ctnnb1; 5’-GCTCCAAAGGACTTGTACGTG-3’ and 5’ -TGATCTGAAGGGCAGCATTTC-3’ for mouse Pdl1; 5’-GCGCCTTCGTTTTCCTATGTG-3’and 5’-AGGTTTTTGTTCACTGCTACGG-3’ for mouse Ptpn13; 5’-ATGACATCGCGGAGATGGTTT-3’ and 5’-GGGTTACTCTTACTGGGCCTT-3’ for mouse Ptpn11; 5’-GGACTTCTATGACCTGTACGGA-3’ and 5’-GCTGCGTGTAATACTCGACCA-3’ for mouse Ptpn6; 5’-GCAGTGAGAGCATTCTACGGA-3’ and 5’-TGACACAAACCCCATCTTAGTGA-3’ for mouse Ptpn2; 5’-GGAGTTCGAGGAACCCTAGTG-3’ and 5’-GGGATTTGTAGTGGATCGTGC-3’ for mouse Cxcl9; 5’-CCAAGTGCTGCCGTCATTTTC-3’ and 5’-GGCTCGCAGGGATGATTTCAA-3’ for mouse Cxcl10; 5’-GGCTTCCTTATGTTCAAACAGGG-3’ and 5’-GCCGTTACTCGGGTAAATTACA-3’ for mouse Cxcl11;

5’-GGACCTGACCTGCCGTCTAG-3’ and 5’-GTAGCCCAGGATGCCCTTGA-3’ for human GAPDH; 5’-TACGCTTTTGAGGGTTGATTC-3’ and 5’-GCAGGTTATTGCGAGTGTTTT-3’ for human APC; 5’-TGGCTATGTCTTTGCACCAG-3’ and 5’-TGTTTCTTACTGCCCACACG-3’ for human AXIN2; 5’-GTCAAGAGGCGAACACACAAC-3’ and 5’-TTGGACGGACAGGATGTATGC-3’ for human MYC; 5’-CCTACTCGAAGACTTACCCAGT-3’ and 5’-GCATTGGGGTGAATGATAGCA-3’ for human LGR5; 5’-GCTGTGGTCATCAGGCAGAGT-3’ and 5’-AGGTGAAAGACCAGAGCAGGAA-3’ for human IRF1; 5’-GGTTCTCACACCATCCAG-3’ and 5’-TAATCCTTGCCGTCGTAG-3’ for human HLA-A; 5’-GGGATGGCGAGGACCAAAC-3’ and 5’-ACAGCTCCGATGACCACAAC-3’ for human HLA-B; 5’-GATTTCTTCACACCTCTCCTT-3’ and 5’-CTCTCCACCTCCTCACATT-3’ for human HLA-C; 5’-CCAAGGAAGGCGTCTAAGGC-3’ and 5’-CTTTCGAGCGCAACCACTTTG-3’ for human B2M; 5’-TGCCCCGCATATTCTCCCT-3’ and 5’-CACCTGCGTTTTCGCTCTTG-3’ for human TAP1; 5’-AATCCCTCACTATTCTGGTCGT-3’ and 5’-TCGAGACATGGTGTAGGTGAAG-3’ for human TAP2; 5’-GGTTCTGATTCCCGAGTGTCT-3’ and 5’-CAGCCAAAACAAGTGGAGGTT-3’ for human LMP2; 5’-GGACAAGCAGTGACCATCAAG-3’ and 5’-CCCAGAATTACCAAGTGAGTCCT-3’ for human PD-L1; 5’-GAAGAGTTGGATACTCAGCGTC-3’ and 5’-TGCAGTTTAACACGACTGTGAT-3’ for human PTPN2; 5’-TGAACTGCTCCGATCCCACTA-3’ and 5’-CACGCACAAGAAACGTCCAG-3’ for human PTPN6; 5’-GAACTGTGCAGATCCTACCTCT-3’ and 5’-TCTGGCTCTCTCGTACAAGAAA-3’ for human PTPN11; 5’-ACTGCTTGGAATGTGTGAGGA-3’ and 5’-CACGTAGGAAAATGAGGGTGC-3’ for human PTPN13.

PDZ2a PDZ2b were identified via PCR, with the PCR primers (forward and reverse, respectively), 5’-ATTCTACCATCAGACTCTGC-3’ and 5’-TGAACTGGCTAAAGGTGATA-3’ for human PTPN13; 5’-ATTCTGCCATCTGACTCTGC-3’ and 5’-TGAGCTGGCTAAAACTGATG-3’ for mouse Ptpn13. *Ptpn13*^*fl/fl*^ mice were genotyped using PCR with specific primers: 5’-GCAGATTGACCTGTTGTTTGGG-3’ and 5’-TTAGGTTACATGGGGACTGGAGC-3’.

### Co-immunoprecipitation and immunoblot analysis

For immunoprecipitation, cells were lysed on ice in IP lysis buffer supplemented with protease and phosphatase inhibitors for 30 min. Lysates were cleared by centrifugation at 15,000× *g* for 30 min at 4 °C, and supernatants were incubated with the indicated antibodies for 12 h at 4 °C. After incubation, Protein A/G PLUS-Agarose (SC-2003, Santa Cruz Biotechnology) was added and incubated for 3 h at 4 °C. Immune complexes were purified with extensive washing buffer (5.6 mM Na_2_HPO_4_, 8.0 mM KH_2_PO_4_, 96.2 mM NaCl, 1.6 mM KCl, and 1 mM PMSF). Purified complexes were mixed with loading buffer and assayed by western blot. The antibodies used were as follows: anti-APC (1:100, #ab15270, abcam), anti-PTPN13 (1:1000, #NB100-56139, Novus), anti-Flag (1:1000, #F1804, Sigma), Anti-DLG1 (1:500, #ab300481, abcam), Anti-PTPN2 (1:200, #M01597, BOSTER), Anti-SHP1/PTPN6 (1:500, #M00938-2, BOSTER), Anti-SHP2/PTPN11 (1:500, #M00150-2, BOSTER), Anti-ZO-1 (1:500, #21773-1-AP, Proteintech), Anti-PTPN13 (1:500, #25944-1-AP, Proteintech), and Anti-CTNNB1 (1:500, #610153, BD Biosciences).

For immunoblotting, total protein was extracted with lysis buffer (#P0013, Beyotime Biotechnology). Protein concentration was determined using a BCA assay kit (#23227, Thermo Fisher Scientific). Cell lysates were separated by 4%–5% Tris-glycine gel electrophoresis, transferred to polyvinylidene difluoride membranes (#HVHP29325 or #GVHP29325, Millipore), and incubated overnight at 4 °C with the appropriate primary antibodies: anti-APC (1:100 dilution, #ab15270, abcam), anti-Phospho-JAK1 (1:100, #3332S, Cell Signaling Technology), anti-JAK1 (1:100, #3331S, Cell Signaling Technology), anti-Phospho-STAT1 (1:100, #9167S, Cell Signaling Technology), anti-STAT1 (1:500, #9172S, Cell Signaling Technology), anti-IRF1 (1:500, #8478S, Cell Signaling Technology), anti-Caspase-8 monoclonal antibody (1:500, 66093-1-IG-50UL, Proteintech), anti-Caspase-3 antibody (1:500, #9662S, Cell Signaling Technology), and anti-Cleaved Caspase-3 (1:200, #9664 T, Cell Signaling Technology). Signals were detected using horseradish peroxidase (HRP)-conjugated secondary antibodies and SuperSignal West Femto Chemiluminescent Substrate (34096, Thermo Fisher Scientific). Images were captured using an automated chemiluminescence imaging analysis system (Tanon 5200).

### PTPase assay

For in vitro PTPase assays, IFNγ (100 ng/mL) was used to stimulate Flag-Stat1-plasmid-transfected 293T cells for 1 h. Cell lysates were obtained and immunoprecipitated using anti-Flag antibody. Immunoprecipitates were incubated with GST-tagged PTPase (#ab42581, Abcam) in 25 mM HEPES (pH 7.5), 5 mM EDTA, and 10 mM DTT at 37 °C for 3 h, then immunoblotted with anti-phospho-STAT1 (1:100, #9167S, Cell Signaling Technology) and anti-STAT1 (1:500, #9172S, Cell Signaling Technology).

### Immunofluorescence

Frozen tissues were used to obtain 5-μm sections for staining as described previously.^24,55^ Paraffin blocks were prepared using standard methods, and 2.5-μm tissue sections were obtained. The sections were de-waxed and stained with anti-mouse CD8 (1:500, #ab209775, abcam). The secondary antibodies and fluorescent reagents used for CD8 detection were goat anti-rat IgG with fluorescein-Cy3 (Perkin Elmer). DAPI was used to stain nuclei. Cover slips were mounted using ProLong Gold anti-fade medium. Slides were imaged using a Carl Zeiss LSM 880 inverted laser scanning confocal microscope. The total number of CD8-positive cells per 5 microscopy fields (200×) was calculated. For organoid staining, organoids were grown in 40 μL of Matrigel plated into an 8-well chamber slide (Lab-Tek II, 154534). Where indicated, BSA or 100 ng/mL IFNγ was added to the growth medium for 24 h before fixing. The growth medium was removed, and the cells were fixed in 4% PFA-PME (50 mM PIPES, 2.5 mM MgCl_2_, and 5 mM EDTA) for 20 min. They were then permeabilized in 0.5% Triton for 20 min and blocked in IF buffer (PBS, 0.2% Triton, 0.05% Tween, and 1% BSA) for 1 h. Cells were incubated in anti-IRF1 primary antibody (1:200, #13063, Cell Signaling Technologies) overnight in IF buffer, then washed three times with TBS 0.1% Tween. Alexa Fluor 594-labeled goat anti-rabbit secondary antibodies in PBS containing 1% BSA were added and incubated for 90 min. The solution was removed, DAPI in PBS was added for 5 min, and the cells were washed twice with TBS 0.1% Tween. The chambers were then removed, and cover slips were mounted using ProLong Gold anti-fade medium. Images were acquired using a Carl Zeiss LSM 880 inverted laser scanning confocal microscope. For cell staining, cells were treated with or without 100 ng/mL IFNγ for 24 h before harvest. Cells were then fixed for 10 min at room temperature with 4% paraformaldehyde in PBS and permeabilized with 0.1% Triton X-100 in PBS for 10 min at room temperature. Cells were then incubated overnight at 4°C with primary antibodies to IRF1 (1:200, #8478S, Cell Signaling Technology) and then with Alexa Fluor 594-labeled goat anti-rabbit secondary antibodies for 90 min at room temperature in PBS containing 1% BSA. Cells were covered with a drop of ProLong Gold antifade reagent with DAPI for observation. Slides were imaged using a Carl Zeiss LSM 880 inverted laser scanning confocal microscope.

### Immunohistochemistry

Frozen tissues were used to obtain 5-μm sections for subsequent staining as described previously.^24,55^ Staining was performed using anti-CD8 antibody (1:200, #100716, BioLegend). Secondary antibodies and fluorescent reagents used for CD8 detection were goat anti-rat IgG with HRP. Subcutaneous tumors were fixed by incubation in formalin overnight at room temperature and then dehydrated. Paraffin blocks were prepared using standard methods, and 2.5-μm tissue sections were obtained. The sections were de-waxed and stained with anti-mouse CD8 (1:500, #ab209775, abcam), anti-human CD8 (1:500, #HPA037756, ATLAS), anti-Ki67 (1:1000, #550609, BD Biosciences), or anti-CD31 (1:200, #77699S, Cell Signaling Technology) primary antibody in combination with HRP-conjugated secondary goat anti-mouse antibody. All slides were detected by adding DAB substrate at room temperature, and Mayer’s hematoxylin was used to highlight nuclei. Sections were dehydrated and mounted in neutral balsam mounting medium. A coverslip was placed over the tissue section, and the section was photographed under an Olympus BX53F microscope.

### Multiplexed immunofluorescence staining

Paraffin blocks were prepared using standard methods, and 2.5-μm tissue sections were obtained. The sections were de-waxed and hydrated. Endogenous peroxidase activity was blocked by immersing the slides in peroxidase blocking buffer (0.040 M citric acid, 0.121 M disodium hydrogen phosphate, 0.030 M sodium azide, and 1.5% hydrogen peroxide) for 15 min at room temperature. Antigen retrieval involved boiling for 10 min in Tris-EDTA (pH 9.0) and incubation for 30 min in blocking buffer (5% goat serum) at room temperature. Staining for (CK, CD3 and CD8) or (Epcam, HLA-ABC and CD8) or (APC, PTPN13 and p-STAT1) was performed using a sequential multiplexed immunofluorescence protocol with the isotype-specific primary antibodies anti-CK (1:800, #C2562, Sigma-Aldrich), Epcam (1:500, #93790S, Cell Signaling Technology), CD3E (1:500, #HPA043955, ATLAS), CD8 (1:500, #HPA037756, ATLAS), HLA-ABC (1:200, #565292, BD Biosciences), APC (1:100, #ab15270, abcam), PTPN13 (1:500, #NB100-56139, Novus) and p-STAT1 (1:50, #9167S, Cell Signaling Technology). Nuclei were highlighted using DAPI. The secondary antibodies and fluorescent reagents used were goat anti-rabbit IgG with fluorescein-tyramide (Perkin Elmer) for Epcam and PTPN13 detection, goat anti-rabbit IgG with Cy3-tyramide (Perkin Elmer) for APC, HLA-ABC, and CD3 detection, and goat anti-rabbit IgG with Cy5-tyramide (Perkin Elmer) for CD8 and p-STAT1 detection. Residual HRP activity between incubations with secondary antibodies was eliminated by exposing the slides to a solution containing benzoic hydrazide and hydrogen peroxide two times for 7 min each.

### Flow cytometry

For flow cytometric analysis of MHC-I, the indicated cells were pretreated with IFNγ (100 ng/mL) or BSA for 24 h. Washed cells were resuspended in staining buffer (PBS with 1% BSA). Anti-mouse H-2Kb/SIINFEKL (1:200, #756311, BD OptiBuild) or anti-H-2Kd/2Dd Class I (1:200, #12-5998-81, eBioscience) antibody or isotype control antibody (1:200, #12-4724-81, eBioscience) was added, and staining was continued for 40 min on ice. To analyze tumor-infiltrating lymphocytes, the indicated tumors were cut, digested in DMEM with 0.1 mg/mL DNase I (Solarbio, D8071), 100 U/mL collagenase I (Solarbio, C8140), and 100 U/mL collagenase IV (Solarbio, C8160), and shaken at 37 °C for 30–50 min. Each single-cell suspension was passed through a 70-μm filter, centrifuged, pelleted, and rinsed with PBS. Cells were incubated with Fc Blocker (clone 93; BioLegend) for 20 min on ice. Where indicated, anti-CD45-PE antibody (30-F11, eBioscience, 12-0451-82), anti-TCR-β-APC (H57-597, eBioscience, 17-5961-82), anti-CD4-PE Cy5.5 (RM4-5, PE-Cyanine5.5, eBioscience 17-5961-82), anti-CD8-APC cy7 (53-6.7, APC-eFluor 780, eBioscience 0081-82), anti-Flex-T Biotin H-2 K(b) OVA Monomer (SIINFEKL, bioLegend 280051, tetramers generated following the manufacturer’s instructions), or isotype control antibody (1:200, #12-4724-81, eBioscience) was added, and staining was continued for 40 min on ice. For IFNγ and TNFα staining, the indicated cells were restimulated with Cell Activation Cocktail (with brefeldin A) (bioLegend, cat#423303) for 4 h. Additional fixation and permeabilization steps were performed following the manufacturer’s instructions (BD Fixation/Permeabilization Kit, #554714), followed by incubation with anti-IFN-γ (XMG1.2, BV650, BD Pharmingen 563854) or anti-TNF-α (BV650, bioLegend, 506327) antibody for 40 min on ice. After a wash step, cytometry sample acquisition was performed on an LSRFortessa X-20 flow cytometer (BD), and analysis was performed using FlowJo software (TreeStar). Cells stained with the isotype control antibody were used for gating.

### Protein purification

cDNAs encoding the KIND (residues 3–190), FERM (residues 572–872), PDZ1 (residues 1093–1178), PDZ2a (residues 1368–1459), PDZ3 (residues 1501–1588), PDZ4 (residues 1788–1868), and PDZ5 (residues 1882–1965) domains of human PTPN13 and a cDNA encoding STAT1 were individually cloned into the pGEX6P1 vector (GE Healthcare) and expressed and purified with standard techniques. All proteins were overexpressed in *E. coli* strain BL21, which was cultured at 37 °C in LB medium and incubated for 20 h at 18 °C with 0.5 mM isopropyl β-D-1-thiogalactopyranoside (IPTG) upon reaching OD_600_ = 0.6. Cells were harvested by centrifugation and resuspended in binding buffer (GST column binding buffer: 25 mM Tris-HCl (pH 8.0), 150 mM NaCl, and 2 mM DTT). The cells were lysed by sonication, followed by centrifugation. Supernatants of the cell lysates were purified using the GST Spin Purification Kit (#16107, Thermo Fisher Scientific) according to the manufacturer’s instructions. His-STAT1 was purchased from Prospec (PKA-315).

The N-terminally 6× His-tagged and SUMO-tagged PTPN13 PDZ2a domain (1362–1452) was cloned and inserted into the pET28a vector. Following overnight protein expression induced by 0.1 mM IPTG at 16 °C, cell pellets were lysed by sonication, and supernatants were applied to Ni Sepharose 6 Fast Flow resin (Cytiva). The eluted fractions were dialyzed in buffer containing 20 mM Tris (pH 8.0) and 150 mM NaCl for 3 h, followed by excision of the 6× His-SUMO tag with ULP1 protease. The protein was then loaded onto a Superdex 200-pg gel filtration column (GE Healthcare) equilibrated in 20 mM Tris (pH 6.8) and 150 mM NaCl for further purification.

### Peptide synthesis and administration

APC5: LVTSV, APC7: SYLVTSV, APC9: SGSYLVTSV, APC11: RHSGSYLVTSV, APC13: PKRHSGSYLVTSV, APC15: QSPKRHSGSYLVTSV, PDZ2a with or without an HA tag, the cell-penetrating peptide (TAT, with GRKKRRQRRR sequence)-conjugated C-terminal 5, 7, 9, 11, 13, or 15 residues of human WT APC or mutant APC (with sequence RHSGSYLVTSA), or the fusogenic HA2 peptide with the sequence GDIMGEWGNEIFGAIAGFLG were chemically synthesized and purified by reversed-phase HPLC (GL Biochem Ltd.). For cell-peptide treatments, cells were incubated with 25 μM TAT-HA2 with or without 50 μM TAT-APC11 or TAT-APC11M for 2 h, treated with IFNγ at the indicated concentrations and times, and then subjected to qRT-PCR, immunoblot analysis, or flow cytometry. For in vivo assays, cells maintained in DMEM with 10% FBS were incubated with 25 μM TAT-HA2 with or without 50 μM TAT-APC11 or TAT-APC11M peptides for 4 h and then trypsinized. Digested cells were resuspended into PBS with 25 μM TAT-HA2 and with or without 50 μM TAT-APC11 or TAT-APC11M peptides, then subcutaneously transplanted into mice.

### Surface plasmon resonance (SPR)-based binding assays

According to the manufacturer’s instructions, the 3D Dextran chip was activated using EDC (76.5 mg/mL) and sNHS buffer (11.5 mg/mL). Next, 2 μg PDZ2a peptide was immobilized onto the 3D Dextran chip surface. The peptide was incubated overnight at 4 °C at a humidity greater than 60%. Remaining active parts were deactivated in 1 M ethanolamine for 5 min. The binding kinetics between human APC fragments of different lengths (500 μM) and immobilized PDZ2a were assessed using a PlexArray HT A100 instrument (Plexera) at a flow rate of 2 μL/s, with association and dissociation for 300 s each. Changes in the frequency of the sensor surface resonance (ΔF) during the binding experiments were recorded using Plexera Instrument Control 1.9.0 software. The response unit (RU) value was derived using Plexera Data Explorer software (Plexera) and Biacore T200 Evaluation software v1.0 (GE Healthcare). To obtain values for the specific binding response, background binding to the reference surfaces was subtracted from binding to the experimental surfaces.

### Fluorescence polarization (FP) binding assay

APC peptides fluoresceinated at their N termini with FITC (APC9: SGSYLVTSV; APC11: RHSGSYLVTSV; APC13: PKRHSGSYLVTSV; APC15: QSPKRHSGSYLVTSV; APC11 mutant: RHSGSYLVTSA at the last residue) were purchased from GL Biochem (Shanghai) Ltd. For the binding assay, the concentration of FITC labeled peptides was maintained at 1.7 nM, and PTPN13-PDZ2 proteins were diluted in a 2-fold series in binding buffer containing 20 mM Tris (pH 6.8) and 150 mM NaCl. The FP signals were recorded using black 384-well plates on a Synergy H1 microplate reader (BioTek Instruments), and *K*_d_ values were calculated by fitting curves in GraphPad Prism 9.

### Structure prediction of the APC–PDZ2a interaction

The complex structure of APC–PDZ2a was predicted with Alphafold3^56^ using its public server (https://alphafoldserver.com/); it was modeled using the full-length APC sequence (2843 aa) and the PTPN13 PDZ2a domain (residues 1368–1459) as input. The structure was visualized using PyMol (The PyMol Molecular Graphics System).

### Crystallization and X-ray data collection

PTPN13 PDZ2a–APC11 protein complexes were crystallized using a sitting-drop vapor diffusion method at 16 °C. APC11 peptide-bound PDZ2a co-crystals were obtained by mixing an equal volume of reservoir solution containing 0.1 M citric acid (pH 3.5) and 3 M NaCl with 38 mg/mL PDZ2a proteins in the presence of 5.5 mM APC11 peptide. Before flash freezing, 25% glycerol was added to the reservoir solution to cryoprotect the crystals. Diffraction data were collected at Shanghai Synchrotron Radiation Facility Beamline 19U1 and processed using the HKL3000 program.^1,57^

### Structure determination and refinement

The complex structure of PDZ2a–APC11 was solved by molecular replacement with the program Phaser^58^ using the apo PDZ2a structure (PDB code 3LNX^32^) as the search model. Further manual model building was facilitated using Coot,^59^ combined with structure refinement using Phenix.^60^ The diffraction data and final statistics are summarized in Supplementary information, Table S2. All structure figures were prepared using PyMol (The PyMol Molecular Graphics System).

### GST pull-down

GST pull-down assays between His-STAT1 and GST-tagged PTPN13-domain proteins were performed according to standard procedures. His-STAT1 protein (PKA-315, PROSPEC) was added to GST-tagged PTPN13-domain proteins (20 μg each) pre-immobilized on a GST affinity column at 4 °C, then washed using GST column binding buffer (25 mM Tris-HCl (pH 8.0), 150 mM NaCl, and 2 mM DTT). The bound proteins were eluted with GST column elution buffer (50 mM Tris-HCl (pH 8.0), 150 mM NaCl, and 7 mM glutathione), then analyzed by SDS-PAGE and immunoblotting using anti-GST (1:1000, #66001-2-Ig, Proteintech) and anti-His (1:1000, #66005-1-Ig, Proteintech) antibodies. For the interaction between GST-STAT1 and HA-PDZ2a, 20 μg TAT-APC11 or TAT-APC11M peptide was added to 500 μL binding buffer with recombinant GST-STAT1 (20 μg) and HA-PDZ2a (20 μg) and incubated with GST-Sepharose beads for 12 h at 4 °C. Following incubation, the bound proteins were eluted and purified with elution buffer. Purified complexes were mixed with loading buffer and assayed by western blotting using anti-GST (1:1000, Proteintech, #66001-2-Ig) and anti-HA (1:1000, Abcam, #ab18181) antibodies.

### Synthesis and administration of PEG-TAT-APC11 peptides

PEG-conjugated TAT-APC11 peptide was chemically synthesized and then purified by reversed-phase HPLC (GL Biochem Ltd.). The dosage of PEG-conjugated TAT-APC11 was determined by the RHSGSYLVTSV peptide (200 μg per mouse). The peptides were intraperitoneally injected into the mice every other day. Anti-PD1 antibody (BE0146, BioXcell) was administered intraperitoneally every three days (200 μg/dose), with rat IgG2b isotype (BE0089, BioXcell) administered as a control. The timing and frequency of nanoparticle and anti-PD1 antibody injections are noted in the Figures, and the survival times of mice were recorded.

### Nanoparticle synthesis, assembly with peptides, and administration

For every 5 mg of SDS, 186 μL 2-(2-methoxy ethoxy) ethyl methacrylate (MEO2MA), 1.8 μL methylacrylic acid (MAA), and 9.8 μL ethylene glycol methacrylate (EGDMA) were dissolved in 20 mL ddH_2_O and heated to 70 °C under a nitrogen atmosphere. Five milligrams of KPS solution in 1 mL ddH_2_O was then mixed in and stirred for 6 h. When the reaction had cooled to room temperature, it was centrifuged at 50,000 rpm for 10 min. The precipitate was collected, rinsed 3 times, and resuspended in PBS. For every 150 mg of nanoparticles in 100 mL PBS, 6 mg EDC (N1-((ethylimino)methylene)-N3,N3-dimethylpropane-1,3-diamine) and 10 mg CRGDKGPDC cyclopeptide (iRGD)^35^ were added and allowed to react for 4 h at room temperature. The mixture was then centrifuged at 50,000 rpm for 10 min, and the nanoparticles were collected, rinsed, and resuspended in PBS. Ten milligrams of GDIMGEWGNEIFGAIAGFLG peptide was added to the nanoparticles; after 6 h at room temperature, the mixture was centrifuged at 50,000 rpm for 10 min, rinsed, and dispersed in PBS. In addition, for the treatment group, 60 mg RHSGSYLVTSV peptide was added, allowed to react overnight at room temperature, centrifuged at 50,000 rpm for 10 min, rinsed, and dispersed in PBS. The concentration of RHSGSYLVTSV peptide was calculated by subtracting the amount of peptide remaining in the solution from the total amount of peptide added. The dosage of polypeptide-conjugated nanoparticles was determined by RHSGSYLVTSV peptide (200 μg per mouse). Nanoparticles were injected via the caudal vein every other day, and 200 μg/dose anti-PD1 antibody (BE0146, BioXcell) was injected intraperitoneally every three days, with rat IgG2b isotype (BE0089, BioXcell) administered as a control, when the subcutaneous tumor volume reached 100 mm^3^ or the day after orthotopic transplantation or when the *Apc*^*Min/+*^ mice were 9 weeks old. The timing and frequency of nanoparticle and anti-PD1 antibody injection are noted in the Figures.

### Statistical analysis

Statistical analyses were performed using GraphPad Prism software 9.0 (CA, USA) and SPSS software v22.0 (IBM Corp., Armonk, N.Y., USA). Comparisons between multiple treatment groups and a control group were analyzed using one-way ANOVA or two-way ANOVA, followed by Dunnett’s multiple comparison test. LSD, Tukey’s, or Dunnett’s T3 multiple comparison test was used for comparisons between groups after one-way ANOVA according to the homogeneity of variance where pairwise comparisons were required for each group to analyze tumor volume. Two-way ANOVA followed by Tukey’s multiple comparison test was used where pairwise comparisons were required for each group to analyze tumor growth. A linear regression model was used where required to assess effect of MSI-H (MSS + MSI-L as reference), APC mutation (APC WT as reference), KRAS mutation (KRAS WT as reference), CTNNB1 mutation (CTNNB1 WT as reference) and Wnt activity score on the T cell markers CD3E, CD3D, CD3G, CD4, and CD8A. Survival time of mice was analyzed using log-rank analysis. Detailed statistical parameters and methods are described in the Figures and Figure Legends. A value of *P* < 0.05 was considered statistically significant.

## Supplementary information

Supplementary Figure S1

Supplementary Figure S2

Supplementary Figure S3

Supplementary Figure S4

Supplementary Figure S5

Supplementary Figure S6

Supplementary Figure S7

Supplementary Figure S8

Supplementary Figure S9

Supplementary Figure S10

Supplementary Table S1

Supplementary Table S2

Supplementary Table S3

## Data Availability

RNA-seq data and somatic mutation data for colorectal cancer were downloaded from TCGA using the R package TCGAbiolinks. DEGs of APC-mut samples and APC-WT samples were sorted by logFC, and analyzed using KEGG (c2) or GO-BP (c5) gene-set collections from MSigDB (v7.0, http://www.gsea-msigdb.org/gsea/msigdb). For GEO data analysis, dataset GSE67186 was downloaded from the GEO database (https://www.ncbi.nlm.nih.gov/geo/query/acc.cgi?acc=GSE67186). Data generated in this study are included within the paper (and its supplementary information files) or are available from the corresponding authors upon reasonable request. Source data are provided with this paper. Coordinates and structure factors of the PTPN13 PDZ2a–APC11 complex have been deposited in the Protein Data Bank under accession number 7XTY.

## Code availability

No custom code or mathematical algorithm that includes any access restrictions was used in this study.
