# Supplementary material for: Targeting PTPN13 with 11-amino-acid peptides of C-terminal APC prevents immune evasion of colorectal cancer
Source: Cell Res. 2026 Jan 5;36(1):72–93. doi: 10.1038/s41422-025-01206-4 (PMC12765898; doi:10.1038/s41422-025-01206-4)
Supplement: Supplementary file 1 — Supplementary Figure S1 [file 41422_2025_1206_MOESM1_ESM.pdf]

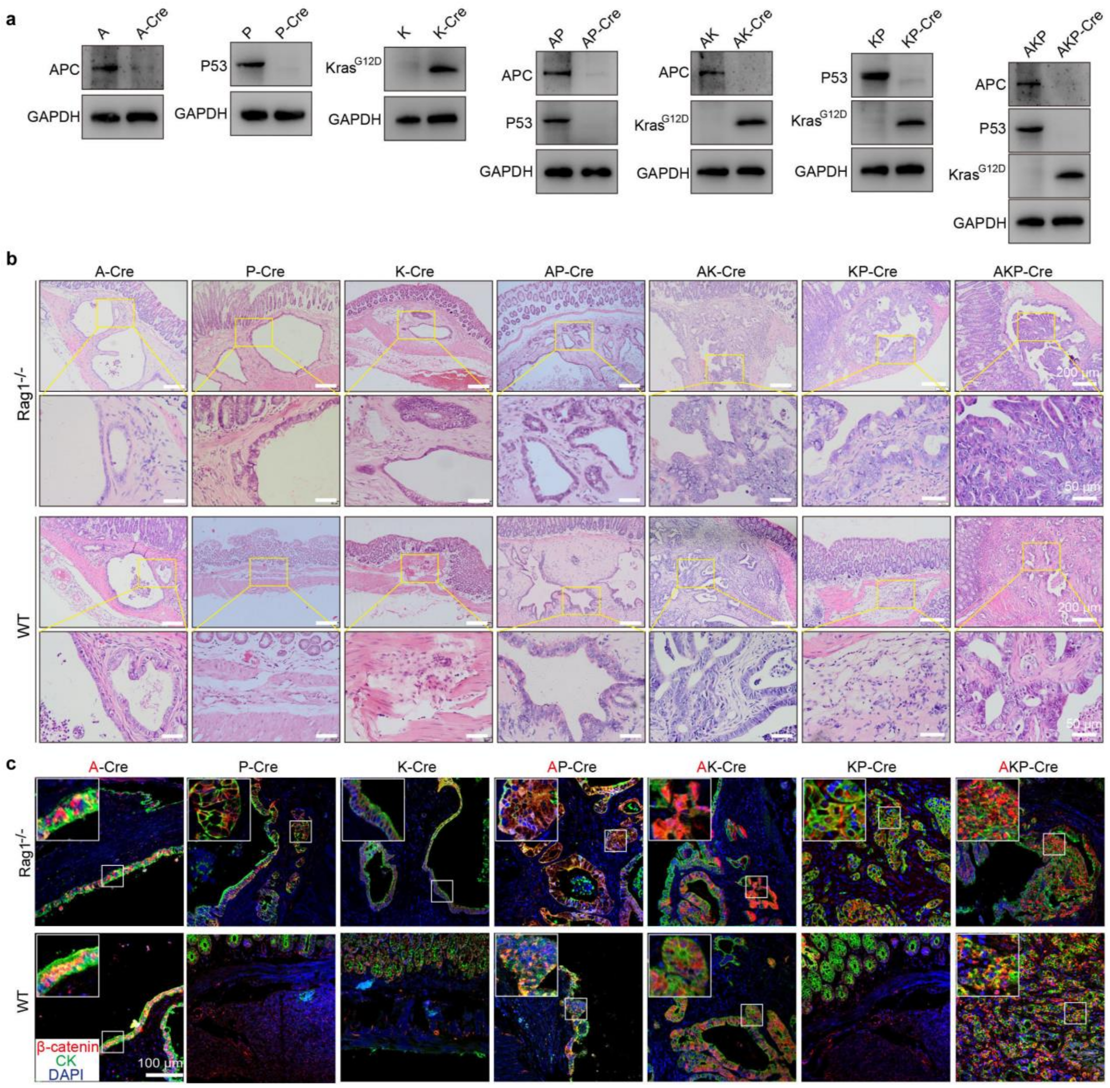

Supplementary information, Fig. S1. Additional data on tumor formation by APC-deficient intestine organoids. a, Total cell lysates of indicated organoids from $Apc^{fl/fl}$ (A), $p53^{fl/fl}$ (P), $LSL\text{-}Kras^{G12D}$ (K), $Apc^{fl/fl}/p53^{fl/fl}$ (AP), $Apc^{fl/fl}/Kras^{G12D}$ (AK), $p53^{fl/fl}/Kras^{G12D}$ (KP) and $Apc^{fl/fl}/p53^{fl/fl}/Kras^{G12D}$ (AKP) mice were subjected to immunoblot analysis with antibodies to the indicated proteins. Data represents three independent experiments. **b,** Histology slides showing representative haematoxylin and eosin (H&E) staining of orthotopic transplantation from indicated organoids. The intestinal epithelial boundary was marked with a black dashed line. **c,** Representative images showing immunofluorescence staining against β-catenin (red), CK (green) and nucleus (DAPI) in orthotopic tumor sections formed by indicated A, P, K, AP, AK, KP, AKP intestine organoids transfected with adenovirus expressing Cre recombinase.
