# Supplementary material for: Targeting PTPN13 with 11-amino-acid peptides of C-terminal APC prevents immune evasion of colorectal cancer
Source: Cell Res. 2026 Jan 5;36(1):72–93. doi: 10.1038/s41422-025-01206-4 (PMC12765898; doi:10.1038/s41422-025-01206-4)
Supplement: Supplementary file 2 — Supplementary Figure S2 [file 41422_2025_1206_MOESM2_ESM.pdf]

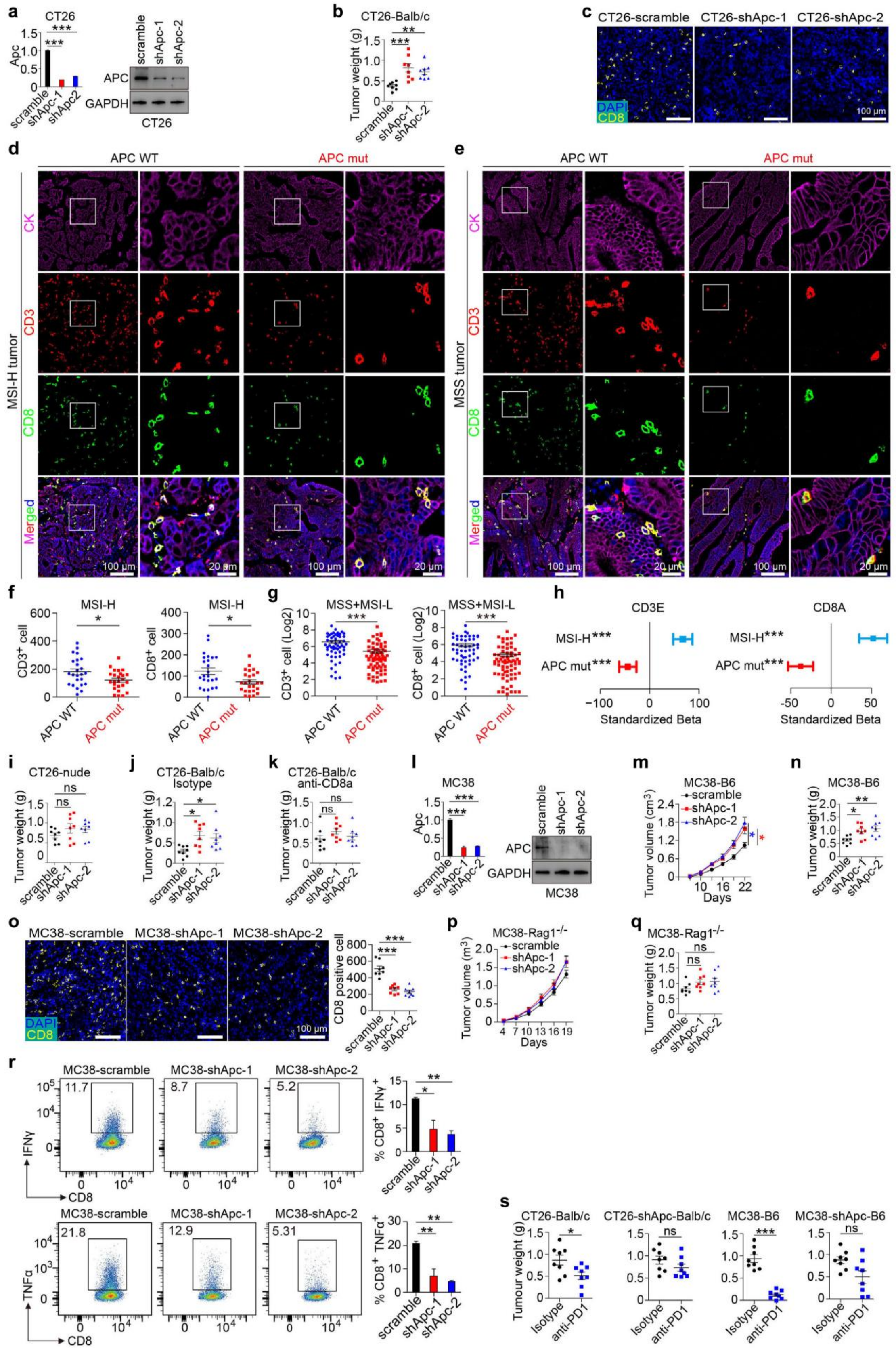

Supplementary information, Fig. S2. Additional data on the role of APC-loss in CRC immune evasion and resistance to anti-PD-1 therapy. a, CT26 cells were transfected with shRNA targeting

Apc and the interference efficiency was detected by qRT-PCR and western blot. one-way ANOVA. **b,** CT26-scramble or CT26-shApc cells were injected into Balb/c and tumor weight was measured. *n* = 8, one-way ANOVA. **c,** Representative images of immunofluorescent staining against CD8 (yellow) and DAPI (blue) in indicated tumor tissues. **d, e,** Representative immunofluorescence staining of CK (purple), CD3 (red) and CD8 (green) in APC-WT and APC-mutation MSI-H **(d)** and MSS **(e)** colorectal cancer. **f, g,** Average number of $CD3^+$ and $CD8^+$ T cells were counted within 5 different fields (1.25 mm × 0.70 mm) and scatter plots show number of $CD3^+$ and $CD8^+$ cell in the tumor tissue of patients with MSI-H **(f)** and MSS+MSI-L (**e**, log2 normalized**)** colorectal cancer based on APC-WT and APC-mutation grouping. unpaired *t* test. **h,** Forest plot showing regression coefficient beta and 95% CI of MSI status and APC mutation to CD3E and CD8A. Linear regression analysis. **i,** CT26-scramble or CT26-shApc cells were injected into nude mice and tumor weight was measured. *n* = 8, one-way ANOVA. **j, k,** Indicated CT26 cells were transplanted into Balb/c mice injected with isotype or anti-CD8 antibody and tumor growth was monitored. two-way ANOVA. **l,** MC38 cells were transfected with shRNA targeting Apc and the interference efficiency was detected by qRT-PCR and western blot. one-way ANOVA. **m, n,** C57BL/6 mice were injected with MC38 cells transfected with scramble shRNA or two independent shRNAs targeting Apc, and tumor growth was monitored at the indicated times **(m)**. *n* = 8 tumors for each group. two-way ANOVA. Tumor weight was measured **(n)**. *n* = 8, one-way ANOVA. **o,** Representative immunofluorescence analysis of $CD8^+$ cells found in MC38 tumors and scatter plots showing number and $CD8^+$ cells in all three tumor tissues. *n* = 8 for each group, one-way ANOVA. **p**, **q**, $Rag1^{-/-}$ mice were injected with MC38 cells transfected with scramble shRNA or two independent shRNAs targeting Apc, and tumor growth was monitored at the indicated times **(p)**. *n* = 8 tumors for each group. two-way ANOVA. Tumor weight was measured **(q)**. *n* = 8, one-way ANOVA. **r,** The number of IFN-γ^+ or TNF-α^+ $CD8^+$ T cells in MC38-OVA-shAPC subcutaneous tumors were measured by flow cytometry. *n* = 3 for each group, one-way ANOVA. **s,** Indicated cells were transplanted into mice and tumor weight was measured. unpaired *t* test. $^*P < 0.05$, $^{**}P < 0.01$, $^{***}P < 0.001$, ns, no significance.
