# Supplementary material for: Targeting PTPN13 with 11-amino-acid peptides of C-terminal APC prevents immune evasion of colorectal cancer
Source: Cell Res. 2026 Jan 5;36(1):72–93. doi: 10.1038/s41422-025-01206-4 (PMC12765898; doi:10.1038/s41422-025-01206-4)
Supplement: Supplementary file 3 — Supplementary Figure S3 [file 41422_2025_1206_MOESM3_ESM.pdf]

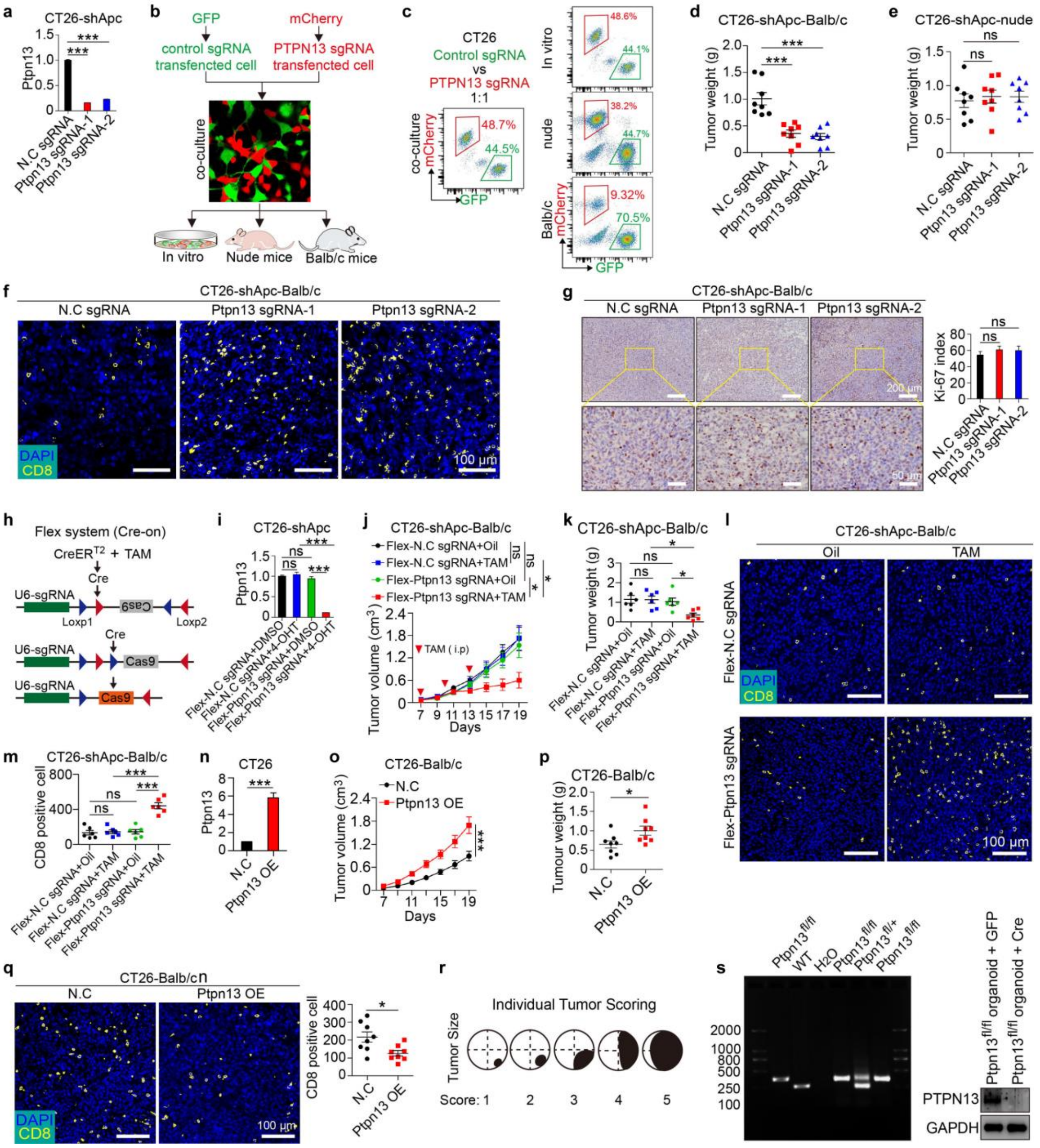

Supplementary information, Fig. S3. Additional data on the role of PTPN13 in APC-loss induced immune evasion. a, Knockout efficiency in indicated cells was detected using qRT-PCR. Data are calculated of three independent experiments, one-way ANOVA. **b, c,** Ptpn13 knockout- mCherry and control-GFP CT26 cells were mixed *in vitro* and subcutaneously injected proportionally (1:1) into WT Balb/c or nude mice, and GFP/mCherry ratio *in vitro* and *in vivo* was validated through FACS. **d, e,** Scatter plots show tumor weight of indicated cells formed in Balb/c **(d)** and nude mice **(e)**. n = 8. one-way ANOVA. **f,** Immunofluorescent staining against CD8 in subcutaneous tumor in Balb/c mice. **g,** Representative images and quantification of immunohistochemistry staining against Ki-67 in indicated tumor tissues. *n* = 8 for each group, one-way ANOVA. **h,** Schematic illustration of in vivo Ptpn13 knockout system. **i**, Indicated CT26 cells were treated with 4-OHT or DMSO, and Ptpn13 expression was detected by qRT-PCR. **j,** Indicated CT26 cells were transplanted into Balb/c mice, tamoxifen or oil was

injected intraperitoneally, tumor growth was monitored at the indicated times. *n* = 6 for each group. Significance for tumor growth kinetics were calculated by two-way ANOVA test. **k**, Scatter plots show tumor weight of indicated cells formed in Balb/c mice. *n* = 6, one-way ANOVA. **l**, Immunofluorescent staining against CD8 in tumor tissues. **m**, Scatter plots show number of CD8 positive cell in the tumor tissue in **(l)**. one-way ANOVA. **n,** Ptpn13 was overexpressed by CRISPR/Cas9- based Synergistic Activation Mediator (SAM) system and its expression was detected by qRT-PCR. Data are calculated of three independent experiments. unpaired t test. **o,** Ptpn13 was overexpressed in CT26 cells by SAM system and indicated cells were injected subcutaneously into Balb/c mice and tumor growth was monitored. *n* = 8 for each group, two-way ANOVA. **p,** Scatter plots show tumor weight of indicated cells formed in Balb/c mice. *n* = 8, unpaired t test. **q,** Immunofluorescent staining against CD8 in Ptpn13-overexpressed tumor tissues. Scatter plots show number of CD8 positive cell in the tumor tissues. unpaired t test. **r**, Schematic illustration of the tumor scoring determined by the diameter of the colonic lumen occupied by the tumor. **s,** Offspring of Ptpn13 conditional knockout mice were screened by using PCR and knockout efficiency was detected by immunoblot. All data are mean ± s.e.m., *P < 0.05, **P < 0.01, ***P < 0.001.
