# Supplementary material for: Targeting PTPN13 with 11-amino-acid peptides of C-terminal APC prevents immune evasion of colorectal cancer
Source: Cell Res. 2026 Jan 5;36(1):72–93. doi: 10.1038/s41422-025-01206-4 (PMC12765898; doi:10.1038/s41422-025-01206-4)
Supplement: Supplementary file 4 — Supplementary Figure S4 [file 41422_2025_1206_MOESM4_ESM.pdf]

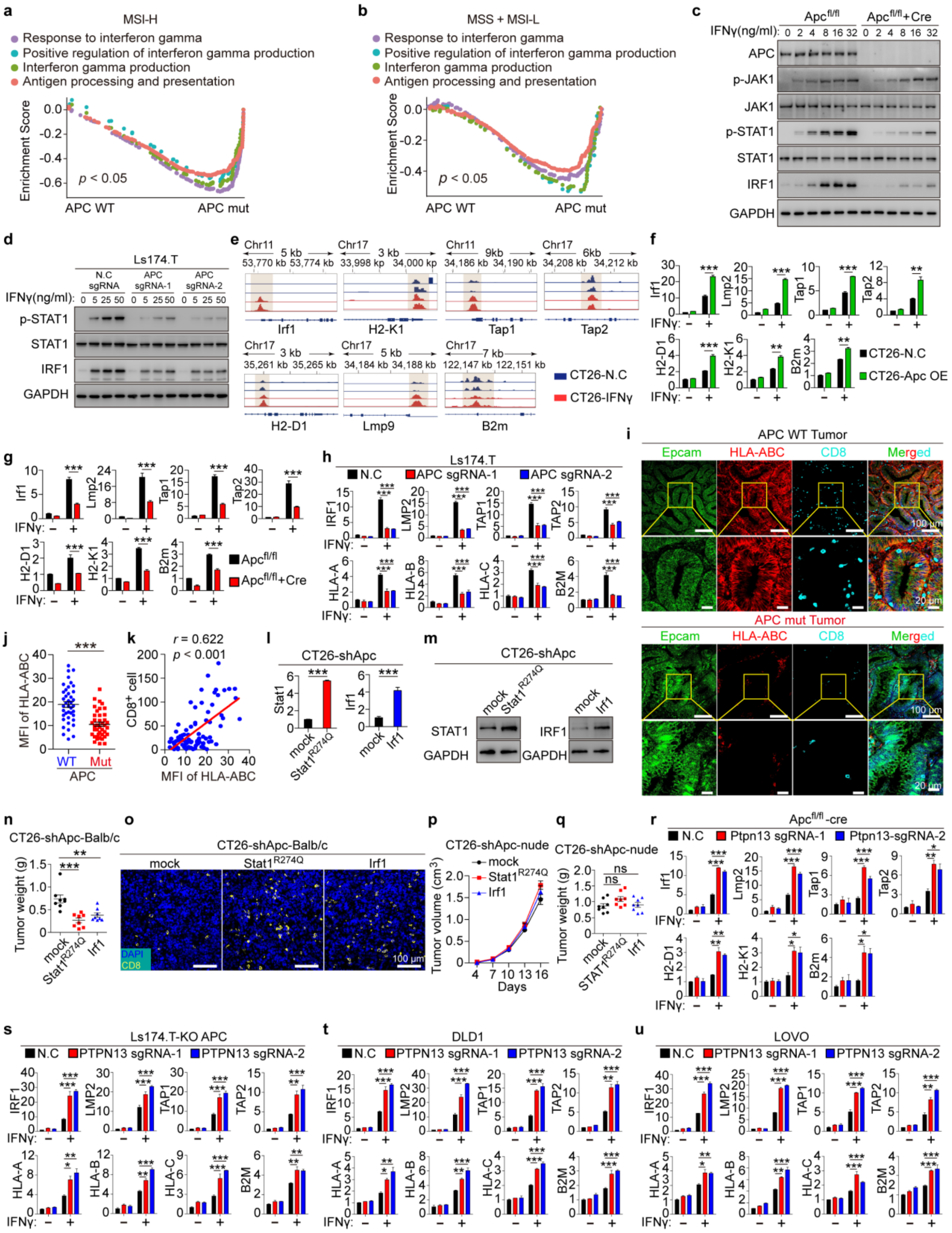

Supplementary information, Fig. S4. Additional data on the role of APC-loss in attenuating IFNγ-STAT1- IRF1-MHC-I antigen presentation signaling. a, b, GSEA using GO pathways were performed between APC mutated and APC WT groups respectively in MSI-H (A) and MSS+MSI-L (B) subgroup of TCGA dataset. **c,** Intestinal organoids from *Apc*$^{fl/fl}$ mice were cultured with adenovirus-GFP or adenovirus-Cre infection and stimulated with IFNγ with indicated concentrations for 2 h. Total cell lysates of indicated organoids were subjected to immunoblot analysis with antibodies to the indicated proteins. Data represents three independent experiments. **d,** Total cell lysates from gradient IFNγ concentration treated Ls174.T cell were subjected to immunoblot analysis with antibodies to the indicated proteins. Data represents three independent experiments. **e,** ChIP-Seq tracks of the Irf1, Psmb9,

Tap1, Tap2, H2-D1, H2-K1, B2m, Cxcl9, Cxcl10, and Cxcl11 gene loci of IFNγ (red) or PBS (blue) treated CT26 cells. Grey shaded regions are significantly differential between conditions. Two replicates shown per condition. **f-h,** Irf1, Lmp2, Tap1, Tap2, MHC-I and B2m mRNA expression (qRT-PCR) in indicated cells. Data were from three independent experiments. **i,** Representative immunofluorescence (IF) staining of Epcam (green), HLA-ABC (red) and CD8 (cyan) in APC-WT and APC-mutated CRC primary tissues. **j,** Mean fluorescence intensity (MFI) of HLA-ABC IF staining in APC-WT and APC-mutated CRC primary tissues. $n = 40$ per group, unpaired *t*-test. **k,** Scatter plot showing correlation between MFI of HLA-ABC and CD8 IF staining in APC-WT and APC-mutated CRC primary tissues. $n = 80$, Pearson's *r*. **l,** Expression of Stat1 and Irf1 in Apc-silenced CT26 cells transfected $Stat1^{R274Q}$ or Irf1 lentivirus was determined by qRT-PCR. Data represent three independent experiments, unpaired t test. **m,** Expression of Stat1 and Irf1 in Apc-silenced CT26 cells transfected $Stat1^{R274Q}$ or Irf1 lentivirus was determined by western blot. Data represent three independent experiments. **n,** Scatter plots show tumor weight of indicated cells formed in Balb/c mice. $n = 8$, one-way ANOVA. **o,** Representative immunofluorescence staining of CD8 in tumor tissues. **p**, Indicated cells were subcutaneously xenotransplanted to nude mice, tumor growth was monitored at indicated times. $n = 8$ for each group, two-way ANOVA. **q**, Scatter plots show tumor weight of indicated cells formed in nude mice. $n = 8$, one-way ANOVA. **r-u,** Irf1, Lmp2, Tap1, Tap2, MHC-I and B2m mRNA expression (qRT-PCR) in indicated cells. Data were from three independent experiments. All data are mean ± s.e.m., *P < 0.05, **P < 0.01, ***P < 0.001.
