# Supplementary material for: Targeting PTPN13 with 11-amino-acid peptides of C-terminal APC prevents immune evasion of colorectal cancer
Source: Cell Res. 2026 Jan 5;36(1):72–93. doi: 10.1038/s41422-025-01206-4 (PMC12765898; doi:10.1038/s41422-025-01206-4)
Supplement: Supplementary file 5 — Supplementary Figure S5 [file 41422_2025_1206_MOESM5_ESM.pdf]

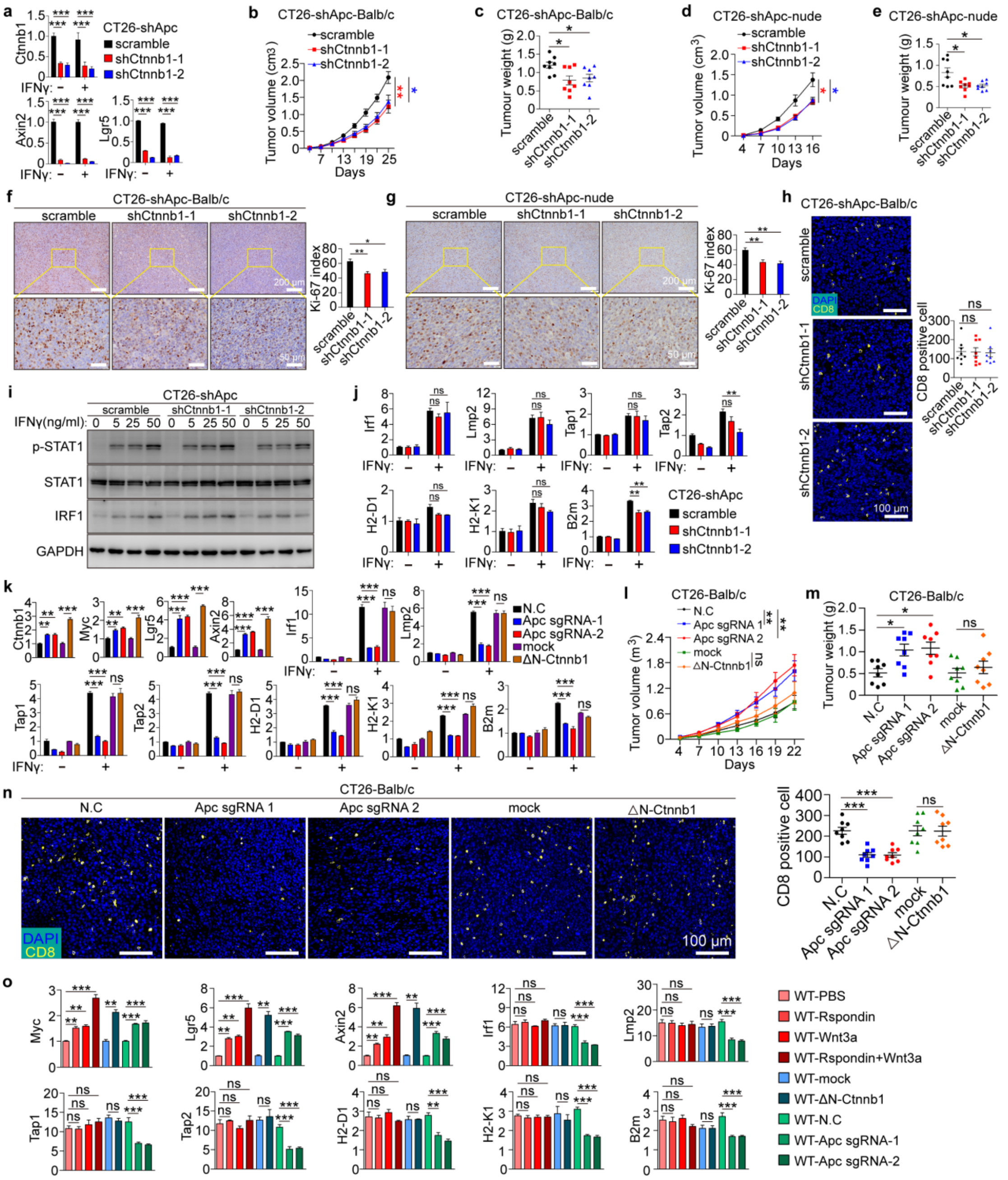

Supplementary information, Fig. S5. Role of β-catenin in APC-loss-induced CRC immune evasion and inactivation of IFNγ-STAT1-IRF1-MHC-I antigen presentation signaling. a, Ctnnb1, Axin2 and Lgr5 mRNA expression (qPCR) in indicated cells with 12 h exposure to IFNγ (50 ng ml^{-1}) before collection from three independent experiments. One-way ANOVA. **b-e,** Indicated cells were transplanted subcutaneously into Balb/c or nude mice. Tumor growth was monitored at indicated times**.** *n* = 8, two-way ANOVA. Tumor weight was measured. *n* = 8, one-way ANOVA. **f, g,** Representative images and quantification of immunohistochemistry staining against Ki-67 in tumor tissues. *n* = 8 for each group, one-way ANOVA. **h,** Frozen sections from indicated tumors were subjected to immunostaining analysis of CD8 (red) along with DAPI for DNA (cyan), and the number of $CD8^+$ cells were quantified. *n* = 8. one-way ANOVA. **i,** Cell lysates from gradient concentration IFNγ-stimulated cells were subjected to immunoblot analysis with antibodies to the indicated proteins. Data collected from three independent experiments. **j,** Irf1, Lmp2, Tap1, Tap2, H2-D1, H2K1 and B2m mRNA

expression (qPCR) in indicated cells with 12 h exposure to IFNγ (50 ng ml^{-1}) before collection from three independent experiments.one-way ANOVA. **k**, Apc was knocked out or ΔN-Ctnnb1 was overexpressed in CT26 cells, and Ctnnb1, Myc, Lgr5, Axin2, Irf1, Lmp2, Tap1, Tap2, H2-D1, H2K1 and B2m mRNA expression were determined by qPCR. Data were from three independent experiments. one-way ANOVA. **l**, Indicated CT26 cells were transplanted into Balb/c mice and tumor growth was monitored. Significance for tumor growth kinetics were calculated by two-way ANOVA test. **m**, Scatter plots show tumor weight of indicated cells formed in Balb/c mice. n = 8, one- way ANOVA. **n,** Representative immunofluorescence staining of CD8 in tumor tissues and scatter plots show number of $CD8^+$ cell in all three groups. n = 8, one-way ANOVA. **o,** Myc, Lgr5, and Axin2 were quantified by qPCR in organoids with indicated treatment, and Irf1, Lmp2, Tap1, Tap2, H2-D1, H2K1 and B2m mRNA expression were quantified by qPCR in indicated organoids with 12 h exposure to IFNγ (50 ng ml^{-1}) before collection from three independent experiments. one-way ANOVA.
