# Supplementary material for: Targeting PTPN13 with 11-amino-acid peptides of C-terminal APC prevents immune evasion of colorectal cancer
Source: Cell Res. 2026 Jan 5;36(1):72–93. doi: 10.1038/s41422-025-01206-4 (PMC12765898; doi:10.1038/s41422-025-01206-4)
Supplement: Supplementary file 6 — Supplementary Figure S6 [file 41422_2025_1206_MOESM6_ESM.pdf]

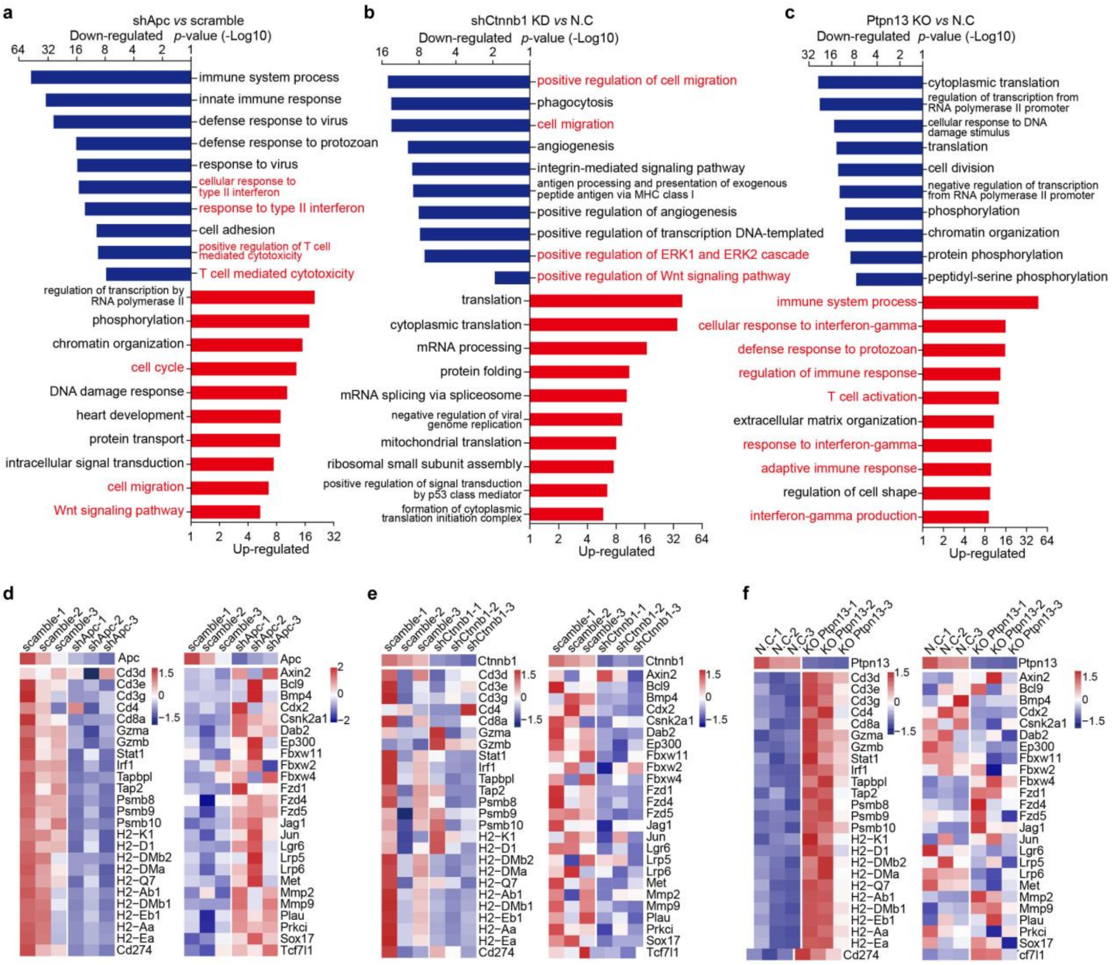

Supplementary information, Fig. S6. RNA-seq analysis of Apc-silenced, Ctnnb1-silenced and Ptpn13 knockout CT26 tumors. a-c, Bar chart showing up- and down-regulated GO pathway analyses in indicated tumors using The Database for Annotation, Visualization, and Integrated Discovery (DAVID), ordered by -log10(*p*-value). **d-f,** Heatmap showing comparison of immune-related markers (left) and Wnt target genes (right) in indicated tumors.
