# Supplementary material for: Targeting PTPN13 with 11-amino-acid peptides of C-terminal APC prevents immune evasion of colorectal cancer
Source: Cell Res. 2026 Jan 5;36(1):72–93. doi: 10.1038/s41422-025-01206-4 (PMC12765898; doi:10.1038/s41422-025-01206-4)
Supplement: Supplementary file 7 — Supplementary Figure S7 [file 41422_2025_1206_MOESM7_ESM.pdf]

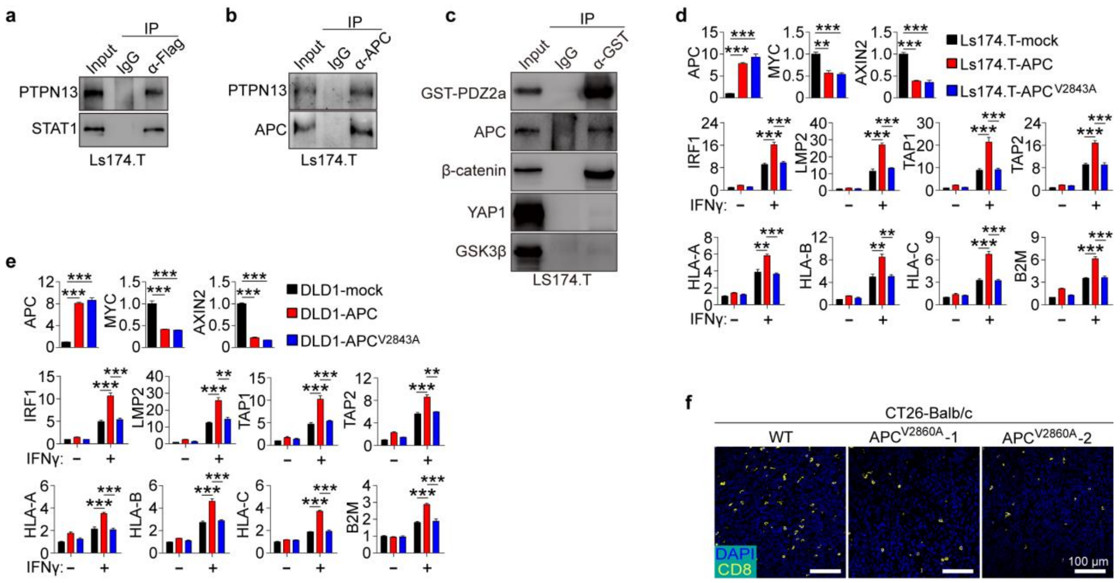

Supplementary information, Fig. S7. Additional data on the role of C-terminal valine of APC in CRC immune evasion. a, Flag-tagged Stat1 expression vector (0.5 μg) was transfected into Ls174.T cells. Total cell lysates were immunoprecipitated with anti-Flag and immunoblotted with anti- PTPN13. Data are representative of three independent experiments. **b,** Total cell lysates of Ls174.T cells were immunoprecipitated with anti-APC and immunoblotted with anti-PTPN13. Data are representative of three independent experiments. **c,** Total cell lysates of Ls174.T cells were incubated with GST-PDZ2a protein and immunoprecipitated with anti-GST and immunoblotted with anti-APC/β-catenin/YAP1/GSK3β. Data are representative of three independent experiments. **d, e,** 50 ng ml^{-1} IFNγ was administrated to APC-WT or APC^{V2843A} mutation plasmids transfected indicated cells for 12 h and IRF1, LMP2, TAP1, TAP2, MHC-I and B2M mRNA expression were detected by qRT-PCR. Data were from three independent experiments. One-way ANOVA. **f,** Representative immunofluorescence staining of CD8 in tumor tissues. Data represents three independent experiments. All data are mean ± s.e.m., **P* < 0.05, ***P* < 0.01, ****P* < 0.001, ns, not significant.
