# Supplementary material for: Targeting PTPN13 with 11-amino-acid peptides of C-terminal APC prevents immune evasion of colorectal cancer
Source: Cell Res. 2026 Jan 5;36(1):72–93. doi: 10.1038/s41422-025-01206-4 (PMC12765898; doi:10.1038/s41422-025-01206-4)
Supplement: Supplementary file 8 — Supplementary Figure S8 [file 41422_2025_1206_MOESM8_ESM.pdf]

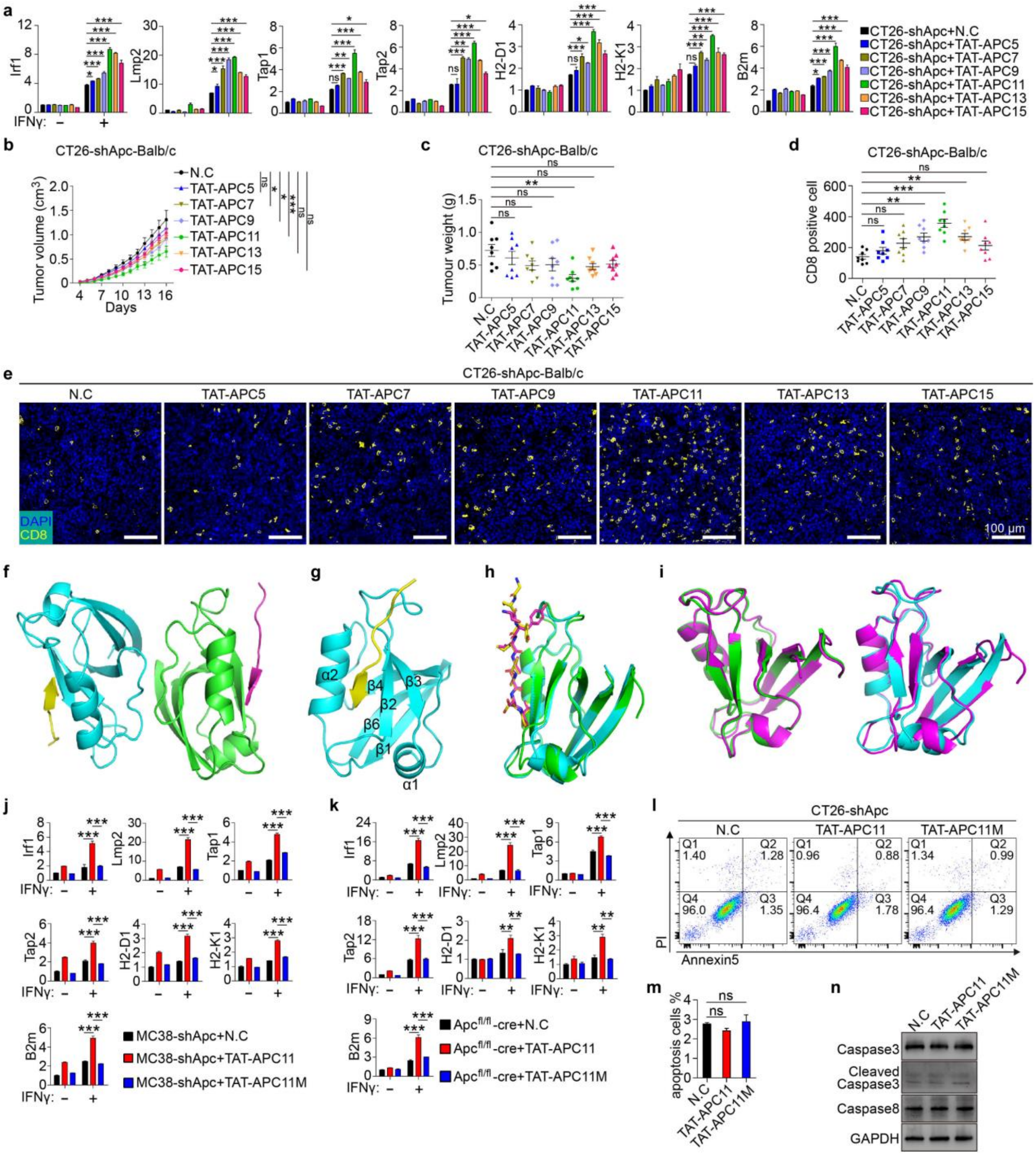

Supplementary information, Fig. S8. Additional data on the role of APC11 in restoration of IFNγ-STAT1-IRF1-MHC-I antigen presentation signaling. a, Irf1, Lmp2, Tap1, Tap2, H2-D1, H2K1 and B2m mRNA expression was determined by qPCR in Apc-silenced CT26 cell treated with indicated APC C-terminal peptides of different lengths for 2 h followed by IFNγ (50 ng ml^{-1}) stimulation for 12 h. Data were calculated of three independent experiments, one-way ANOVA. **b, c,** Apc-silenced CT26 cells were incubated with 50 μM indicated peptides for 4 h, cells were collected and suspended in PBS with 50 μM indicated peptides and injected subcutaneously (1×10^7 cells) into Balb/c mice and tumor growth was monitored **(b)**, n = 8, two-way ANOVA. Tumor weight were measured (**c**), n = 8, one-way ANOVA. **d,** Quantification of $CD8^+$ cells in indicated tumors, n = 8, one-way ANOVA. **e,** Representative immunofluorescence analysis of $CD8^+$ cells in indicated tumors. **f,** The overall structure of two PDZ2a-APC11 complexes in one asymmetric unit. **g,** PTPN13 PDZ2a domain (cyan) shows a typical PDZ folding topology with six β-strands (β1 to β6) and two α-helices (α1 and α2), and the C-terminus of APC peptide binds into the groove between β2 and α2 of PDZ2a. **h,** Superimposition of the two PDZ2a-APC11 complexes in each asymmetric unit. APC11 peptides are shown as sticks and

colored in yellow (last eight residues visible) or magenta (last seven residues). **i,** Structural comparison between apo (magenta, PDB ID: 3LNX) and two APC11 peptide-bound PDZ2a domains (green and cyan from each asymmetric unit). **j, k**, Irf1, Lmp2, Tap1, Tap2, H2-D1, H2K1 and B2m mRNA expression (qPCR) in indicated cells with 12 h exposure to IFNγ (50 ng ml^{-1}) before collection from three independent experiments. One-way ANOVA. **l**, **m**, Apc-silenced CT26 cells were incubated with 25 μM TAT-HA2 with or without 50 μM TAT-APC11 or TAT-APC11M for 2 h. Histogram represents FACS analysis and quantification of apoptotic cells. Data represent three independent experiments; two-way ANOVA. **n,** Apc-silenced CT26 cells were incubated with 25 μM TAT-HA2 with or without 50 μM TAT-APC11 or TAT-APC11M for 2 h. Caspase3, cleaved caspase3 and caspase8 were detected by western blot. Data represents three independent experiments. All data are mean ± s.e.m., $^*P < 0.05$, $^{**}P < 0.01$, $^{***}P < 0.001$, ns, not significant.
