# Supplementary material for: Targeting PTPN13 with 11-amino-acid peptides of C-terminal APC prevents immune evasion of colorectal cancer
Source: Cell Res. 2026 Jan 5;36(1):72–93. doi: 10.1038/s41422-025-01206-4 (PMC12765898; doi:10.1038/s41422-025-01206-4)
Supplement: Supplementary file 9 — Supplementary Figure S9 [file 41422_2025_1206_MOESM9_ESM.pdf]

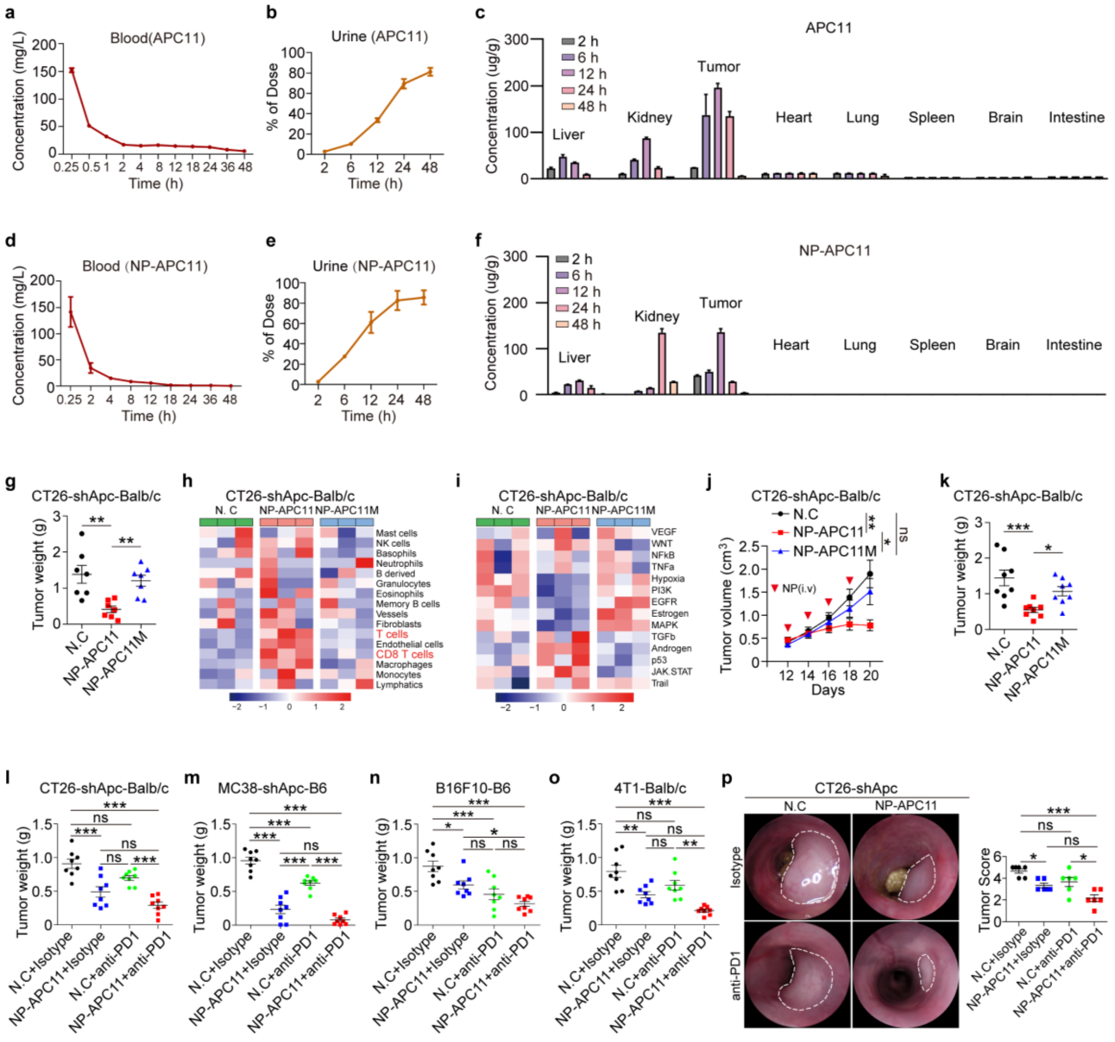

Supplementary information, Fig. S9. Additional data on the effect of APC11 on tumor immunotherapy. a, Serum concentration of FITC labeled APC11 peptide [iRGD-PEG2-(Lys)-FITC-PEG2-TSV] was measured by detecting relative fluorescence intensity of FITC in WT Balb/c after administration at indicated time points. Data was presented as relative fluorescence intensity to untreated mice at indicated time point. **b,** Urine concentration of FITC labeled APC11 peptide [iRGD-PEG2-(Lys)-FITC-PEG2-TSV] was measured by detecting relative fluorescence intensity of FITC in WT Balb/c after administration at indicated time points. **c,** Tissue targeting was assessed by detecting relative fluorescence intensity of FITC in brain, intestine, heart, lung, spleen, kidney, liver and tumor of FITC labeled APC11 peptide [iRGD-PEG2-(Lys)-FITC-PEG2-TSV] treated CT26-shAPC tumor bearing mice after administration at indicated time points. **d,** Serum concentration of Cy5 labeled NP-APC11 peptide [Cy5-iRGD-TSV-HA2] was measured by detecting relative fluorescence intensity of Cy5 in WT Balb/c after administration at indicated time points. **e,** Urine concentration of Cy5 labeled NP-APC11 peptide [Cy5-iRGD-TSV-HA2] was measured by detecting relative fluorescence intensity of Cy5 in WT Balb/c after administration at indicated time points. **f,** Tissue targeting was assessed by detecting relative fluorescence intensity of Cy5 in brain, intestine, heart, lung, spleen, kidney, liver and tumor of Cy5 labeled NP-APC11 peptide [Cy5-iRGD-TSV-HA2] treated CT26-shAPC tumor bearing mice after administration at indicated time points. **g,** Tumor weight in Figure 7F was measured. n = 8. **h,** Heatmap shows immune infiltration analysis (mMCPCounter) of

APC11 or APC11M treated CT26-shApc tumors. **i,** Heatmap shows pathway responsive genes for activity inference from gene expression (progeny) analysis of APC11 or APC11M treated CT26-shApc tumors. **j, k,** Apc-silenced CT26 cells were injected subcutaneously (1×10^7 cells) into Balb/c mice. Subsequently, 200 μg NP-APC11 or NP-APC11M was injected intraperitoneally every other day at indicated time when tumor reached 500 mm^3. Tumor growth (**j**) and weight (**k**) were measured. $n = 8$. **l-o**, Indicated cells were injected subcutaneously (1×10^7 cells) into mice, anti-PD-1 antibodies were injected intraperitoneally and NP-APC11 were injected via caudal vein. Tumor growth was monitered and tumor weight was measured. $n = 8$ for each group, two-way ANOVA for tumor volume, and one-way ANOVA for tumor weight. **p,** Apc-silenced CT26 cells were orthotopically inoculated to Balb/c mice, and anti-PD-1 antibodies were injected intraperitoneally and NP-APC11 were injected via caudal vein. Tumor growth were monitored and scored by colonoscopy. $n = 6$, one-way ANOVA.
