# Supplementary material for: Targeting PTPN13 with 11-amino-acid peptides of C-terminal APC prevents immune evasion of colorectal cancer
Source: Cell Res. 2026 Jan 5;36(1):72–93. doi: 10.1038/s41422-025-01206-4 (PMC12765898; doi:10.1038/s41422-025-01206-4)
Supplement: Supplementary file 10 — Supplementary Figure S10 [file 41422_2025_1206_MOESM10_ESM.pdf]

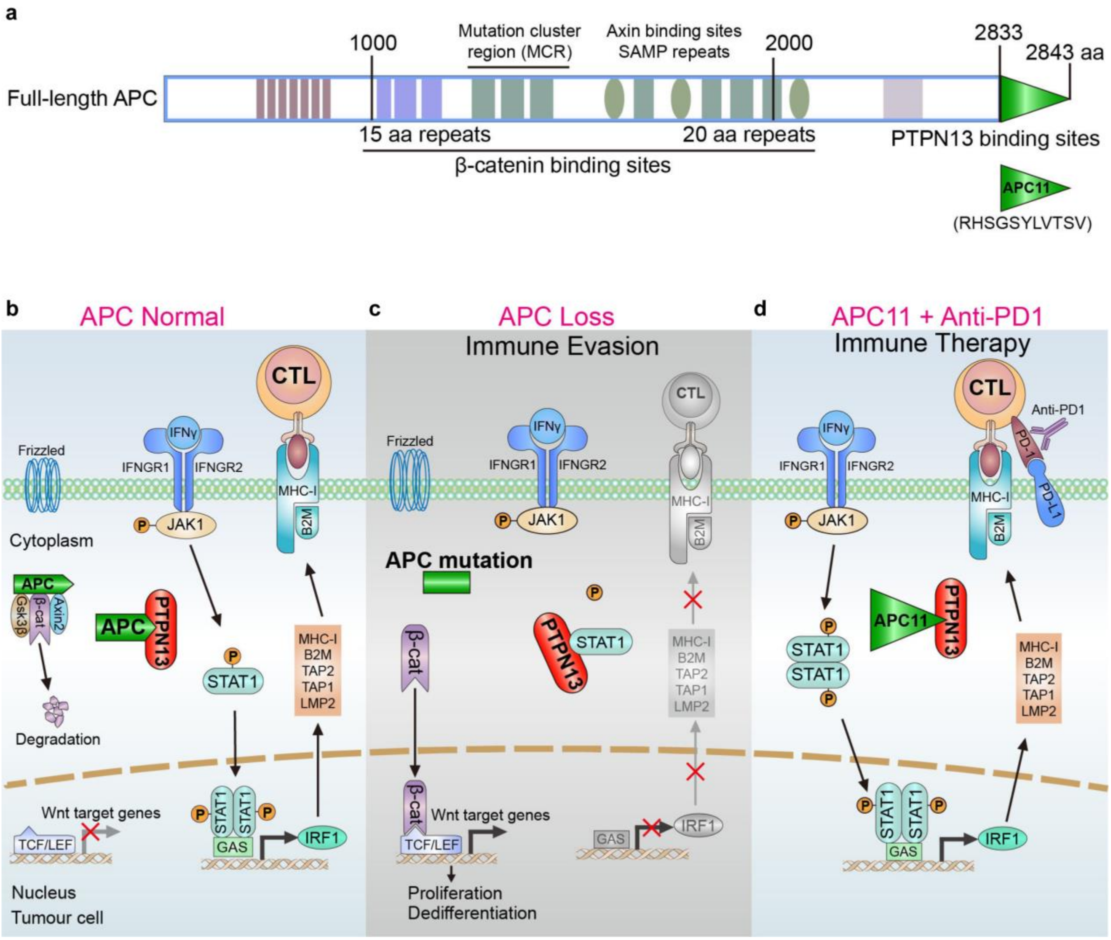

Supplementary information, Fig. S10. Diagram illustrating APC-loss-mediated tumor immune evasion. a, Diagram of the APC protein structure showing the regions relevant to Wnt signal transduction and PTPN13 interaction. **b,** In the presence of normal APC, APC is involved in destruction complex of β-catenin to inhibit Wnt/β-catenin target gene transcription; meanwhile, C-terminus of APC directly interacts with PTPN13, competitively inhibiting the interaction between PTPN13 and STAT1. This enhances STAT1 phosphorylation and activates the IFNγ-STAT1-IRF1-MHC class I antigen presentation pathway, which ultimately leads to increased $CD8^+$ T cell infiltration and suppression of tumor growth, independent of β-catenin. **c,** In the absence of APC (through genetic deletion or loss of function mutation), PTPN13 interacts with and dephosphorylates STAT1 to inhibit IFNγ-STAT1-IRF1-MHC class I antigen presentation pathway, leading to tumor immune evasion. **d,** The 11-aa peptide of C-terminal APC (APC11) binds to PTPN13, blocks PTPN13/STAT1 interaction to activate IFNγ-STAT1-IRF1-MHC-I antigen presentation signaling, overcomes tumor immune evasion, and synergizes with immune checkpoint blockade therapy.
