# Supplementary material for: Targeting PTPN13 with 11-amino-acid peptides of C-terminal APC prevents immune evasion of colorectal cancer
Source: Cell Res. 2026 Jan 5;36(1):72–93. doi: 10.1038/s41422-025-01206-4 (PMC12765898; doi:10.1038/s41422-025-01206-4)
Supplement: Supplementary file 12 — Supplementary Table S2 [file 41422_2025_1206_MOESM12_ESM.pdf]

Supplementary Table S2. Data collection and refinement statistics.

	PTPN13-PDZ2-APC
PDB code	7XTY
**Data collection**	
Space group	C121
Cell dimensions	
a, b, c (Å)	97.462, 32.407, 69.917
α, β, γ (°)	90.0, 108.857, 90.0
Resolution (Å)	33.14-2.10 (2.17-2.10)
R_{merge}	17.3 (37.4)
$I / \sigma I$	11.5 (2.67)
Completeness (%)	98.0 (87.4)
Redundancy	6.0 (4.2)
**Refinement**	
Resolution (Å)	2.1
No. reflections	12144
R_{work} / R_{free}	0.1935/0.2402
No. atoms	1563
Protein	1432
Water	131
B-factors	
Protein	26.94
water	27.28
R.m.s deviations	
Bond lengths (Å)	0.01
Bond angles (°)	1.25
Ramachandran plot residues in favored regions（%）	97.33 %

*One crystal was used for each structure.

*Values in parentheses are for the highest-resolution shell.
